# Molecular Periphery Design Allows Control of the New Nitrofurans Antimicrobial Selectivity

**DOI:** 10.3390/molecules29143364

**Published:** 2024-07-17

**Authors:** Lyubov Vinogradova, Alexey Lukin, Kristina Komarova, Maxim Zhuravlev, Artem Fadeev, Mikhail Chudinov, Elizaveta Rogacheva, Lyudmila Kraeva, Maxim Gureev, Yuri Porozov, Marine Dogonadze, Tatiana Vinogradova

**Affiliations:** 1 Lomonosov Institute of Fine Chemical Technologies, MIREA—Russian Technological University, 119454 Moscow, Russiafadeev19190@gmail.com (A.F.); 2 Pasteur Institute of Epidemiology and Microbiology, 197101 Saint Petersburg, Russia; 3 Institute of Cytology, Russian Academy of Sciences, Tikhoretsky Ave. 4, 194064 Saint Petersburg, Russia; 4 Laboratory of Angiopathology, The Institute of General Pathology and Pathophysiology, 8 Baltiyskaya Street, 125315 Moscow, Russia; 5 Advitam Laboratory, Mihaila Shushkaloviћа 13, 11030 Belgrade, Serbia; 6 Saint-Petersburg State Research Institute of Phthisiopulmonology of the Ministry of Healthcare of the Russian Federation, 191036 Saint Petersburg, Russia

**Keywords:** nitrofurans, ESKAPE, *M. tuberculosis*, antibacterial selectivity, nitroreductase, azoreductase, flexible docking, strained ligand–protein interactions

## Abstract

A series of 13 new 3-substituted 5-(5-nitro-2-furyl)-1,2,4-oxadiazoles was synthesized from different aminonitriles. All compounds were screened in the disc diffusion test at a 100 μg/mL concentration to determine the bacterial growth inhibition zone presence and diameter, and then the minimum inhibitory concentrations (MICs) were determined for the most active compounds by serial dilution. The compounds showed antibacterial activity against ESKAPE bacteria, predominantly suppressing the growth of 5 species out of the panel. Some compounds had similar or lower MICs against ESKAPE pathogens compared to ciprofloxacin, nitrofurantoin, and furazidin. In particular, 3-azetidin-3-yl-5-(5-nitro-2-furyl)-1,2,4-oxadiazole (**2h**) inhibited *S. aureus* at a concentration lower than all comparators. Compound **2e** (5-(5-nitro-2-furyl)-3-[4-(pyrrolidin-3-yloxy)phenyl]-1,2,4-oxadiazole) was active against Gram-positive ESKAPE pathogens as well as *M. tuberculosis*. Differences in the molecular periphery led to high selectivity for the compounds. The induced-fit docking (IFD) modeling technique was applied to in silico research. Molecular docking results indicated the targeting of compounds against various nitrofuran-associated biological targets.

## 1. Introduction

Antibiotic development efforts are most often focused on discovering broad-spectrum drugs that are simultaneously active against many pathogens. The undeniable advantages of these drugs also have a black side. The structure of biological targets varies significantly among different microbial species. As a result, broad-spectrum antibiotics aren’t able to combat various infections with equal efficiency. Such medications are often prescribed without accurately identifying the infection and are used off-label. This is the root of the antibiotic resistance problem [1], which makes antibiotic development economically unjustified [2]. An alternative to broad-spectrum antibiotics are so-called precision antibiotics. Precision antimicrobials selectively kill only one pathogen with the least off-target effects. This makes them less likely to cause resistance than broad-spectrum antibiotics. Additionally, since drugs that target only one pathogen do not use the same metabolic pathways, they are less likely to cause harm to the host’s resident microbes [3]. However, the search for antimicrobial agents that are selectively active against one pathogen seems even less economically reasonable.

The goal of our work was to demonstrate that excellent selectivity for a series of compounds against diverse microbial targets can be achieved by fine-tuning their molecular periphery. One of the research areas in our laboratory is new antimicrobials among nitro heterocycles, including nitrofurans [4,5] and nitroimidazoles [6]. Nitrofurans (NFs) have been used clinically since the 1960s. The antibiotic effect of NF follows a stepwise process involving activation by reduction, catalyzed by microbial azoreductases [7,8] and nitroreductases [9]. In anaerobic conditions, type I reductases catalyze the reduction of NFs to produce cytotoxic hydroxylamine. While in aerobic conditions, nitroreduction catalyzed by type II reductases produces reactive oxygen and nitrogen species, causing oxidative stress and the ultimate death of bacteria [10]. Specificity to certain reductases determines the selectivity of NF action. For these molecules, another type of action is also possible: the direct inhibition of target proteins [11,12].

**Figure 1:**
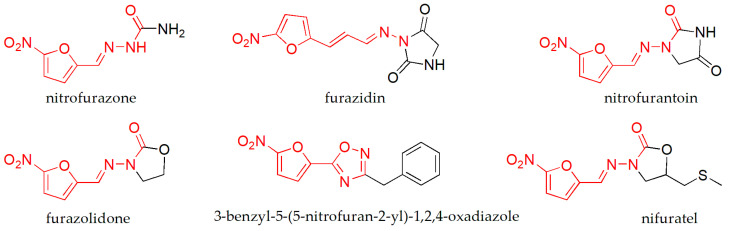
Some clinical nitrofuran-based antibiotics and most active antitubercular 5-(5-nitrofuran-2-yl)-1,2,4-oxadiazole (MIC = 3.1 μg/mL against *M. tuberculosis* HRv37 strain); nitrofuranoyl “warhead” marked as red.

Nitrofuran antibiotics are widely used in clinical practice against anaerobic infections. There are also many promising compounds based on the nitrofuran “warhead” that are active against *M. tuberculosis* (MTB) [10,13,14,15]. The classical NFs have a wide activity spectrum as well as high toxicity. Most clinical NFs (Figure 1) contain a “warhead” with an azo group derived from 5-nitro-2-furaldehyde. The previously described substitution of an azo group with an isosteric heterocycle can render these compounds non-cytotoxic in general, but it can also direct their activity to a specific type of bacterial target and does not affect the entire microbiome [5,14,15,16,17].

**Scheme 1:**
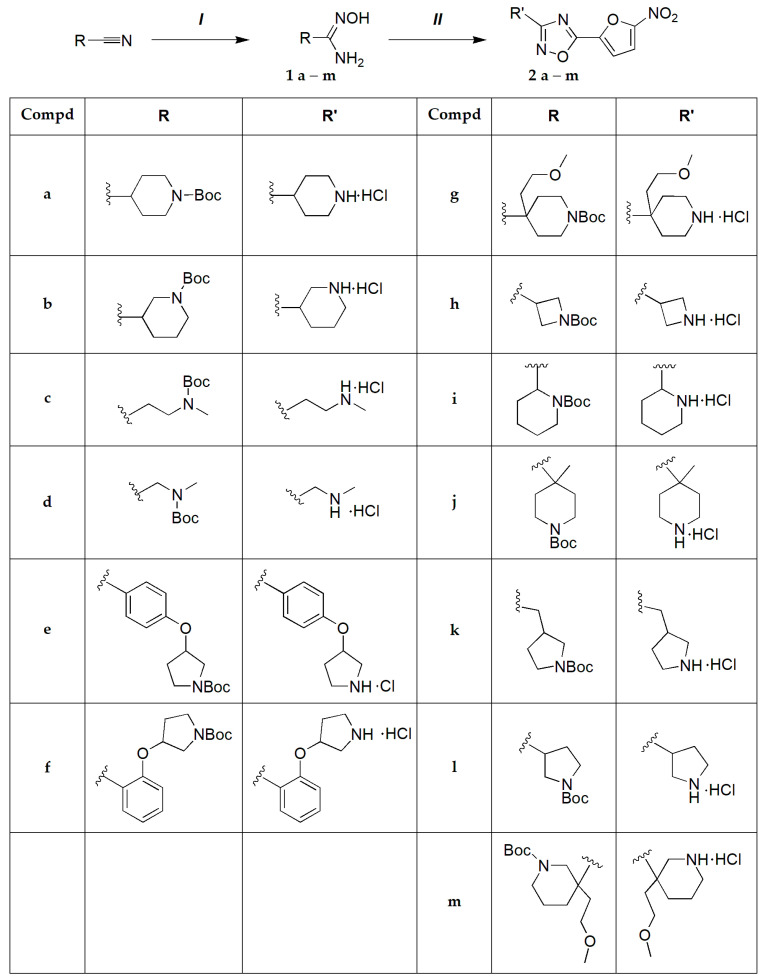
Synthesis of target compounds. Reagents and conditions: (*I*) NH_2_OH (50% aq), MeOH, rt, 12–24 h, 61–93%; (*II*) 5-nitro-2-furoic acid, CDI, DMF, 0 °C → 90 °C, then HCl (3 M, dioxane), 43–66%.

Thirteen new compounds were synthesized according to Scheme 1. In target compounds **2a**–**m**, the azo group was replaced by an isosteric oxadiazole ring. A range of alkylamine moieties were added to the previously tested design, which combined a 1,2,4-oxadiazole ring and a 5-nitrofuran pharmacophore. All new nitrofurans were tested against ESKAPE [18] and MTB pathogens. The previously reported conjugates of 1,2,4-oxadiazoles with nitrofurans (Figure 1) [15] displayed remarkable selectivity against MTB (HRv37 strain). However, their activity against other Gram-positive and Gram-negative pathogens was not observed. By contrast, certain compounds tested within the **2a**–**m** series shared structural features and demonstrated selectivity against individual pathogens of the ESKAPE panel and MTB. Molecular docking of these molecules with the most likely target proteins was used for this phenomenon explanation.

## 2. Results and Discussion

### 2.1. Chemistry

The synthesis of compounds **2a**–**m** was carried out in two steps using commercially available or previously synthesized nitriles [19,20,21]. At the first step, nitriles were treated with aqueous hydroxylamine to obtain amidoximes **1a**–**m**. Compounds **1a**–**m** were acylated with 5-nitro-2-furoic acid and cyclized one-pot into the target 3-substituted 5-(5-nitro-2-furyl)-1,2,4-oxadiazoles **2a**–**m**. The proposed cyclization method turned out to be the only effective way to obtain the target compounds. The other approaches described [22,23], such as cyclization of acylamidoximes in pyridine or catalysis with tetrabutyl ammonium fluoride, resulted in decomposition products only. The purity of the final compounds according to TLC and NMR data was sufficient for biological testing (at least 95%).

### 2.2. Antibacterial Activity

Nitrofurans **2a**–**m** were tested against Gram-positive (*S. aureus* and *E. faecium*) or Gram-negative (*P. aeruginosa*, *A. baumannii*, *K. pneumoniae*, and *E. cloacae*) pathogens of the ESKAPE panel. The clinically used antibiotic ciprofloxacin was used as a positive control and comparator. All compounds were screened at a 100 μg/mL concentration to determine the presence and diameter of the bacterial growth inhibition zone around the drug-treated disk (Table 1). Compounds **2c**,**h** that displayed growth inhibition for most pathogens on the ESKAPE panel were tested in serial dilution mode to determine the minimum inhibitory concentration (MIC). All compounds **2a**–**m** were tested against MTB in serial dilution mode, and MICs were determined (Table 2).

Most substances in the series exhibit antibacterial activity predominantly against Gram-positive bacteria (Table 1 and Table 2). Interestingly, they act on *S. aureus* but do not show an effect on *E. faecium.* Compound **2k** is active against *A. baumannii*, **2a** against *P. aeruginosa*, and **2b** against *E. cloacae.* Compound **2e** is active against *M. tuberculosis* and two Gram-positive and one Gram-negative pathogens.

### 2.3. Molecular Modeling

The calculations were performed for five targets belonging to four microbial species. Based on the results of experiments as well as data on the studied microorganisms, a pool of potential targets with which the studied compounds can interact was formed. These are azoreductase (AzoR), oxygen-insensitive NADPH nitroreductase (NfsA), oxygen-insensitive NAD(P)H nitroreductase (NfsB), deazaflavin-dependent nitroreductase from *M. tuberculosis* (Ddn), and phosphoglycerate kinase (Pgk). AzoR, NfsA, NfsB, and Ddn are conventional targets of nitrofuran antibiotics, as they reduce the nitrofuran moiety to highly reactive intermediates that inhibit the citric acid cycle as well as the synthesis of DNA, RNA, and proteins [25]. Pgk is not usually considered a possible target for nitrofurans, but there is an example of enzyme inhibition by a nitrofuran compound [26]. Phosphoglycerate kinase from *A. baumannii* has been selected as a potential target for antibiotic development [27]. It is worth noting that the protein structures were matched to the bacteria in question: *P. aeruginosa*, *S. aureus*, *A. baumannii*, and *M. tuberculosis.*

The docking results are summarized in Table 3, where a color-coded scheme of compound differentiation with experimentally derived characteristics of affinity or lack thereof in the context of site- and species-specific interactions is introduced. The low-active substances **2b**, **l**, and **m** were excluded from the calculations.

The experiments performed demonstrated that the compounds under study possess a number of remarkable properties. These properties have been interpreted through the utilization of molecular modeling techniques, enabling the identification of a priority target. This, in turn, illuminates the subsequent targeting of the compounds, taking into consideration both the nature of the target and species specificity.

**Figure 2:**
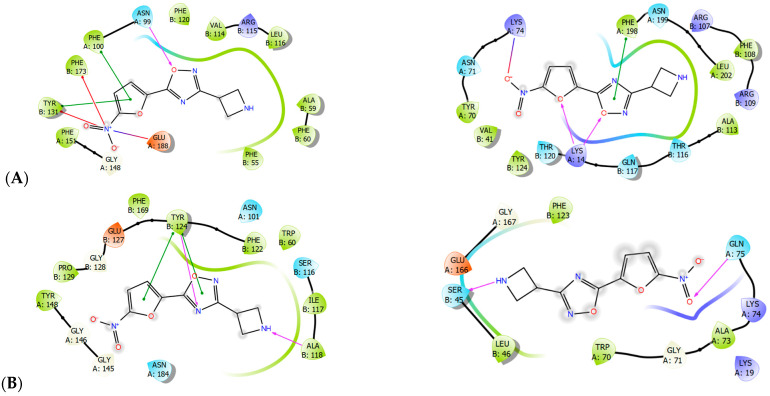

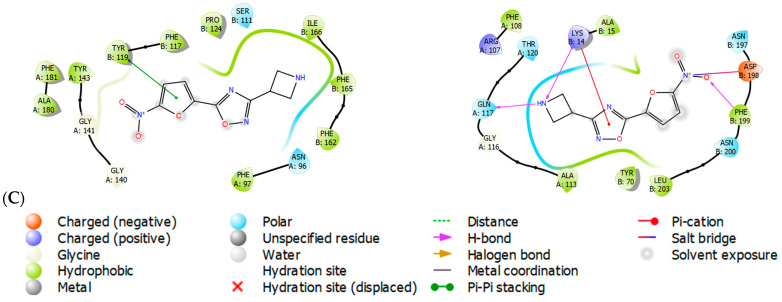
Ligand interactions diagrams of **2h** with AzoR (potential principal target) and NfsB (least likely target for ligand binding): left—AzoR, right—NfsB; (**A**) *P. aeruginosa*; (**B**) *S. aureus*; (**C**) *A. baumannii*.

Compounds **2c** and **2h** showed high antibacterial activity against ESKAPE pathogens but not antituberculosis activity. Compound **2c** showed high affinity for the Pgk protein in *P. aeruginosa* and *A. baumannii*, as well as for AzoR in *S. aureus* and *A. baumannii*. The interaction of **2c** with NfsA was moderate, despite the high quality of the binding pose, and the scores of the evaluation function are worse than those with AzoR. The affinity of **2c** to the NfsB protein was low, as was the quality of the binding pose. Apparently, the decrease in the intensity of lipophilic contacts causes the leaching, which in turn reduces the molecule’s affinity to the protein’s active site. Ligand interaction diagrams of **2c** with azoreductase (potential principal target) and NfsB (least likely target for ligand binding) are shown in Supporting Information (Figure S4). Compound **2h** demonstrated pronounced species specificity against *S. aureus*. These calculations predict the most effective interaction with azoreductase (AzoR) and oxygen-insensitive NADPH nitroreductase (NfsA). However, the calculations also showed that **2h** was capable of interacting with similar targets in other microorganisms considered. As in the previous case, NfsB was the least promising target for interaction (Table 3). The binding affinity of compound **2h** to azoreductase in all microorganisms was associated with tighter lipophilic contacts. Additionally, it is important to note the frequent occurrence of π-stacking between the furan and oxadiazole cycles with tyrosines and phenylalanines, which also aids in the proper orientation of the nitro group within the protein’s catalytic region (Figure 2).

**Figure 3:**
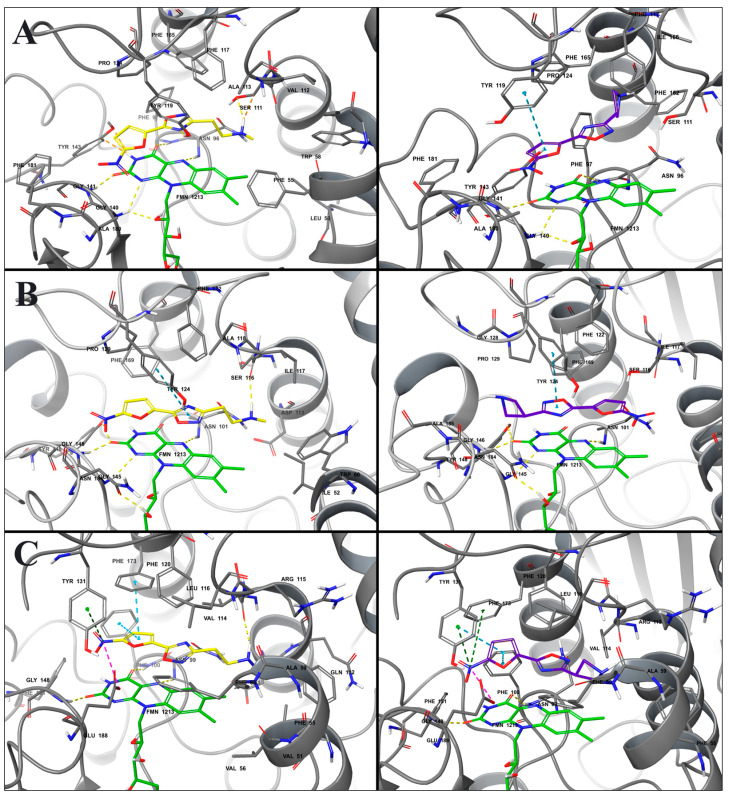
Co-factor accessibility for the nitro group in the AzoR structure of (**A**) *A. baumannii*; (**B**) *S. aureus*; (**C**) *P. aeruginosa*; (yellow-colored molecule—**2c**; violet—**2h**); FMN in green. Protein-ligand contacts showed by dashed lines: yellow—hydrogen bonds, light blue—pi-stacking, dark green—Pi-cation, magenta—salt bridge.

The active compounds **2c** and **2h** possess an orienting π-stacking interaction with Tyr119 of AzoR (Figure 2 and Figure 3), which ensures that the nitro group is present near the reactive flavin nitrogen of the mononucleotide (FMN). These compounds do not have the aforementioned drawbacks.

The next target is NfsA, which is present in all bacterial species. Nitrofurantoin is the main interactant and control compound. Calculations have shown that the leader compounds **2c** and **2h** have selective affinity to NfsA, depending on the pathogen. Compounds **2d** and **2g**, which did not show antibacterial activity against ESKAPE pathogens, demonstrated poor binding pose parameters and did not reproduce the key pharmacophore characteristics of the control compound.

Compound **2с** formed salt bridges with arginine and lysine in the same manner as the control and also had the necessary lipophilic contacts that determine the correct orientation of the ligand in the active site of the protein. Notably, **2c** had the highest affinity for NfsA from *A. baumannii*, followed by *S. aureus*. Based on the ligand–protein interaction diagrams, it is clear that **2c** has the advantage of an extended network of lipophilic contacts (see Supporting Information, Figure S8).

Compounds **2a**, **2c**, **2e**, **2f**, **2h**, **2i**, and **2j** showed pronounced species specificity against *S. aureus*. The above compounds have a rather high generalized affinity for the targets considered in the context of *S. aureus*. However, a variation in binding selectivity was observed in terms of binding pose quality and reproduction of pharmacophore characteristics. For example, binding to Pgk was not specific for all the above compounds due to their incorrect binding poses (Table 3, section Pgk@*S. aureus*).

In the context of *S. aureus* AzoR azoreductase, we observed high predicted affinity in terms of scoring function value as well as high binding pose quality (which corresponded to the pharmacophore characteristics for nitrofurantoin and other nitrofuran-based antibiotics) for compounds **2c**, **2f**, **2h**, and **2i**. The others, except the inactive ones, showed moderate binding pose quality with comparable scoring function values (see Table 3, section AzoR@*S. aureus*).

For NfsA of *S. aureus,* the picture was slightly different: compounds **2e** and **2f** were washed out due to poor quality protein–ligand binding. At the same time, the best-performing compounds were: **2a**, **2h**, and **2i**. They had the best binding pose and at the same time, high values of the scoring function (see Supporting Information, Table S4).

*S. aureus* phosphoglycerate kinase (Pgk) did not fall into the pool of potential targets of the compounds considered, as all compounds showed poor quality of docking solutions despite rather high values of predicted small molecule affinity to the active site (see Table 3, section Pgk@*S. aureus*).

In contrast, the situation with NfsB was positive: all the compounds listed showed high docking solution quality in the NfsB active cleft, with structures **2f**, **2i,** and **2k** as the best-fitting compounds.

**Figure 4:**
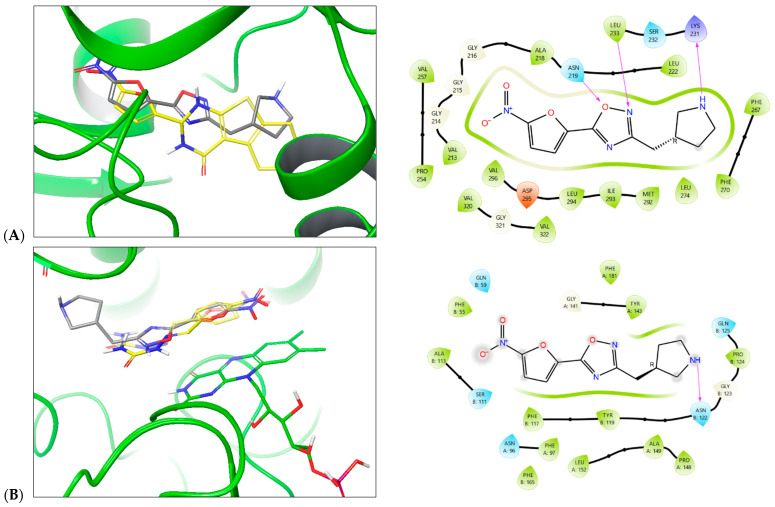

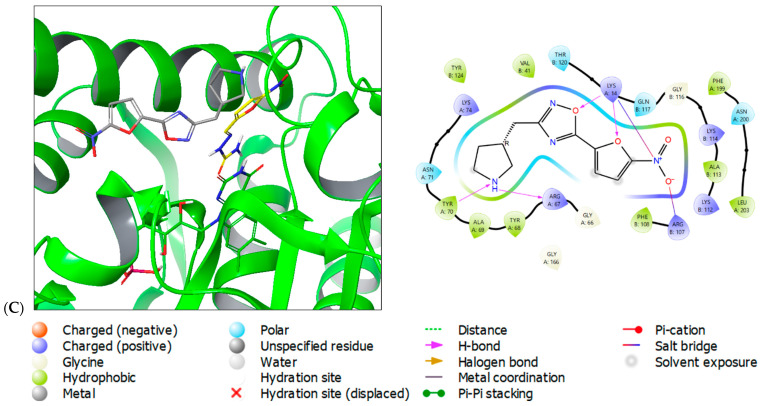
Interaction of **2k** with the most likely *A. baumannii* targets: left—three-dimensional binding poses models, right—ligand–protein interaction diagram; (**A**) **2k** with Pgk; (**B**) **2k** with AzoR; (**C**) **2k** with NfsB. In the three-dimensional models, the compound under study is shown in gray, the control compounds: **A**—2~{S})-2-(5-nitrofuran-2-yl)-2,3,5,6,7,8-hexahydro-1~{H}-[1]benzothiolo[2,3-d]pyrimidin-4-one [26]; **B**, **C**–nitrofurazone) is shown in yellow.

The selectivity of **2k** against *A. baumannii* is very interesting, since this pathogen is very important from the point of view of the treatment of nosocomial infections [28]. According to the combination of scoring function value and binding quality, the best target of **2k** was Pgk (Table 3). AzoR may serve as a second synergetic target. The binding to NfsB was energetically favorable but violated the control structure binding pose (Figure 4C).

In the case of interaction with Pgk, **2k** reproduced the binding mode of the reference compound with experimentally demonstrated affinity (see Supporting Information, Figure S2). The key was the orientation of the nitrofuran moiety towards the glycine-rich protein site (catalytic cavity of the protein) and the reproducibility of the necessary lipophilic contacts (Figure 4A).

Compound **2a**, selective against *P. aeruginosa*, had affinity to targets in the series: AzoR > Pgk > NfsA > NfsB (affinity level descending). The interaction was most qualitatively realized with AzoR and Pgk (in terms of reproducibility of the control structure binding mode as well as scoring function values, see Table 3). NfsA is rather a collateral target with low predicted affinity, although it can provide a potential synergistic effect due to a possible multi-target interaction.

**Figure 5:**
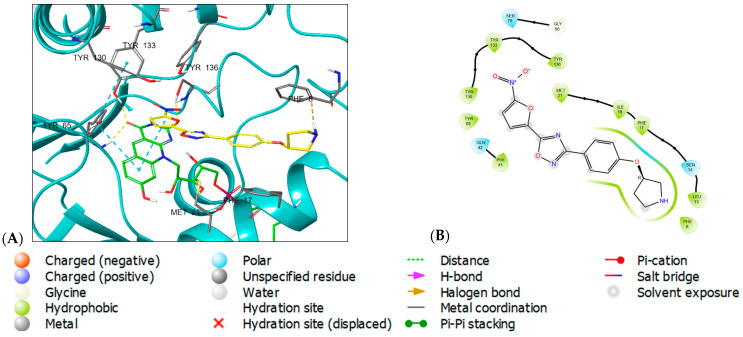
(**A**) Binding pose of **2e** (yellow) in Ddn active cavity; (**B**) ligand interactions diagram of **2e** with Ddn. Protein-ligand contacts showed on 3-d model with dashed lines: yellow—hydrogen bonds, light blue—pi-stacking, dark green—Pi-cation, magenta—salt bridge, orange—strained contact.

*M. tuberculosis* has its own specific targets, so it was decided to look at them separately and find the most likely ones. For the calculations, we used positive and negative controls as well as compound **2e** directly. Initially, only the structure of the Ddn protein (desaflavin-dependent nitroreductase) was considered. Compounds **2c**, **2d**, **2g,** and **2h** gave no docking results (the program reported that docking of this compound was not possible at the selected active site). On the other hand, the remaining structures showed a stable interaction with a high clustering of solutions, the best among them being **2e** and having the leading scoring function values (Table 3, section Ddn@*M. tuberculosis*). The **2e** nitrofuran group is located in the protein’s catalytic region, where the flavin mononucleotide (FMN) molecule is also present. This interaction with the nitro group determines the target activity during its reduction process. The nitrofuran functional group forms a π-stacking interaction with the aromatic core of FMN, contributing to the target activity. Additionally, the 3-phenoxypyrrolidine fragment at the oxadiazole core stimulates lipophilic contacts with leucines/isoleucines and phenylalanines in the protein’s active cavity (see Figure 5B).

However, *M. tuberculosis* has more promising targets than just nitroreductases. Analysis of the literature has revealed the presence of at least three additional targets:

(1) The HTH-type transcriptional regulator EthR interacts with linezolid, as indicated by Pdb: 5NZ0. Inhibition of EthR has been shown to enhance the effectiveness of antibiotics and reduce drug resistance [29];
(2) Arylamine N-acetyltransferase (TBNAT, Pdb: 4BGF) is responsible for the intracellular survival of *M. tuberculosis* within macrophages. It has been identified as a promising target [30,31]. In 2020, it was discovered that nitrofurans could inhibit it. Prior to this, there had been limited research on the subject [32].
(3) Enoyl reductase (InhA, PDB: 4TZK) is a protein associated with antibiotic resistance, making it an attractive target for small-molecule design [33,34].

All of the targets considered are present in the RCSB Protein Data Bank, some of them are in complex with small-molecule inhibitors (respectively, there is a control). After preparing the protein structures (error correction, solvent removal, GridBox calculation based on ligand or literature data), the investigated compounds were docked into the protein structure. These compounds included **2e** itself, active **2d**, **2k**, and **2h**, inactive **2a**, **2c**, and **2g**. The results are presented in Table 4.

**Figure 6:**
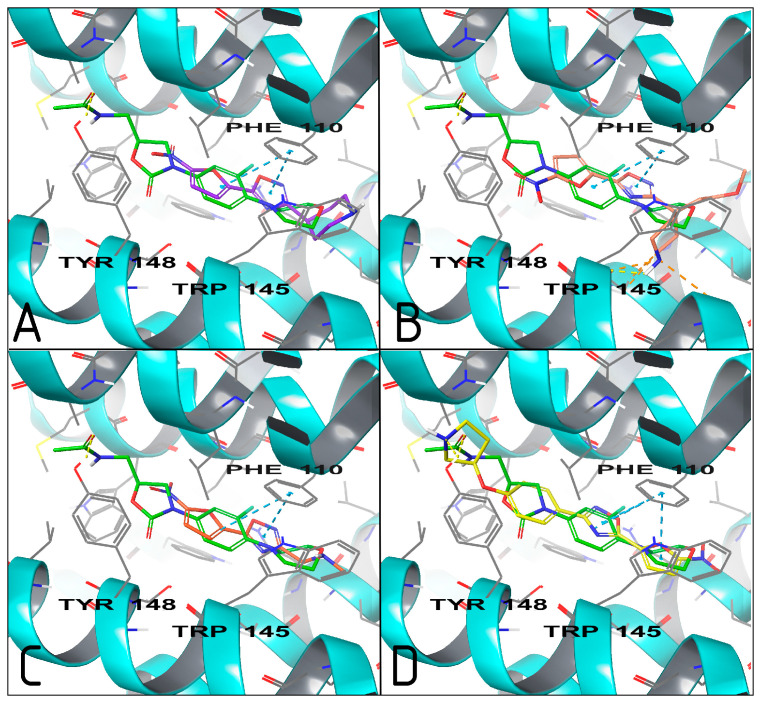
Binding poses of the structures studied in complex with EthR protein: (**A**) **2a** (violet); (**B**) **2g** (faded orange); (**C**) **2d** (orange); (**D**) **2e** (yellow). The control structure from PDB is shown in green. Protein-ligand contacts showed on 3-d model with dashed lines: yellow—hydrogen bonds, light blue—pi-stacking, dark green—Pi-cation, magenta—salt bridge, orange—strained contact.

Based on the modeling results, it can be concluded that EthR is the most likely target. This is because **2e** binds to the active cavity of the protein better than any other structure, as indicated by the scoring function. The lipophilic contacts with aromatic amino acids, including Phe110/184, Trp138/145, Leu183, and Ile107 (Figure 6), played a significant role in this binding. In all other compounds, the nitro group was oriented in the opposite direction (towards Tyr148). The absence of phenyl (as in **2e**) ensures redistribution of lipophilic contacts, resulting in a more rational binding mode (see Figure 6). This explains why **2e** had a higher predicted affinity to EthR compared to the other structures (see Table 4).

Regarding the InhA protein, all compounds, except **2g**, had high scores on the evaluation function that describes their potential affinity for the protein. However, we observed a leaching of **2g** in terms of binding pose quality. This was caused by steric factors, as the methoxyethyl substituent at the quaternary carbon of the piperidine fragment created steric complications for the binding mode of the compound in the same manner as the control (green). Compound **2d** was inactive and formed an unstable interaction, characterized by weak docking solutions clustering. All other compounds (**2a**, **2c**, **2e**, **2h**, and **2k**) binded identically. The nitro group was oriented similarly to the lipophilic dichlorophenyl fragment of the control compound, while the alicyclic fragments (and the aromatic one from **2e**) reproduced the binding mode of the cyclohexyl substituent of the control structure (see Figure S12). Compound **2e** was the leading candidate in the series, as it accurately reproduced the binding mode of the control compound and had the highest score.

No stable ligand–protein complexes were formed with TBNAT protein, and all structures exhibited low affinity for the active cavity. Therefore, TBNAT protein was not a priority target for compound **2e**, which had specific activity against *M. tuberculosis*.

## 3. Materials and Methods

### 3.1. Chemistry

All reactions were conducted on oven-dried glassware in a nitrogen atmosphere. Melting points were measured with a Buchi В-520 melting point apparatus (Buchi Labortechnik AG, Flawil, Switzerland) and were not corrected. The NMR spectra were recorded on a Bruker MSL-300 spectrometer (Bruker Corporation, MS, USA) at 25 °C (^1^H: 300 MHz; ^13^C: 75 MHz; chemical shifts are reported as parts per million (δ, ppm)) in dimethyl sulfoxide (DMSO-*d*_6_); the residual solvent peak used as an internal standard: 2.50 ppm for ^1^H and 40.01 ppm for ^13^C, respectively. Mass spectra were recorded using the Shimadzu LCMS-2020 system with ESI. High-resolution mass spectra (HRMS) were recorded using a Bruker microTOF spectrometer (Bruker Corporation, MS, USA) (ionization by electrospray, positive ion detection). Analytical thin-layer chromatography (TLC) was carried out on Sorbfil UV-254 silica gel plates (Imid Ltd., Moscow, Russia) using appropriate mixtures of solvent. The compounds were visualized with short-wavelength UV light. Column chromatography was performed on silica gel 60 (230–400 mesh). All reagents and solvents were obtained from commercial sources and used without further purification.

#### 3.1.1. General Procedure for the Synthesis of Compounds **1a**–**m**

To 5 mL of a 50% aqueous solution of NH_2_OH, the corresponding nitrile (0.01 mol) was added with stirring. MeOH was added dropwise to the resulting suspension until the precipitate was completely dissolved. After completion of the reaction (TLC control, 12–24 h), the precipitate that formed was filtered off and, if necessary, recrystallized from EtOH.

tert-butyl 4-[(hydroxyamino)(imino)methyl]piperidine-1-carboxylate (**1a**)

Yield 1.84 g (76%), white solid, m.p. 147–147.5 °С. ^1^H NMR (300 MHz, DMSO-*d*_6_) δ 8.78 (s, 1H), 5.28 (s, 2H), 3.94 (d, *J* = 13.0 Hz, 2H), 2.69 (t, *J* = 11.6 Hz, 2H), 2.26–2.02 (m, 1H), 1.66 (dd, *J* = 19.6, 8.9 Hz, 2H), 1.54–1.22 (m, 11H); ^13^C NMR (75 MHz, DMSO-*d*_6_) δ 155.61, 154.24, 78.88, 43.76, 38.57, 29.46, 28.50. LСMS (ESI): *m*/*z* (M + H^+^) calcd, 244.1; found, 244.2.

tert-butyl 3-[(hydroxyamino)(imino)methyl]piperidine-1-carboxylate (**1b**)

Yield 1.75 g (72%), white solid, m.p. 134–134.5 °С. ^1^H NMR (300 MHz, DMSO-*d*_6_) δ 8.85 (s, 1H), 5.32 (s, 2H), 4.00–3.80 (m, 2H), 2.91–2.55 (m, 2H), 2.05 (tt, *J* = 11.5, 3.7 Hz, 1H), 1.89 (d, *J* = 12.6 Hz, 1H), 1.69–1.57 (m, 1H), 1.55–1.22 (m, 12H); ^13^C NMR (75 MHz, DMSO-*d*_6_) δ 154.27, 78.94, 60.15, 55.26, 28.95, 28.44, 25.13, 21.12, 14.45. LСMS (ESI): *m*/*z* (M + H^+^) calcd, 244.1; found, 244.2.

tert-butyl [3-(hydroxyamino)-3-iminopropyl]methylcarbamate (**1c**)

Yield 1.7 g (78%), white solid, m.p. 150.5–151 °С. ^1^H NMR (300 MHz, DMSO-*d*_6_) δ 8.81 (s, 1H), 5.35 (s, 2H), 3.32 (t, *J* = 7.3 Hz, 2H), 2.76 (s, 3H), 2.14 (t, *J* = 7.3 Hz, 2H), 1.38 (s, 9H); ^13^C NMR (75 MHz, DMSO-*d*_6_) δ 155.00, 151.06, 78.67, 46.53, 34.27, 28.43. LСMS (ESI): *m*/*z* (M + H^+^) calcd, 218.1; found, 218.2.

tert-butyl [2-(hydroxyamino)-2-iminoethyl]methylcarbamate (**1d**)

Yield 10 g (92%), white solid, m.p. 173–173.5 °С. ^1^H NMR (300 MHz, DMSO-*d*_6_) δ 9.11 (s, 1H), 5.16 (s, 2H), 3.72 (s, 2H), 2.75 (s, 3H), 1.40 (s, 9H); ^13^C NMR (75 MHz, DMSO-*d*_6_) δ 149.69, 79.30, 48.77, 48.24, 34.43, 28.41. LСMS (ESI): *m*/*z* (M + H^+^) calcd, 204.1; found, 204.2.

tert-butyl 3-{4-[(hydroxyamino)(imino)methyl]phenoxy}pyrrolidine-1-carboxylate (**1e**)

Yield 1.95 g (61%), white solid, m.p. 198–198.5 °С. ^1^H NMR (300 MHz, DMSO-*d*_6_) δ 9.45 (s, 1H), 7.60 (d, *J* = 8.8 Hz, 2H), 6.93 (d, *J* = 8.8 Hz, 2H), 5.70 (s, 2H), 5.02 (s, 1H), 3.53 (s, 1H), 3.48–3.33 (m, 3H), 2.09 (d, *J* = 19.3 Hz, 2H), 1.39 (s, 9H); ^13^C NMR (75 MHz, DMSO-*d*_6_) δ 154.00, 153.91, 150.90, 127.24, 126.45, 115.28, 78.94, 76.48, 75.61, 51.51, 44.07, 31.12, 30.35, 28.51. LСMS (ESI): *m*/*z* (M + H^+^) calcd, 322.1; found, 322.2.

tert-butyl 3-{2-[(hydroxyamino)(imino)methyl]phenoxy}pyrrolidine-1-carboxylate (**1f**)

Yield 2.08 g (65%), white solid, m.p. 184–184.5 °С. ^1^H NMR (300 MHz, DMSO-*d*_6_) δ 9.43 (s, 1H), 7.43 (dd, *J* = 7.6, 1.7 Hz, 1H), 7.35 (ddd, *J* = 8.4, 7.4, 1.8 Hz, 1H), 7.07 (d, *J* = 8.1 Hz, 1H), 6.97 (td, *J* = 7.5, 0.9 Hz, 1H), 5.52 (s, 2H), 5.02 (s, 1H), 3.55 (s, 1H), 3.50–3.22 (m, 3H), 2.08 (s, 2H), 1.40 (s, 9H); ^13^C NMR (75 MHz, DMSO-*d*_6_) δ 155.10, 154.01, 151.18, 130.54, 123.96, 121.17, 114.70, 78.91, 77.51, 76.66, 51.67, 44.33, 31.26, 30.48, 28.53. LСMS (ESI): *m*/*z* (M + H^+^) calcd, 322.1; found, 322.2.

tert-butyl 4-[(hydroxyamino)(imino)methyl]-4-(2-methoxyethyl)piperidine-1-carboxylate (**1g**)

Yield 2.1 g (70%), white solid, m.p. 120–120.5 °С. ^1^H NMR (300 MHz, DMSO-*d*_6_) δ 9.06 (s, 1H)-слабый, 7.09 (d, *J* = 76.3 Hz, 1H), 5.31 (s, 1H), 3.71–3.45 (m, 2H), 3.31 (s, 1H), 3.25 (q, *J* = 7.6 Hz, 2H), 3.17 (d, *J* = 2.5 Hz, 3H), 3.08–2.75 (m, 2H), 1.94 (d, *J* = 13.7 Hz, 2H), 1.69 (t, *J* = 7.3 Hz, 2H), 1.38 (s, 9H), 1.27 (t, *J* = 10.4 Hz, 2H); ^13^C NMR (75 MHz, DMSO-*d*_6_) δ 176.66, 154.43, 154.31, 154.03, 78.85, 68.66, 58.26, 43.36, 33.39, 28.44. LСMS (ESI): *m*/*z* (M + H^+^) calcd, 302.2; found, 302.2.

tert-butyl 3-[(hydroxyamino)(imino)methyl]azetidine-1-carboxylate (**1h**)

Yield 2.27 g (72%), white solid, m.p. 170–170.5 °С. ^1^H NMR (300 MHz, DMSO-*d*_6_) δ 9.07 (s, 1H), 5.43 (s, 2H), 3.91 (dd, *J* = 9.9, 7.0 Hz, 4H), 3.24–3.09 (m, 1H), 1.37 (s, 9H); ^13^C NMR (75 MHz, DMSO-*d*_6_) δ 155.93, 152.28, 78.90, 55.23, 29.57, 28.42. LСMS (ESI): *m*/*z* (M + H^+^) calcd, 316.1; found, 316.2.

tert-butyl 2-[(hydroxyamino)(imino)methyl]piperidine-1-carboxylate (**1i**)

Yield 2.26 g (93%), white solid, m.p. 154–154.5 °С. ^1^H NMR (300 MHz, DMSO-*d*_6_) δ 9.13 (s, 1H), 5.14 (s, 2H), 4.64 (s, 1H), 3.87–3.67 (m, 1H), 2.96 (t, *J* = 12.4 Hz, 1H), 2.03 (t, *J* = 12.1 Hz, 1H), 1.67–1.07 (m, 14H); ^13^C NMR (75 MHz, DMSO-*d*_6_) δ 155.09, 151.39, 79.07, 51.07, 41.33, 28.48, 26.68, 25.07, 19.81. LСMS (ESI): *m*/*z* (M + H^+^) calcd, 244.1; found, 244.2.

tert-butyl 4-[(hydroxyamino)(imino)methyl]-4-methylpiperidine-1-carboxylate (**1j**)

Yield 1.72 g (67%), white solid, m.p. 178.5–179 °С. ^1^H NMR (300 MHz, DMSO-*d*_6_) δ 8.97 (s, 1H), 5.26 (s, 2H), 3.47 (dt, *J* = 9.2, 4.3 Hz, 2H), 3.10 (t, *J* = 10.3 Hz, 2H), 1.88 (t, *J* = 16.7 Hz, 2H), 1.38 (s, *J* = 4.9 Hz, 9H), 1.22 (ddd, *J* = 14.6, 9.1, 4.3 Hz, 2H), 1.10 (s, 3H); ^13^C NMR (75 MHz, DMSO-*d*_6_) δ 156.48, 154.46, 109.53, 78.76, 36.73, 34.42, 28.50, 26.83. LСMS (ESI): *m*/*z* (M + H^+^) calcd, 258.1; found, 258.2.

tert-butyl 3-[2-(hydroxyamino)-2-iminoethyl]pyrrolidine-1-carboxylate (**1k**)

Yield 1.58 g (65%), white solid, m.p. 92–92.5 °С. ^1^H NMR (300 MHz, DMSO-*d*_6_) δ 8.76 (s, 1H), 5.34 (s, 2H), 3.34 (tdd, *J* = 12.1, 9.3, 3.8 Hz, 2H), 2.84 (dd, *J* = 10.5, 7.7 Hz, 1H), 2.36 (dd, *J* = 29.8, 6.6 Hz, 1H), 2.16–1.73 (m, 3H), 1.63–1.42 (m, 1H), 1.38 (s, 9H); ^13^C NMR (75 MHz, DMSO-*d*_6_) δ 153.92, 152.10, 78.52, 78.46, 51.22, 50.99, 48.97, 45.46, 45.33, 36.35, 35.51, 34.41, 31.24, 30.56, 28.56. LСMS (ESI): *m*/*z* (M + H^+^) calcd, 244.1; found, 244.2.

tert-butyl 3-[(hydroxyamino)(imino)methyl]pyrrolidine-1-carboxylate (**1l**)

Yield 1.92 g (84%), white solid, m.p. 158–158.5 °С. ^1^H NMR (300 MHz, DMSO-*d*_6_) δ 8.94 (s, 1H), 5.40 (s, 2H), 3.53–3.03 (m, 4H), 2.78 (dd, *J* = 15.1, 7.6 Hz, 1H), 2.07–1.78 (m, 2H), 1.39 (s, 9H); ^13^C NMR (75 MHz, DMSO-*d*_6_) δ 153.81, 152.52, 152.47, 78.52, 48.76, 48.56, 45.61, 45.45, 29.16, 28.55, 28.39, 19.31. LСMS (ESI): *m*/*z* (M + H^+^) calcd, 230.1; found, 230.2.

tert-butyl 3-[(hydroxyamino)(imino)methyl]-3-(2-methoxyethyl)piperidine-1-carboxylate (**1m**)

Yield 2.41 g (80%), white solid, m.p. 104–104.5 *°*С. ^1^H NMR (300 MHz, *DMSO*-d_6_) δ 9.01 (s, 1H), 5.29 (s, 2H), 3.64 (s, 2H), 3.39–3.23 (m, 3H), 2.81 (s, 1H), 1.91–1.56 (m, 5H), 1.54–1.30 (m, 12H); ^13^C NMR (75 MHz, *DMSO*-d_6_) δ 155.27, 154.35, 78.89, 69.22, 58.25, 49.07. LСMS (ESI): *m*/*z* (M + H^+^) calcd, 302.2; found, 302.2.

#### 3.1.2. General Procedure for the Synthesis of Compounds **2a**–**m**

To a solution of 5-nitro-2-furoic acid (0.66 g, 0.42 mmol) in DMF (10 mL) at 0 °C, CDI (0.71 g, 0.44 mmol) was added in portions with stirring. The reaction mixture was stirred for 30 min, and then a solution of 0.4 mmol of the corresponding amidoxime in DMF (5 mL) was added dropwise. The reaction mass was stirred overnight at room temperature, then heated to 90 °C and stirred for 4 h. The reaction mixture was poured onto ice, and the precipitate that formed was filtered off and dried. Then 5 mL of a 3 M HCl/dioxane solution was added to the resulting precipitate, stirred overnight, evaporated, and the residue was crystallized from EtOH.

4-[5-(5-nitro-2-furyl)-1,2,4-oxadiazol-3-yl]piperidine hydrochloride (**2a**)

Yield 0.6 g (50%), white solid, m.p. 176–176.5 °С. ^1^H NMR (300 MHz, DMSO-*d*_6_) δ 8.99 (s, 2H), 7.96 (d, *J* = 4.0 Hz, 1H), 7.89 (d, *J* = 4.0 Hz, 1H), 3.07 (t, *J* = 11.7 Hz, 2H), 2.17 (dd, *J* = 14.1, 2.8 Hz, 2H), 2.06–1.88 (m, 2H); ^13^C NMR (75 MHz, DMSO-*d*_6_) δ 173.00, 166.00, 153.38, 140.29, 119.43, 114.20, 42.50, 31.12, 26.20. HRMS (ESI), *m*/*z* calcd C_11_H_13_N_4_O_4_ [M + H^+^] 265.0936, found 265.0936.

3-[5-(5-nitro-2-furyl)-1,2,4-oxadiazol-3-yl]piperidine hydrochloride (**2b**)

Yield 0.7 g (59%), white solid, m.p. 166–166.5 °С. ^1^H NMR (300 MHz, DMSO-*d*_6_) δ 9.43 (s, 2H), 7.96 (d, *J* = 3.9 Hz, 1H), 7.91 (d, *J* = 4.0 Hz, 1H), 3.61–3.44 (m, 2H), 3.28 (d, *J* = 11.5 Hz, 1H), 3.15 (s, 1H), 2.94 (s, 1H), 2.15 (d, *J* = 12.4 Hz, 1H), 1.85 (d, *J* = 30.5 Hz, 3H), 1.20 (t, *J* = 7.2 Hz, 1H); ^13^C NMR (75 MHz, DMSO-*d*_6_) δ 171.19, 166.09, 153.43, 140.13, 45.76, 45.16, 43.21, 31.45, 26.52, 21.37, 8.82. HRMS (ESI), *m*/*z* calcd C_11_H_13_N_4_O_4_ [M + H^+^] 265.0936, found 265.0935.

*N*-methyl-2-[5-(5-nitro-2-furyl)-1,2,4-oxadiazol-3-yl]ethanamine hydrochloride (**2c**)

Yield 0.61 g (56%), white solid, m.p. 178.5–179 °С. ^1^H NMR (300 MHz, DMSO-*d*_6_) δ 9.26 (s, 2H), 7.96 (d, *J* = 4.0 Hz, 1H), 7.91 (d, *J* = 4.0 Hz, 1H), 3.45–3.21 (m, 4H), 2.59 (t, *J* = 4.8 Hz, 3H); ^13^C NMR (75 MHz, DMSO-*d*_6_) δ 168.32, 166.08, 153.44, 140.14, 119.57, 114.19, 45.43, 34.53, 32.74, 22.77. HRMS (ESI), *m*/*z* calcd C_9_H_11_N_4_O_4_ [M + H^+^] 239.0780, found 239.0782.

*N*-methyl-1-[5-(5-nitro-2-furyl)-1,2,4-oxadiazol-3-yl]methanamine hydrochloride (**2d**)

Yield 0.55 g (53%), white solid, m.p. 229–129.5 °С. ^1^H NMR (300 MHz, DMSO-*d*_6_) δ 9.99 (s, *J* = 68.9 Hz, 1H), 7.97 (dd, *J* = 7.7, 3.8 Hz, 2H), 4.52 (s, 2H), 2.68 (s, 3H); ^13^C NMR (75 MHz, DMSO-*d*_6_) δ 166.64, 165.27, 153.59, 139.68, 120.18, 114.19, 42.31, 33.15. HRMS (ESI), *m*/*z* calcd C_8_H_9_N_4_O_4_ [M + H^+^] 225.0623, found 225.0623.

5-(5-nitro-2-furyl)-3-[4-(pyrrolidin-3-yloxy)phenyl]-1,2,4-oxadiazole hydrochloride (**2e**)

Yield 0.7 g (46%), white solid, m.p. 162–163.5 °С. ^1^H NMR (300 MHz, DMSO-*d*_6_) δ 9.77–9.26 (m, 2H), 8.06 (d, *J* = 8.6 Hz, 2H), 7.98 (d, *J* = 3.9 Hz, 1H), 7.95 (d, *J* = 3.9 Hz, 1H), 7.20 (d, *J* = 8.7 Hz, 2H), 5.28 (s, 1H), 3.51 (s, 1H), 2.23 (dt, *J* = 61.8, 31.3 Hz, 2H); ^13^C NMR (75 MHz, DMSO-*d*_6_) δ 168.43, 166.14, 159.70, 153.41, 140.32, 129.56, 119.57, 118.68, 116.76, 114.26, 76.26, 50.15, 43.83, 30.93. HRMS (ESI), *m*/*z* calcd C_16_H_15_N_4_O_5_ [M + H^+^] 343.1042, found 343.1044.

5-(5-nitro-2-furyl)-3-[2-(pyrrolidin-3-yloxy)phenyl]-1,2,4-oxadiazole hydrochloride (**2f**)

Yield 0.8 g (53%), white solid, m.p. 158–158.5 °С. ^1^H NMR (300 MHz, DMSO-*d*_6_) δ 9.86–8.84 (m, 2H), 8.04–7.97 (m, 2H), 7.96 (d, *J* = 4.0 Hz, 1H), 7.63 (t, *J* = 7.1 Hz, 1H), 7.37 (d, *J* = 8.3 Hz, 1H), 7.24 (t, *J* = 7.6 Hz, 1H), 5.36 (s, 1H), 3.52 (s, 1H), 3.46–3.37 (m, 3H), 2.30–2.11 (m, 2H); ^13^C NMR (75 MHz, DMSO-*d*_6_) δ 171.66, 169.87, 168.96, 167.40, 167.23, 155.38, 140.40, 133.65, 131.54, 122.37, 119.46, 116.22, 115.86, 114.29, 77.28, 50.13, 44.16, 31.35. HRMS (ESI), *m*/*z* calcd C_16_H_15_N_4_O_5_ [M + H^+^] 343.1042, found 343.1042.

4-(2-methoxyethyl)-4-[5-(5-nitro-2-furyl)-1,2,4-oxadiazol-3-yl]piperidine hydrochloride (**2g**)

Yield 0.67 g (47%), white solid, m.p. 165–165.5 °С. ^1^H NMR (300 MHz, DMSO-*d*_6_) δ 9.16–8.57 (m, 2H), 7.96 (d, *J* = 3.9 Hz, 1H), 7.90 (d, *J* = 3.8 Hz, 1H), 3.25 (t, *J* = 6.2 Hz, 3H), 3.09 (s, 3H), 2.89 (s, 2H), 2.36 (d, *J* = 14.8 Hz, 2H), 2.06–1.84 (m, 4H); ^13^C NMR (75 MHz, DMSO-*d*_6_) δ 173.70, 166.15, 153.33, 140.46, 119.39, 114.22, 67.84, 58.29, 36.56, 30.69. HRMS (ESI), *m*/*z* calcd C_14_H_19_N_4_O_5_ [M + H^+^] 323.1355, found 323.1352.

3-azetidin-3-yl-5-(5-nitro-2-furyl)-1,2,4-oxadiazole hydrochloride (**2h**)

Yield 0.62 g (57%), white solid, m.p. 173.5–174 °С. ^1^H NMR (300 MHz, DMSO-*d*_6_) δ 10.11–8.95 (m, 2H), 7.98 (d, *J* = 3.9 Hz, 1H), 7.94 (d, *J* = 4.0 Hz, 1H), 4.47–4.09 (m, 5H); ^13^C NMR (75 MHz, DMSO-*d*_6_) δ 170.19, 166.45, 153.51, 139.96, 119.83, 114.21, 48.71, 34.43, 27.82. HRMS (ESI), *m*/*z* calcd C_9_H_9_N_4_O_4_ [M + H^+^] 237.0623, found 237.0623.

2-[5-(5-nitro-2-furyl)-1,2,4-oxadiazol-3-yl]piperidine hydrochloride (**2i**)

Yield 0.52 g (43%), white solid, m.p. 160–160.5 °С. ^1^H NMR (300 MHz, DMSO-*d*_6_) δ 10.51–9.08 (m, 2H), 7.98 (q, *J* = 4.0 Hz, 2H), 4.81 (d, *J* = 9.0 Hz, 1H), 3.10 (s, 1H), 2.21 (d, *J* = 10.7 Hz, 1H), 1.98–1.53 (m, 6H); ^13^C NMR (75 MHz, DMSO-*d*_6_) δ 168.67, 166.64, 153.61, 139.62, 120.27, 114.18, 50.79, 44.44, 27.52, 21.59, 21.25. HRMS (ESI), *m*/*z* calcd C_11_H_13_N_4_O_4_ [M + H^+^] 265.0936, found 265.0935.

4-methyl-4-[5-(5-nitro-2-furyl)-1,2,4-oxadiazol-3-yl]piperidine hydrochloride (**2j**)

Yield 0.55 g (44%), white solid, m.p. 193–193.5 °С. ^1^H NMR (300 MHz, DMSO-*d*_6_) δ 9.05 (s, 2H), 7.96 (d, *J* = 4.0 Hz, 1H), 7.90 (d, *J* = 4.0 Hz, 1H), 3.21 (s, 2H), 2.95 (s, 2H), 2.32 (d, *J* = 14.3 Hz, 2H), 1.94 (t, *J* = 12.2 Hz, 2H), 1.38 (s, *J* = 2.7 Hz, 3H); ^13^C NMR (75 MHz, DMSO-*d*_6_) δ 175.31, 166.19, 153.35, 140.40, 119.43, 114.22, 66.75, 33.99, 31.90, 27.14. HRMS (ESI), *m*/*z* calcd C_12_H_15_N_4_O_4_ [M + H^+^] 279.1093, found 279.1096.

5-(5-nitro-2-furyl)-3-(pyrrolidin-3-ylmethyl)-1,2,4-oxadiazole hydrochloride (**2k**)

Yield 0.79 g (66%), white solid, m.p. 166–166.5 °С. ^1^H NMR (300 MHz, DMSO-*d*_6_) δ 9.62–8.78 (m, 2H), 7.96 (d, *J* = 4.0 Hz, 1H), 7.89 (d, *J* = 4.0 Hz, 1H), 3.31–3.19 (m, 2H), 3.20–3.08 (m, 1H), 3.02 (d, *J* = 7.1 Hz, 2H), 2.98–2.83 (m, 1H), 2.80–2.61 (m, 1H), 2.20–2.01 (m, 1H), 1.67 (tt, *J* = 17.1, 8.6 Hz, 1H); ^13^C NMR (75 MHz, DMSO-*d*_6_) δ 170.08, 165.91, 140.31, 119.41, 114.21, 49.25, 44.58, 35.71, 29.89, 28.23. HRMS (ESI), *m*/*z* calcd C_11_H_13_N_4_O_4_ [M + H^+^] 265.0936, found 265.0936.

5-(5-nitro-2-furyl)-3-pyrrolidin-3-yl-1,2,4-oxadiazole hydrochloride (**2l**)

Yield 0.6 g (53%), white solid, m.p. 209–209.5 °С. ^1^H NMR (300 MHz, DMSO-*d*_6_) δ 9.52 (s, 2H), 7.97 (d, *J* = 4.0 Hz, 1H), 7.92 (d, *J* = 3.9 Hz, 1H), 3.87 (p, *J* = 7.7 Hz, 1H), 3.66 (s, 1H), 2.46–2.35 (m, 1H), 2.25–2.12 (m, 1H); ^13^C NMR (75 MHz, CDCl_3_) δ 171.03, 166.28, 153.47, 140.07, 119.69, 114.19, 47.66, 44.97, 34.72, 29.36. HRMS (ESI), *m*/*z* calcd C_10_H_11_N_4_O_4_ [M + H^+^] 251.0780, found 251.0782.

3-(2-methoxyethyl)-4-[5-(5-nitro-2-furyl)-1,2,4-oxadiazol-3-yl]piperidine hydrochloride (**2m**)

Yield 0.81 g (57%), white solid, m.p. 168–168.5 *°*С. ^1^H NMR (300 MHz, DMSO-d_6_) δ 9.29 (s, 1H), 8.27 (s, 1H), 7.95 (q, *J* = 4.1 Hz, 2H), 3.69 (d, *J* = 12.5 Hz, 1H), 3.29–3.14 (m, 5H), 3.01–2.84 (m, 1H), 2.39–2.26 (m, 1H), 2.06–1.64 (m, 6H); ^13^C NMR (75 MHz, DMSO-d_6_) δ 172.58, 166.16, 140.20, 119.63, 114.21, 67.62, 58.30, 49.23, 43.38, 30.18, 19.00. HRMS (ESI), *m*/*z* calcd C_14_H_19_N_4_O_5_ [M + H^+^] 323.1355, found 323.1356.

### 3.2. Biological Activity Evaluation

Testing was conducted against the following microorganisms: *Enterococcus faecalis* (ATCC 29812), *Staphylococcus aureus* (ATCC 25912), *Klebsiella pneumoniae* (ATCC 19882), *Acinetobacter baumannii* (948^®^, a patient-derived strain from the Pasteur Institute’s own collection), *Pseudomonas aeruginosa* (ATCC 27853), and *Enterobacter cloacae* (ATCC 13047) for compounds **2a**–**m** and ciprofloxacin (employed as a positive control) using the Kirby–Bauer disk diffusion test under the Standard Operating Procedure of the European Committee on Antimicrobial Susceptibility Testing (EUCAST) [35]. Paper disks bearing 5 mg of the tested compounds were used. Solutions of tested compounds in DMSO (1 mg/10 mL) were prepared and diluted to a total volume of 1 mL with deionized water. Aliquots of the resulting solutions (5 µL each) were added to a Petri dish containing Muller–Hilton agar that was inoculated with a bacterial suspension (McFarland OD 1/4 0.5). After the compound solution had dried off, the Petri dish was incubated at 37 °C for 18 h. The bacterial growth inhibition zone diameter around the disc with ciprofloxacin or the compounds’ dried solution circular spot indicated the general susceptibility to a drug being assessed. Thereupon, minimum inhibitory concentrations (MIC, µg/mL) were determined using serial broth dilutions [36]. All measurements were done in triplicate. All compounds **2a**–**m** were tested against the *Mycobacterium tuberculosis* H37Rv drug-sensitive strain relative to isoniazid that was employed as a positive control. The testing was performed as described previously [37]. All the measurements were done in triplicate.

### 3.3. In Silico Studies

#### 3.3.1. Target Selection

For each microorganism, a different protein structure was selected (unless they were 100% homologous). The table provided in the Supporting Information (Table S1) summarizes all used protein structures present in the RCSB Protein Data Bank. Most of the proteins were represented as models generated with AlphaFold [38]. In terms of pLDDT, all structures were of acceptable quality when compared to the experimental models (pLDDT > 90 or >70). The considered protein structures underwent a structure alignment procedure to identify active sites for subsequent molecular docking of the studied compounds and parameterization of their affinity to the above targets.

#### 3.3.2. Protein and Ligand Structure Preparation

All proteins (enzymes) were preprocessed before calculations using the protein prep wizard tool from the Schrodinger suite. During preprocessing, errors such as missing amino acid sidechains, incorrect protonation states, missing hydrogens, incorrect bond orders, angles, etc., were fixed. The ligand geometry was generated by the LigPrep module. All molecular modifications were carried out in the OPLS4 force field [39]. Schrödinger Suite 2022-4 was used for calculations.

#### 3.3.3. Induced-Fit Docking of Molecules

IFD docking, a modeling technique that allows the estimation of ligand-induced changes in receptor structure, is a variation in molecular docking that considers the possible mobility of a receptor upon binding to a small molecule within a given radius (in this case, 5 angstroms around the ligand) in a limited way.

Docking grid box calculated based on reference ligand position and size (grid placement on complex ligand centroid, maximum grid side size is 12 Å). In the case of ligand absence docking, the grid box is calculated by residues involved in potential interactions. Twenty poses were generated for each ligand. The best-fitting pose was selected by comparison with the position of the nitrofuran moiety of the reference ligand in the active site of the protein (if available in the PDB files). The clustering of its binding poses was also an important parameter showing ligand binding quality. Additionally, the observed cluster must replicate the pharmacophore characteristics of the reference ligands. This value is shown as stars in parentheses. Three stars: more than 60% clustered docking solutions with RMSD less than 1.5 Å; two stars: 40–60% clustered docking solutions with RMSD less than 3 Å; one star: less than 40% or solutions with no clustering.

## 4. Conclusions

A series of 13 new 3-substituted 5-(5-nitro-2-furyl)-1,2,4-oxadiazoles was synthesized in two steps. Most of the compounds showed the same activity as ciprofloxacin against *S. aureus* and some Gram-positive ESKAPE pathogens. Compounds **2c** and **2h** were definitely the leads. Small structural differences in the molecular periphery lead to high selectivity of compounds against individual pathogens. The significance of these differences was confirmed by molecular docking. Possible biological targets were determined by calculations. Compounds **2e** and **2d** showed the best anti-tuberculosis activity, while **2d** did not show activity against ESKAPE pathogens. The calculations indicated a mechanism of action that is unconventional for nitrofurans, and the better activity of **2e** may be explained by a synergistic effect due to a possible multi-target interaction. We found that for the studied compounds, the ligand–protein contacts within their most probable targets can have a big impact on how active they are. In this case, the introduction of a small-sized substituent, as in **2d**, led to a loss of activity due to a significant loss of orienting lipophilic contacts, which provided specific recognition of the binding site. On the other hand, in the case of **2g**, the substituent at the oxadiazole part of the scaffold had a tendency to form too many lipophilic contacts, which caused significant changes in the way of binding. This can even shift the nitro group from the active center of the protein (in the case of nitroreductases). Regarding the designed compounds, it’s really important to strike a balance between the volume and lipophilicity properties of the introduced substituent.

## Figures and Tables

**Table 1:** Antibacterial activity (disk diffusion method inhibition zone (IZ, mm) of compounds **2a**–**m** and nitrofurantoin (NFt) and furazidin (FZ) as positive control against the ESKAPE panel of pathogens. The compounds with broad activity spectra are highlighted by green. The most vulnerable pathogen is highlighted by pink.

ESKAPE Panel	Compound 2													NFt	FZ
	a	b	c	d	e	f	g	h	i	j	k	l	m		
*E. faecium*	0	0	13 ± 1.6	0	7 ± 1.6	0	0	0	0	0	0	0	0	15 ± 1.6	13 ± 1.0
*S. aureus*	21 ± 1.6	17 ± 1.0	21 ± 1.6	0	27 ± 0.4	21 ± 1.0	0	21 ± 1.6	21	25 ± 1.0	0	15 ± 0.3	17 ± 1.2	21 ± 0.3	23 ± 1.6
*K. pneumoniae*	0	0	25 ± 1.0	0	9 ± 1.6	0	0	17 ± 0.3	0	0	0	0	0	12 ± 1.0	14 ± 1.6
*A. baumannii*	0	0	0	0	0	0	0	23 ± 1.0	0	0	13 ± 0.1	0	0	0	0
*P. aeruginosa*	15	0	0	0	0	0	0	0	0	0	0	0	0	0	0
*E. cloacae*	0	13	21 ± 1.6	0	0	0	0	19 ± 0.5	0	0	0	0	0	24 ± 0.4	21 ± 1.0

**Table 2:** Minimal inhibitory concentration (MIC, µg/mL) of tested compounds and ciprofloxacin (positive control) against the ESKAPE panel of pathogens and MTB; nt—not tested. The MIC values are mean from three different assays (errors were in the range of ±5–10% of the reported values). The MIC values less than positive control are highlighted by green.

Compounds	Pathogens						
	*E. faecium*	*S. aureus*	*K. pneumoniae*	*A. baumannii*	*P. aeruginosa*	*E. cloacae*	*M. tuberculosis*
**2c**	4 ± 0.1	6 ± 0.6	0.5 ± 0.1	32 ± 0.1	4 ± 0.03	0.25 ± 0.01	100 ± 10.0
**2h**	1 ± 0.03	0.8 ± 0.01	1.75 ± 0.5	0.4 ± 0.1	9 ± 0.6	3.5 ± 0.1	50 ± 3.0
**2e**	nt	nt	nt	nt	nt	nt	6.2 ± 0.4
**2d**	nt	nt	nt	nt	nt	nt	25 ± 1.8
**2f**,**i**,**k**	nt	nt	nt	nt	nt	nt	50 ± 3.1
**2a**,**b**,**g**,**j**,**l**,**m**	nt	nt	nt	nt	nt	nt	≥100
nitrofurantoin	8 ± 0.1	4 ± 0.1	64 ± 3.0	nt	nt	4 ± 0.1	nt
furazidine	32 ± 0.1	8 ± 0.1	32 ± 3.0	nt	nt	2 ± 0.1	nt
ciprofloxacin	1.25 ± 0.1	1.25 ± 0.1	0.6 ± 0.1	2.5 ± 0.1	0.6 ± 0.1	3 ± 0.1	0.5–0.25 [24]

**Table 3:** Results of molecular docking of the investigated compounds into the active sites of the most likely targets associated with antibiotics of the nitrofuran class, stars indicate the quality of binding pose, where: ***—good, **—fair, *—low. Coloring in «Compounds» column: red—inactive compounds at all targets/species, dark green and faded green—active/moderately active compounds. Coloring in «GlideScore» columns: green—correlated with experiment, yellow—fair correlation, brown—correlation with negative control. n/a—docking pose can’t be obtained.

Compounds	GlideScore (kcal/mol) and Binding Quality (Stars)												
	AzoR			NfsA			NfsB			Pgk			Ddn
	*P. aeruginosa*	*S. aureus*	*A. baumannii*	*P. aeruginosa*	*S. aureus*	*A. baumannii*	*P. aeruginosa*	*S. aureus*	*A. baumannii*	*P. aeruginosa*	*S. aureus*	*A. baumannii*	*M. tuberculosis*
**2a**	−7.51 (***)	−6.81 (**)	−8.44 (***)	−5.47 (***)	−7.18 (***)	−6.15 (***)	−5.16 (**)	−6.03 (***)	−5.41 (*)	−6.77 (***)	−6.50 (*)	−6.88 (***)	−6.70 (***)
**2c**	−6.95 (***)	−7.53 (***)	−7.95 (***)	−5.14 (***)	−5.83 (***)	−6.15 (***)	−5.46 (*)	−5.75 (**)	−5.77 (**)	−8.24 (***)	−5.52 (*)	−8.40 (***)	n/a
**2d**	−6.58 (*)	−6.81 (*)	−6.69 (*)	−6.25 (*)	−5.89 (*)	−6.09 (*)	−5.71 (*)	−7.20 (*)	−5.79 (*)	−6.98 (*)	−5.99 (*)	−6.18 (***)	n/a
**2e**	−8.89 (***)	−8.28 (**)	−9.23 (*)	−4.77 (*)	−6.02 (*)	−5.40 (***)	−7.04 (*)	−6.63 (**)	−5.35 (*)	−8.38 (***)	−6.71 (*)	−7.57 (**)	−7.74 (***)
**2f**	−6.51 (*)	−7.87 (***)	−8.24 (***)	−4.79 (*)	−6.60 (*)	−6.78 (***)	−6.44 (**)	−7.61 (***)	−5.24 (*)	−8.55 (***)	−7.32 (*)	−8.65 (***)	−6.01 (*)
**2g**	−7.59 (*)	−8.64 (*)	−7.24 (*)	−5.63 (*)	−5.82 (*)	−7.05 (*)	−5.88 (*)	−6.16 (*)	−5.83 (*)	−7.00 (**)	−6.91 (*)	−6.45 (***)	n/a
**2h**	−7.46 (***)	−7.11 (***)	−7.48 (***)	−5.13 (***)	−7.19 (***)	−6.94 (***)	−4.60 (**)	−5.98 (***)	−5.07 (*)	−6.60 (***)	−7.16 (*)	−7.02 (***)	n/a
**2i**	−6.09 (***)	−7.01 (***)	−7.83 (***)	−4.77 (*)	−7.03 (***)	−6.35 (***)	−7.16 (**)	−7.43 (***)	−7.20 (*)	−6.98 (***)	−5.79 (*)	−8.13 (***)	−5.83 (***)
**2j**	−6.37 (***)	−7.40 (**)	−7.89 (***)	−4.57 (*)	−6.62 (**)	−7.06 (**)	−5.63 (**)	−6.21 (***)	−6.11 (*)	−7.06 (***)	−6.28 (*)	−6.46 (***)	−6.54 (***)
**2k**	−7.25 (***)	−7.55 (**)	−6.14 (***)	−5.32 (*)	−5.90 (*)	−4.66 (*)	−5.86 (*)	−6.67 (***)	−7.24 (*)	−7.65 (***)	−6.98 (*)	−8.14 (***)	−5.80 (***)

**Table 4:** Results of molecular docking of tested compounds with proteins considered as promising targets for tuberculosis therapy. Stars indicate the quality of binding pose, where: ***—good, **—fair, *—low. Active compounds highlighted by green, most active highlighted by dark green. N/A—binding pose can’t be calculated.

	GlideScore (kcal/mol)/Quality of Binding Pose (Stars)							
Protein	2a	2c	2d	2e	2g	2h	2k	Control
EthR	−8.67 (*)	−7.80 (**)	−7.47 (**)	−10.32 (***)	−7.66 (*)	−8.05 (**)	−8.29 (**)	−10.80 (***)
TBNAT	−5.86 (*)	−4.91 (*)	−5.22 (**)	−5.85 (**)	−6.39 (*)	−4.70 (**)	−4.88 (*)	N/A
InhA	−7.23 (***)	−6.57 (***)	−5.57 (*)	−7.66 (***)	−6.23 (*)	−6.86 (***)	−7.20 (***)	−9.70 (***)

## Data Availability

Data are contained within the article and Supplementary Materials.

## Supplementary Materials

The following supporting information can be downloaded at: https://www.mdpi.com/article/10.3390/molecules29143364/s1, Copies of ^1^H and ^13^C NMR spectra; molecular modeling data and visualization. References [7,27,40,41] are cited in the Supplementary Materials.
