# Supplementary material for: Molecular Periphery Design Allows Control of the New Nitrofurans Antimicrobial Selectivity
Source: Molecules. 2024 Jul 17;29(14):3364. doi: 10.3390/molecules29143364 (PMC11279955; doi:10.3390/molecules29143364)

Supporting Information

Molecular Periphery Design Allows Control Of The New Nitrofurans Antimicrobial Selectivity

Lyubov Vinogradova [1], Alexey Lukin [1], Kristina Komarova [1], Maxim Zhuravlev [1], Artem Fadeev [1], Mikhail Chudinov [1,*], Elizaveta Rogacheva [2], Lyudmila Kraeva [2], Maxim Gureev [3], Yuri Porozov [4,5], Marine Dogonadze [6] and Tatiana Vinogradova [6]

1 Lomonosov Institute of Fine Chemical Technologies, MIREA—Russian Technological University, Moscow 119454, Russia
2 Pasteur Institute of Epidemiology and Microbiology, Saint Petersburg 197101, Russia
3 Institute of Cytology, Russian Academy of Sciences, Tikhoretsky Ave. 4, Saint Petersburg 194064, Russia
4 Laboratory of Angiopathology, The Institute of General Pathology and Pathophysiology, 8 Baltiyskaya Street, 125315 Moscow, Russia
5 Advitam Laboratory, Mihaila Shushkaloviħa 13, 11030 Belgrade, Serbia
6 Saint-Petersburg State Research Institute of Phthisiopulmonology of the Ministry of Healthcare of the Russian Federation, Saint Petersburg 191036, Russia
* Correspondence: chudinov@mirea.ru

NMR spectra of synthesized compounds

tert-butyl 4-[(hydroxyamino)(imino)methyl]piperidine-1-carboxylate (1a) 3

tert-butyl 3-[(hydroxyamino)(imino)methyl]piperidine-1-carboxylate (1b) 4

tert-butyl [3-(hydroxyamino)-3-iminopropyl]methylcarbamate (1c) 5

tert-butyl [2-(hydroxyamino)-2-iminoethyl]methylcarbamate (1d) 6

tert-butyl 3-{4-[(hydroxyamino)(imino)methyl]phenoxy}pyrrolidine-1-carboxylate (1e) 7

tert-butyl 3-{2-[(hydroxyamino)(imino)methyl]phenoxy}pyrrolidine-1-carboxylate (1f) 8

tert-butyl 4-[(hydroxyamino)(imino)methyl]-4-(2-methoxyethyl)piperidine-1-carboxylate (1g) 9

tert-butyl 3-[(hydroxyamino)(imino)methyl]azetidine-1-carboxylate (1h) 10

tert-butyl 2-[(hydroxyamino)(imino)methyl]piperidine-1-carboxylate (1i) 11

tert-butyl 4-[(hydroxyamino)(imino)methyl]-4-methylpiperidine-1-carboxylate (1j) 12

tert-butyl 3-[2-(hydroxyamino)-2-iminoethyl]pyrrolidine-1-carboxylate (1k) 13

tert-butyl 3-[(hydroxyamino)(imino)methyl]pyrrolidine-1-carboxylate (1l) 14

tert-butyl 3-[(hydroxyamino)(imino)methyl]-3-(2-methoxyethyl)piperidine-1-carboxylate (1m) 15

4-[5-(5-nitro-2-furyl)-1,2,4-oxadiazol-3-yl]piperidine hydrochloride (2a) 16

3-[5-(5-nitro-2-furyl)-1,2,4-oxadiazol-3-yl]piperidine hydrochloride (2b) 17

N-methyl-2-[5-(5-nitro-2-furyl)-1,2,4-oxadiazol-3-yl]ethanamine hydrochloride (2c) 18

N-methyl-1-[5-(5-nitro-2-furyl)-1,2,4-oxadiazol-3-yl]methanamine hydrochloride (2d) 19

5-(5-nitro-2-furyl)-3-[4-(pyrrolidin-3-yloxy)phenyl]-1,2,4-oxadiazole hydrochloride (2e) 20

5-(5-nitro-2-furyl)-3-[2-(pyrrolidin-3-yloxy)phenyl]-1,2,4-oxadiazole hydrochloride (2f) 21

4-(2-methoxyethyl)-4-[5-(5-nitro-2-furyl)-1,2,4-oxadiazol-3-yl]piperidine hydrochloride (2g) 22

3-azetidin-3-yl-5-(5-nitro-2-furyl)-1,2,4-oxadiazole hydrochloride (2h) 23

2-[5-(5-nitro-2-furyl)-1,2,4-oxadiazol-3-yl]piperidine hydrochloride (2i) 24

4-methyl-4-[5-(5-nitro-2-furyl)-1,2,4-oxadiazol-3-yl]piperidine hydrochloride (2j) 25

5-(5-nitro-2-furyl)-3-(pyrrolidin-3-ylmethyl)-1,2,4-oxadiazole hydrochloride (2k) 26

5-(5-nitro-2-furyl)-3-pyrrolidin-3-yl-1,2,4-oxadiazole hydrochloride (2l) 27

3-(2-methoxyethyl)-4-[5-(5-nitro-2-furyl)-1,2,4-oxadiazol-3-yl]piperidine hydrochloride (2m)........ 28

tert-butyl 4-[(hydroxyamino)(imino)methyl]piperidine-1-carboxylate (1a)

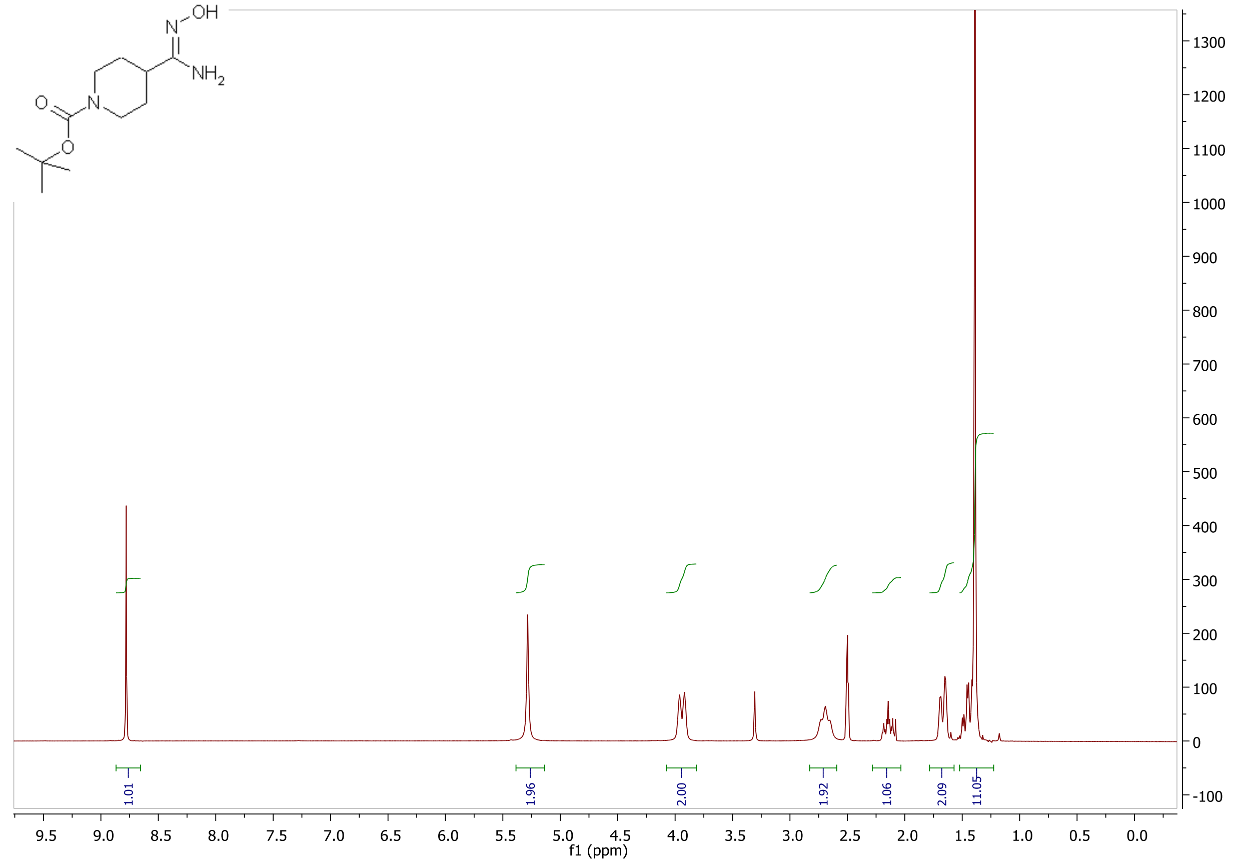

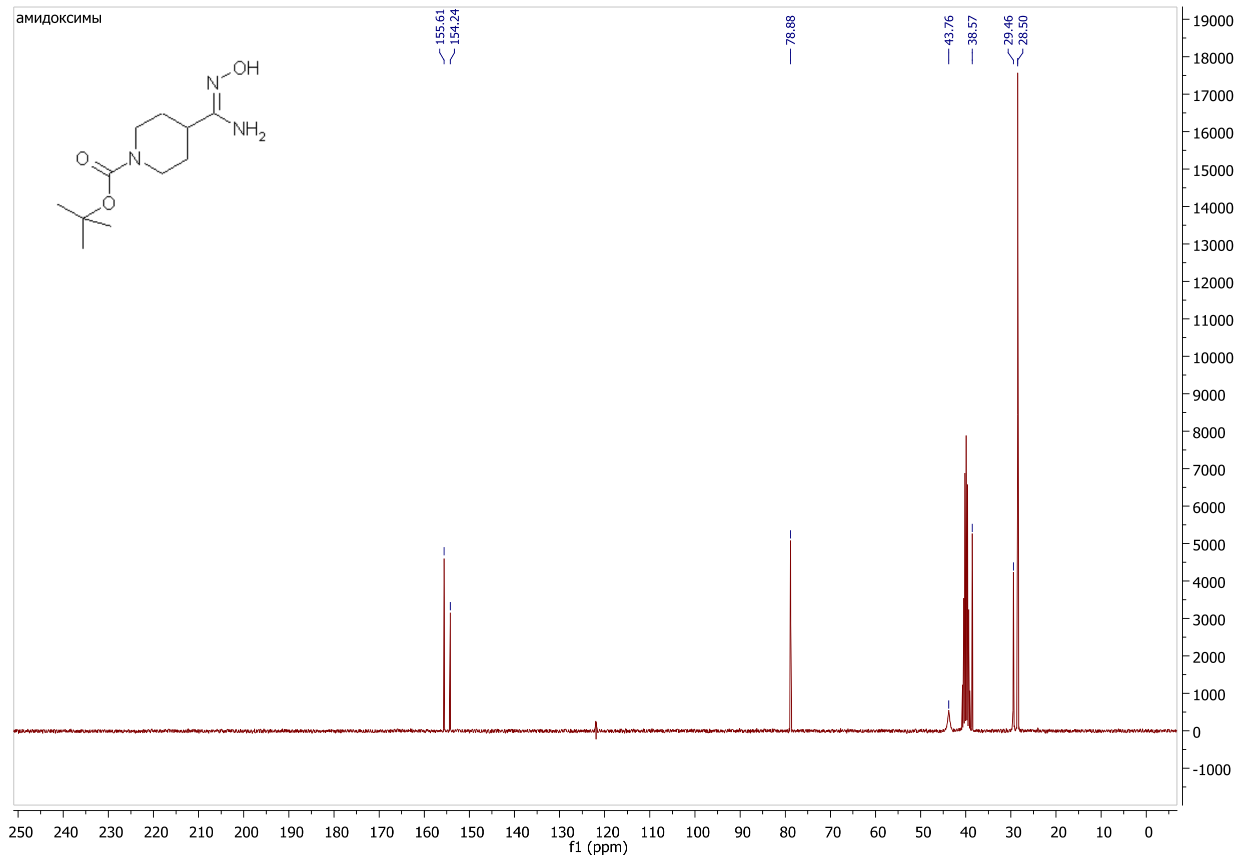

tert-butyl 3-[(hydroxyamino)(imino)methyl]piperidine-1-carboxylate (1b)

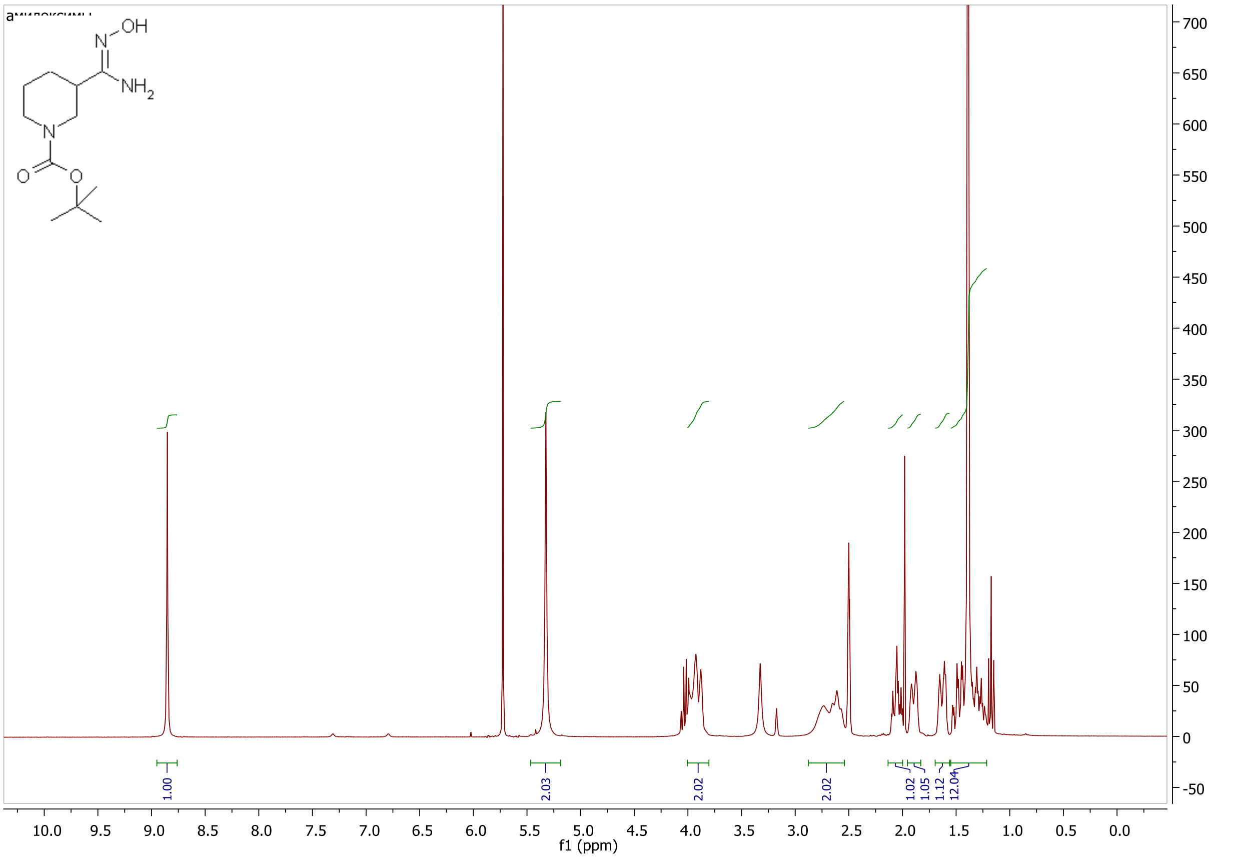

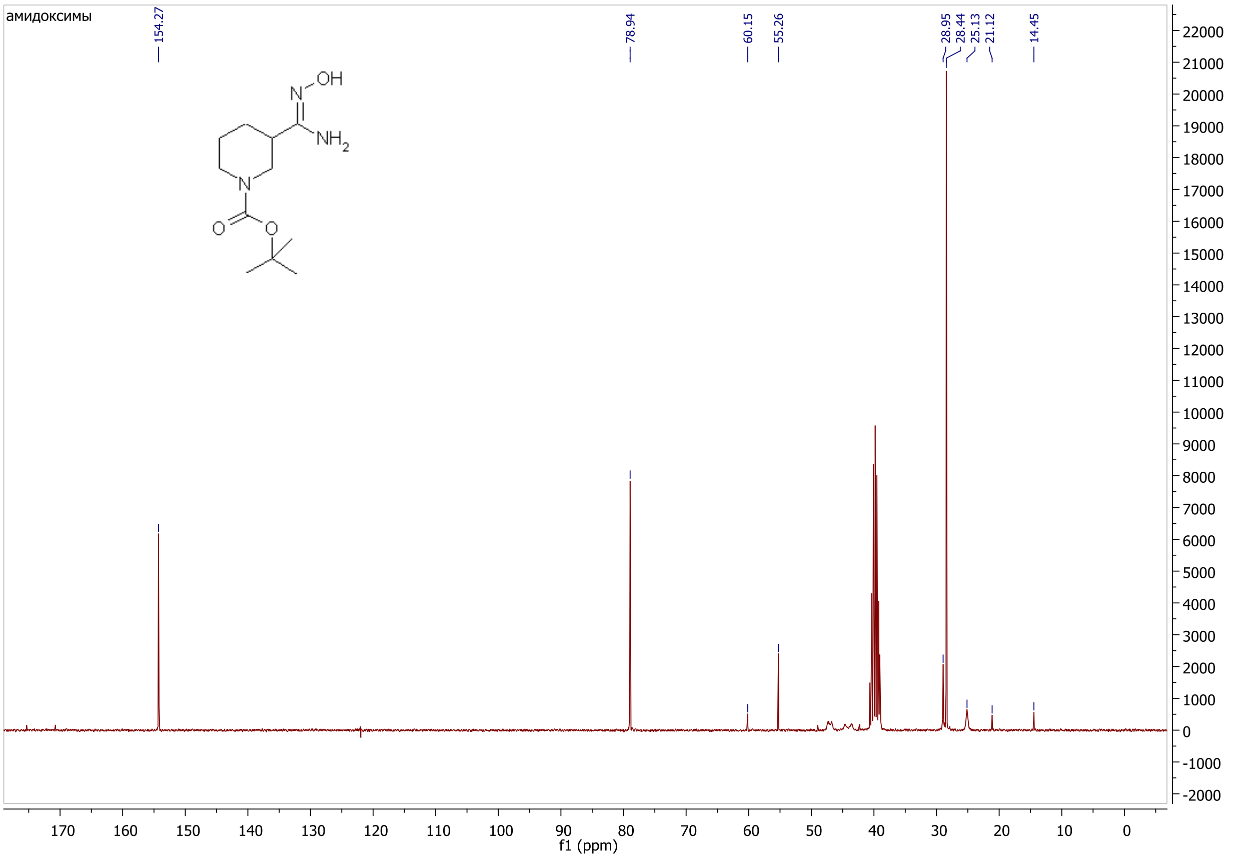

tert-butyl [3-(hydroxyamino)-3-iminopropyl]methylcarbamate (1c)

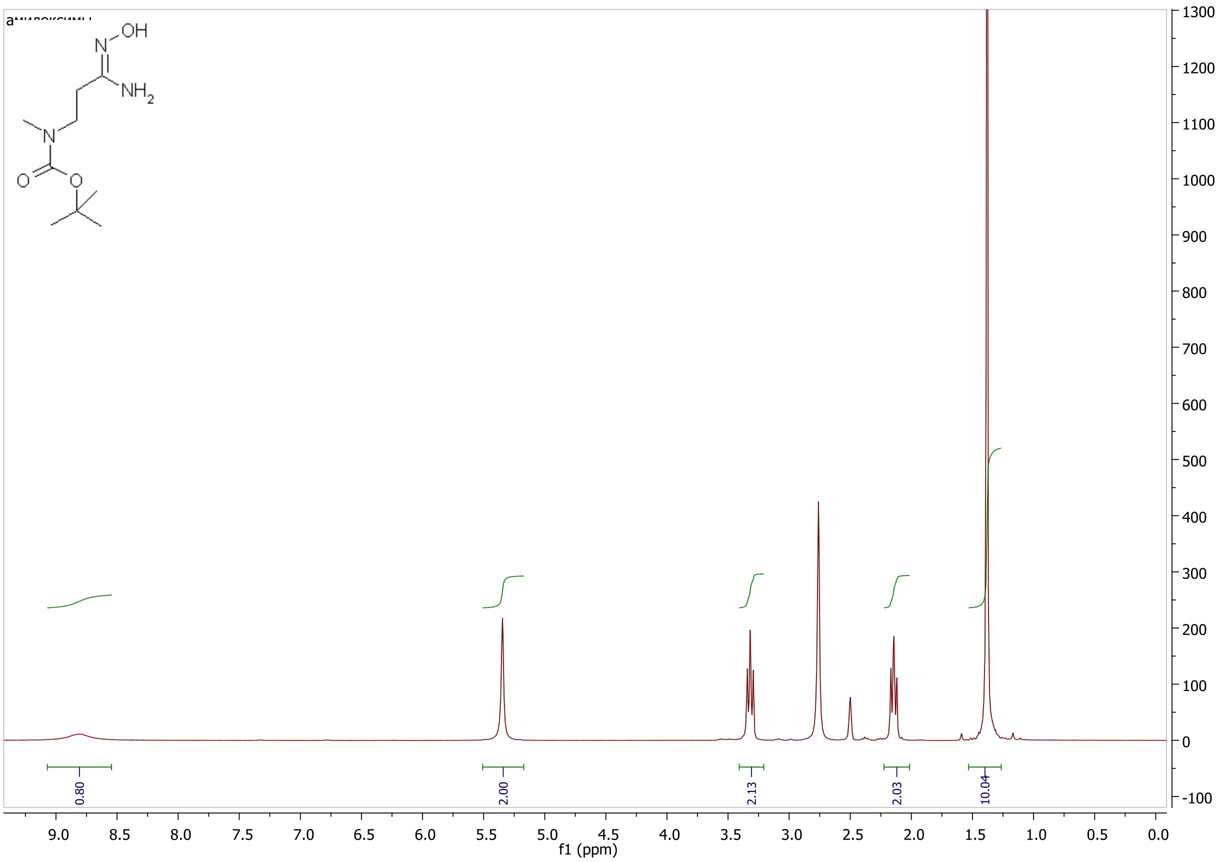

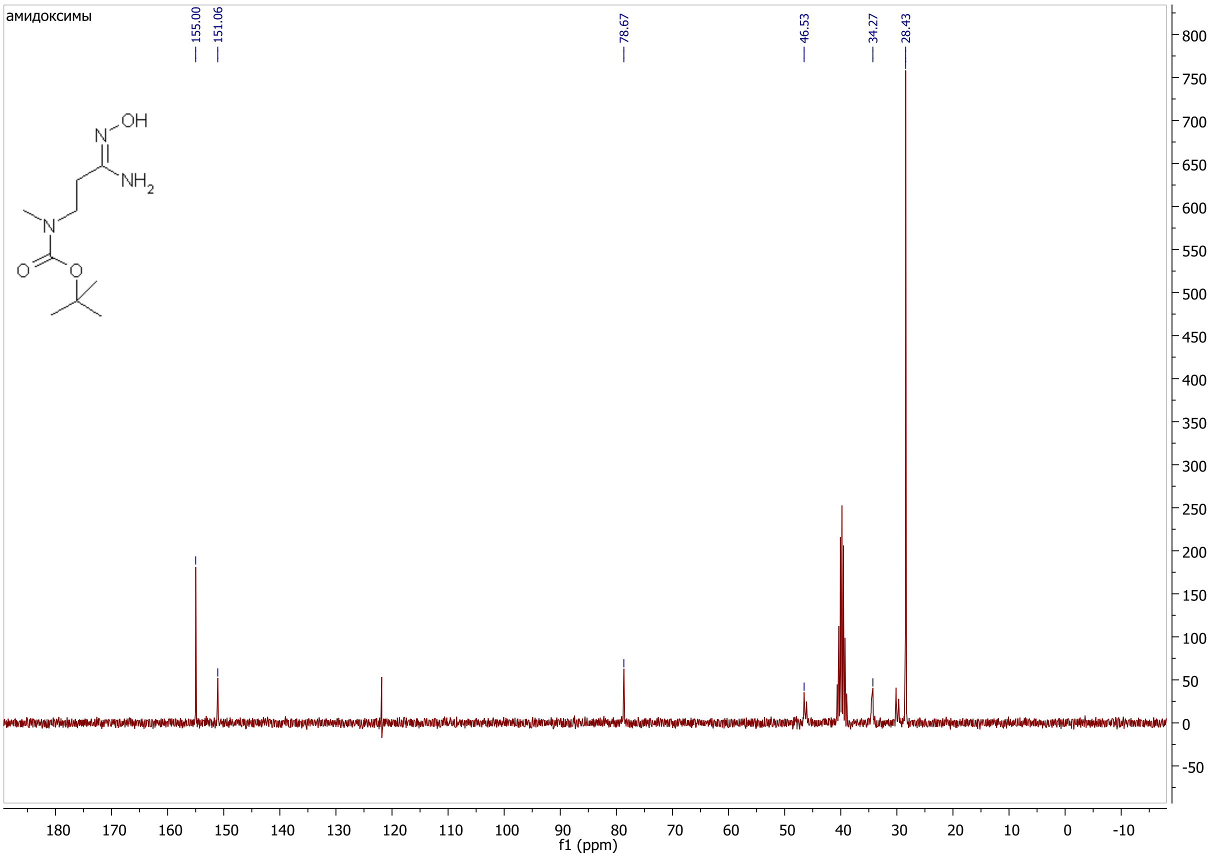

tert-butyl [2-(hydroxyamino)-2-iminoethyl]methylcarbamate (1d)

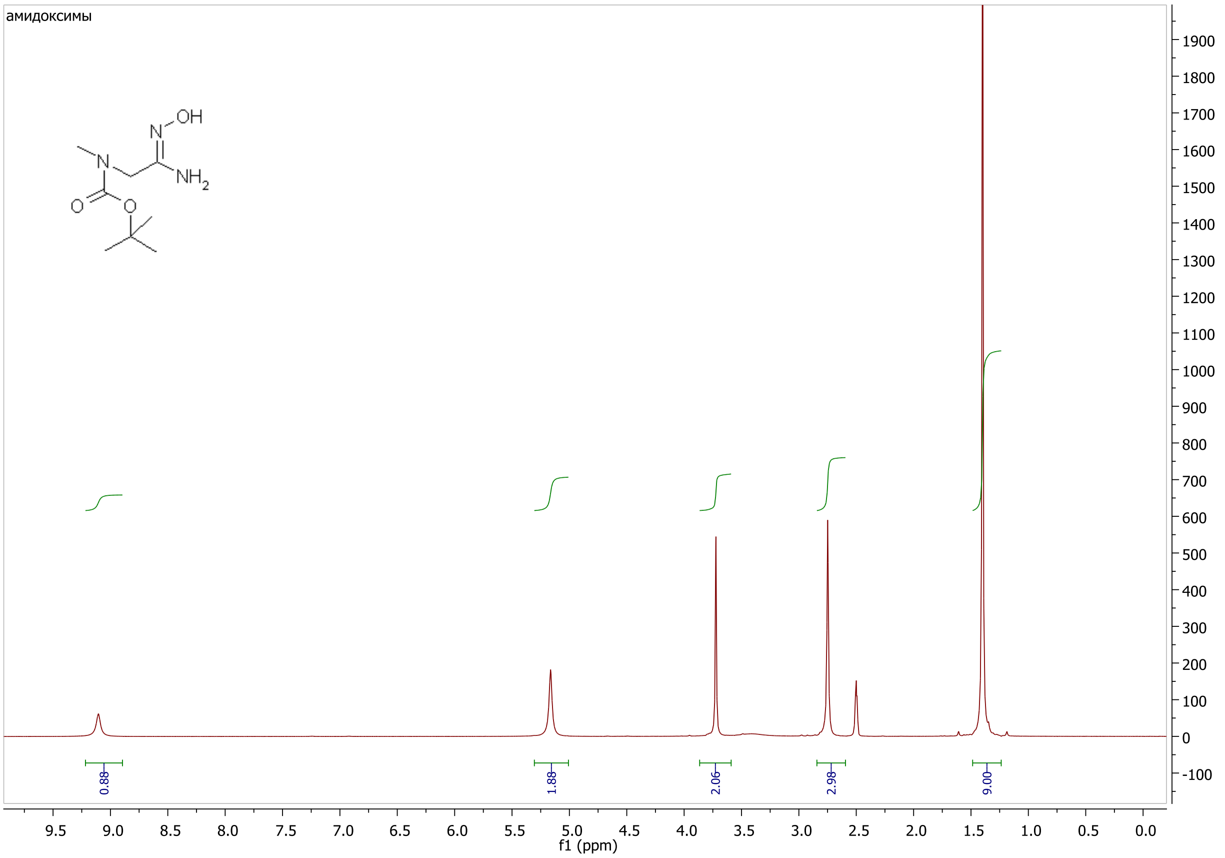

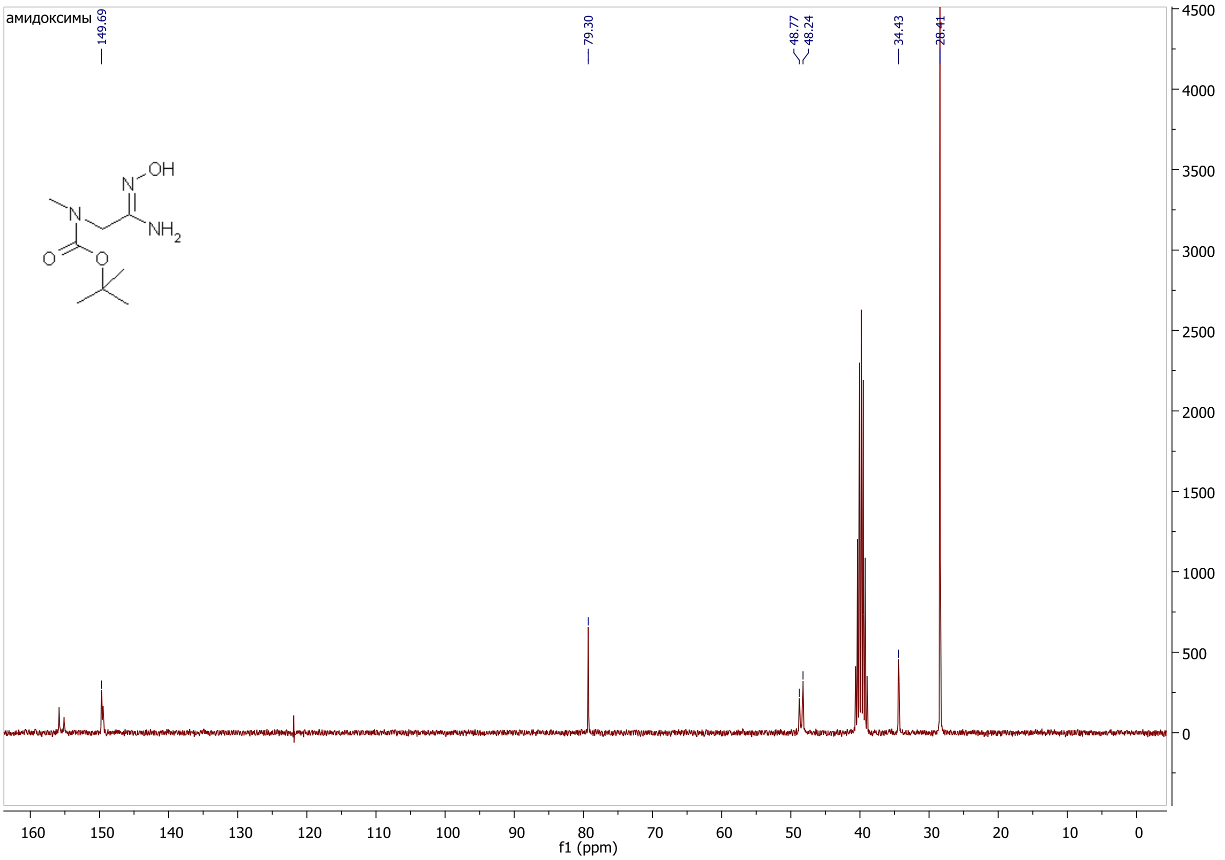

tert-butyl 3-{4-[(hydroxyamino)(imino)methyl]phenoxy}pyrrolidine-1-carboxylate (1e)

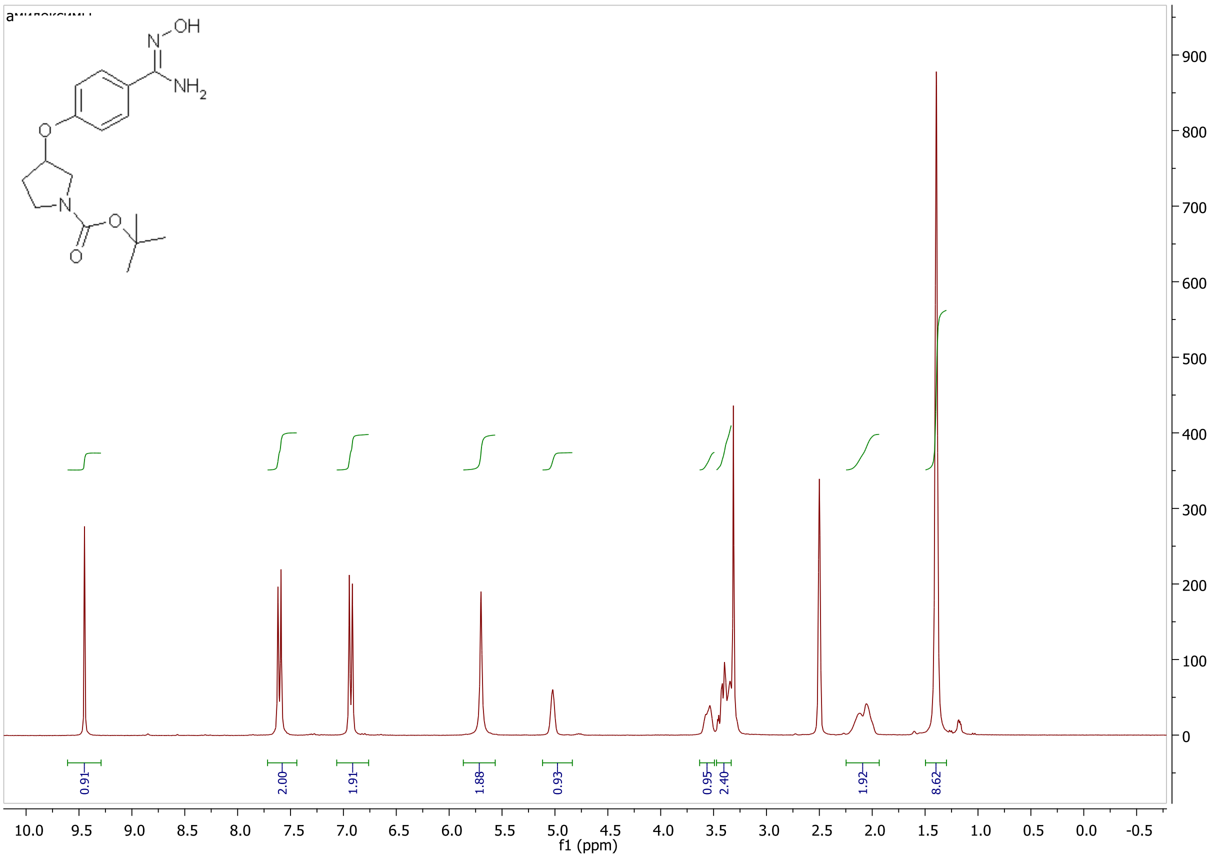

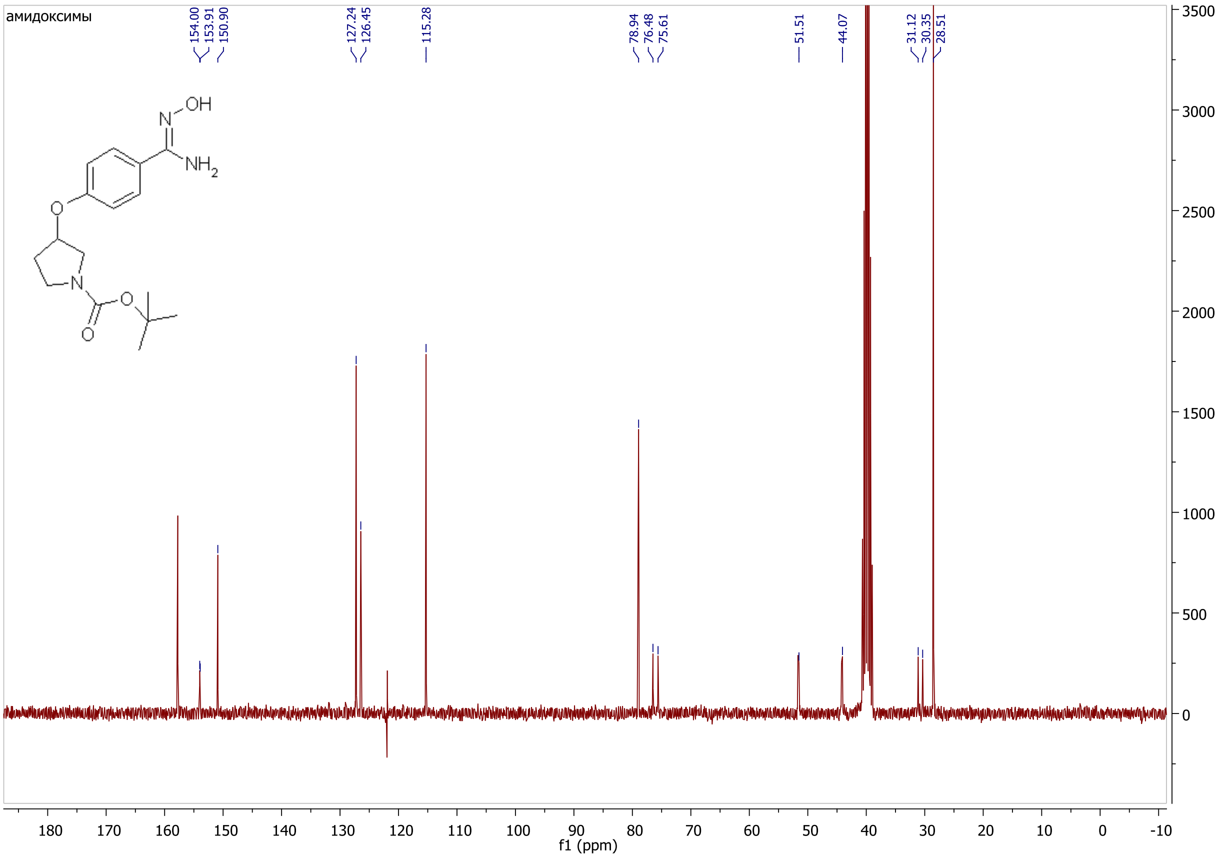

tert-butyl 3-{2-[(hydroxyamino)(imino)methyl]phenoxy}pyrrolidine-1-carboxylate (1f)

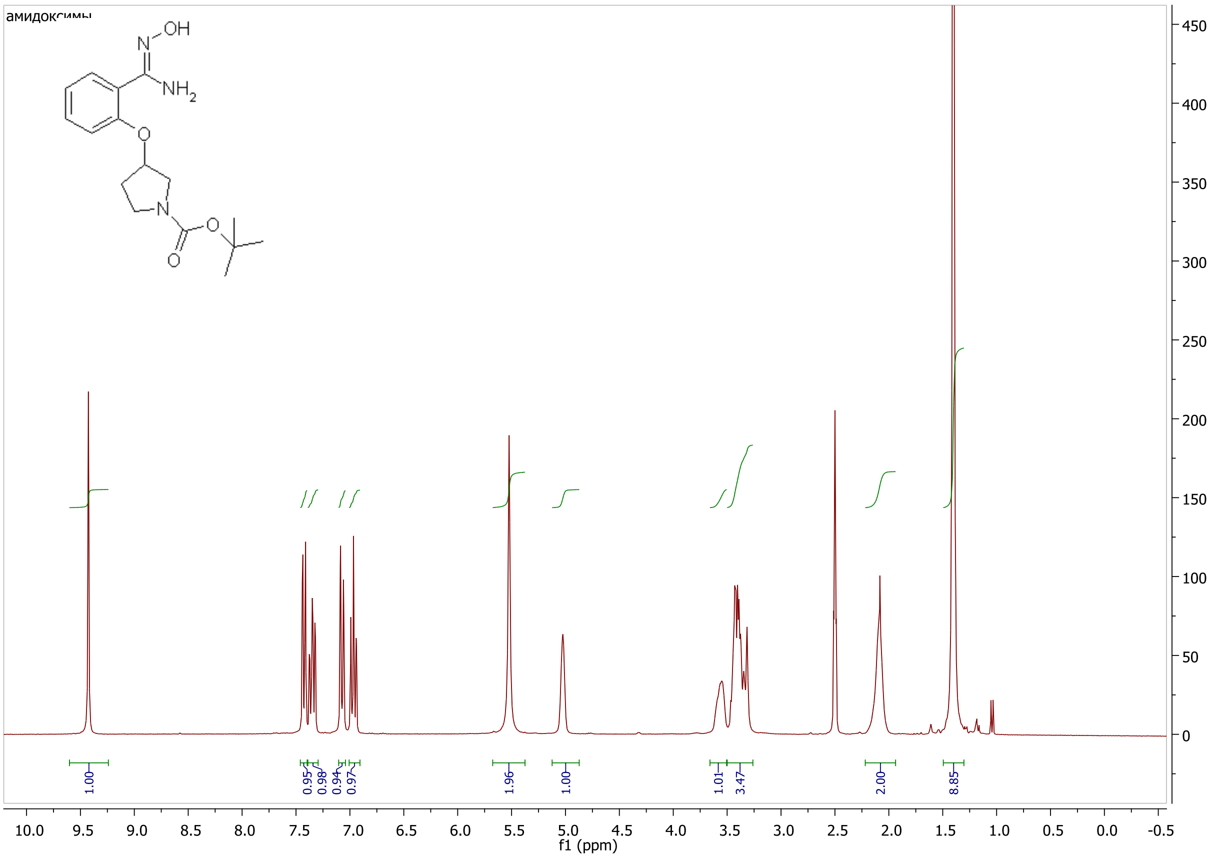

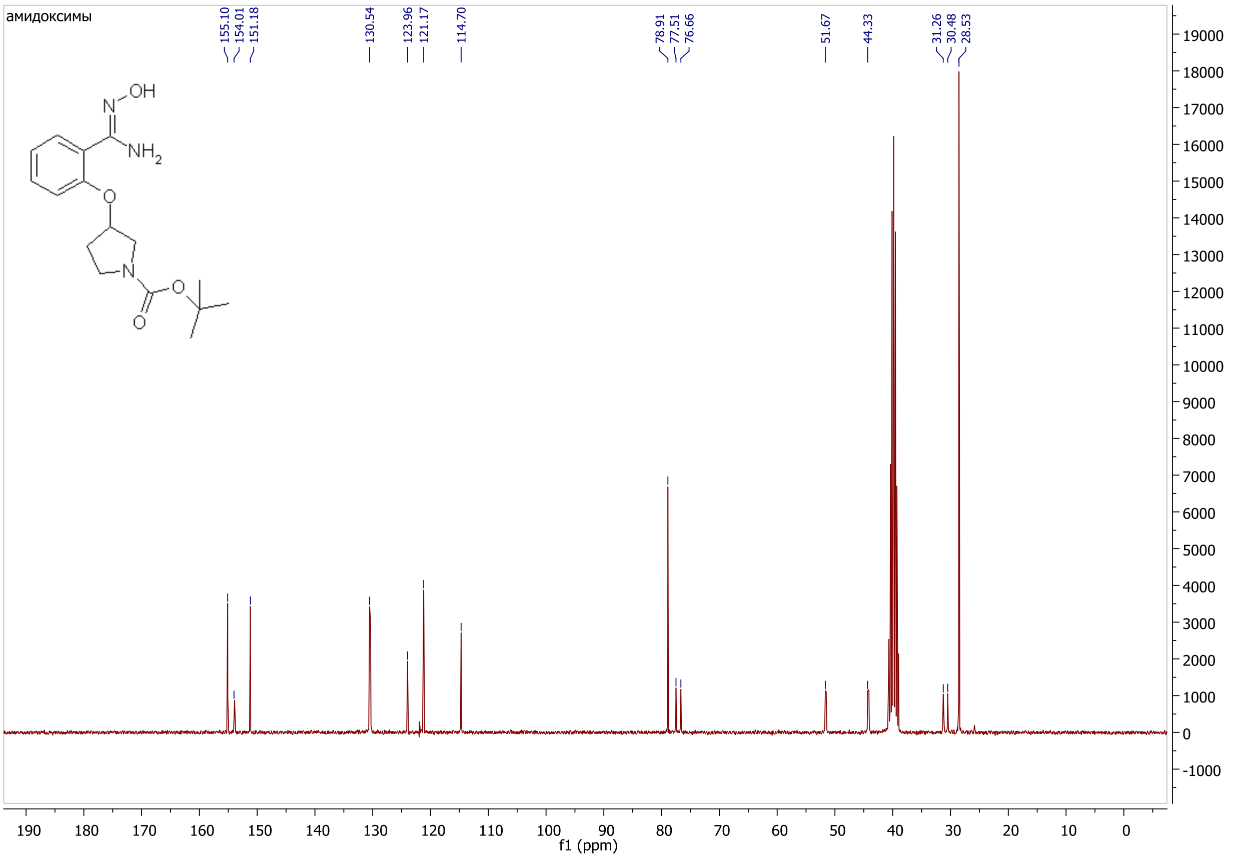

tert-butyl 4-[(hydroxyamino)(imino)methyl]-4-(2-methoxyethyl)piperidine-1-carboxylate (1g)

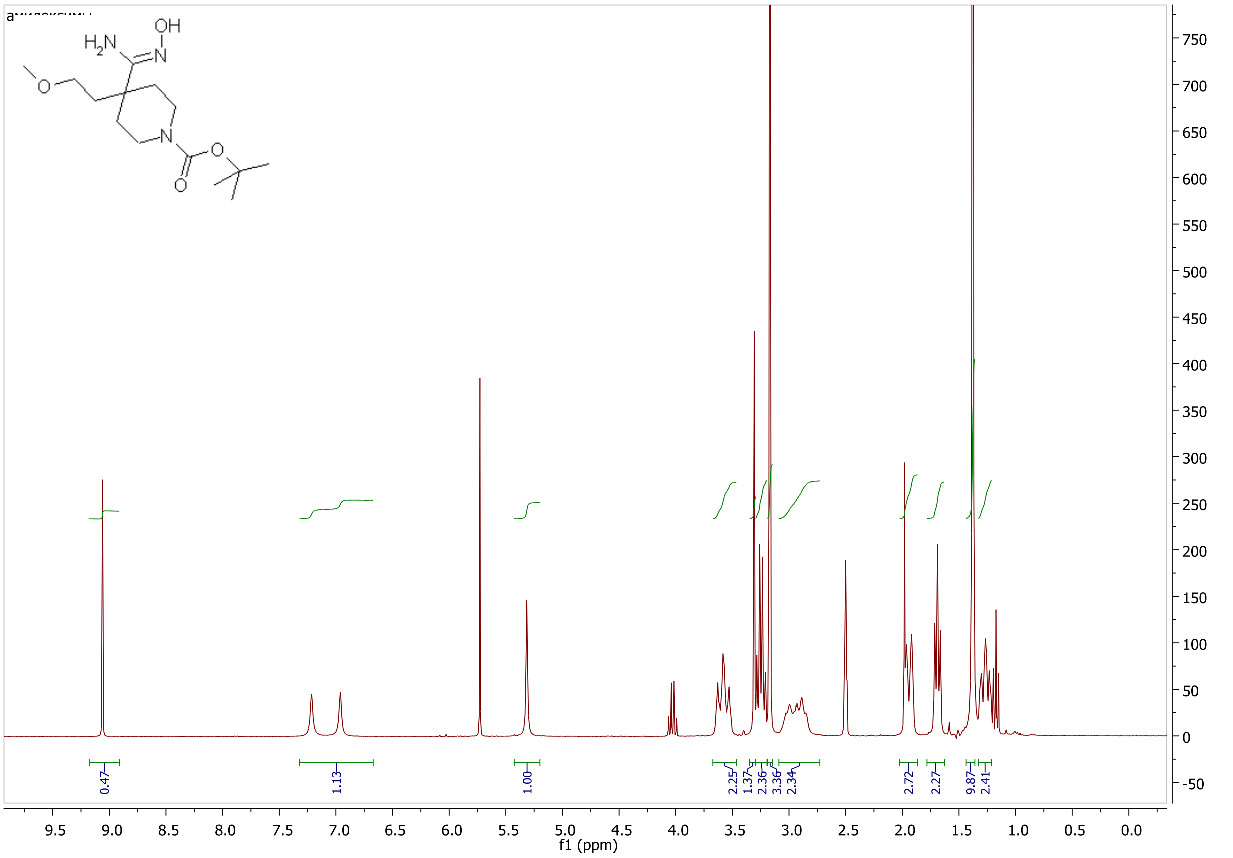

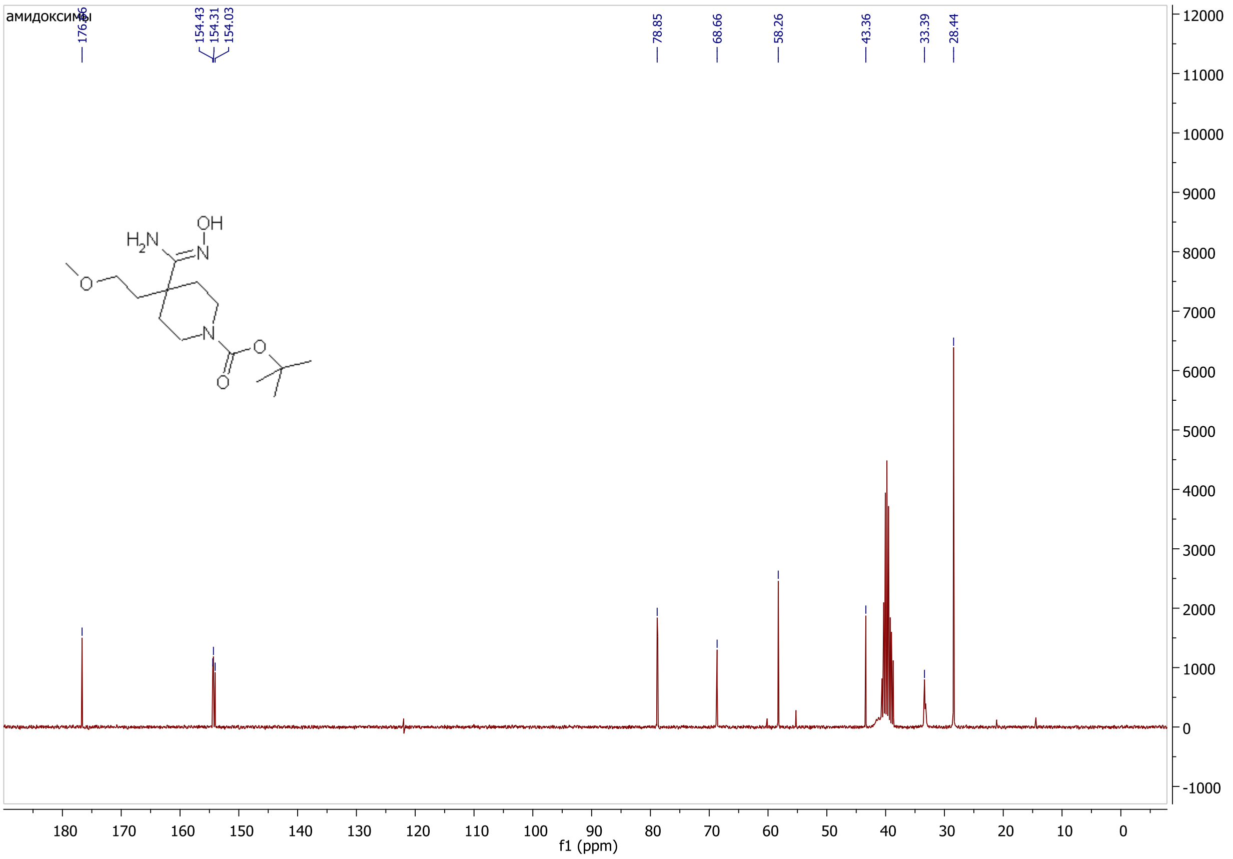

tert-butyl 3-[(hydroxyamino)(imino)methyl]azetidine-1-carboxylate (1h)

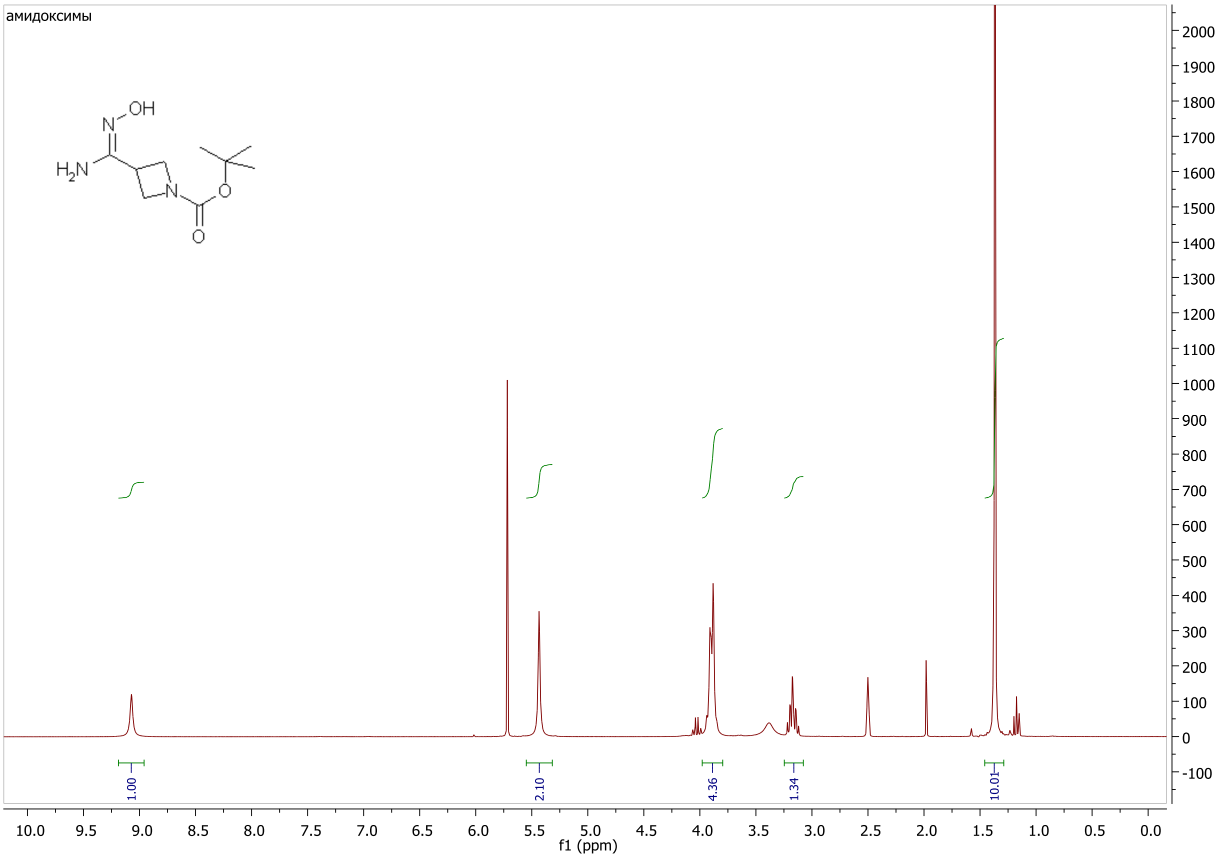

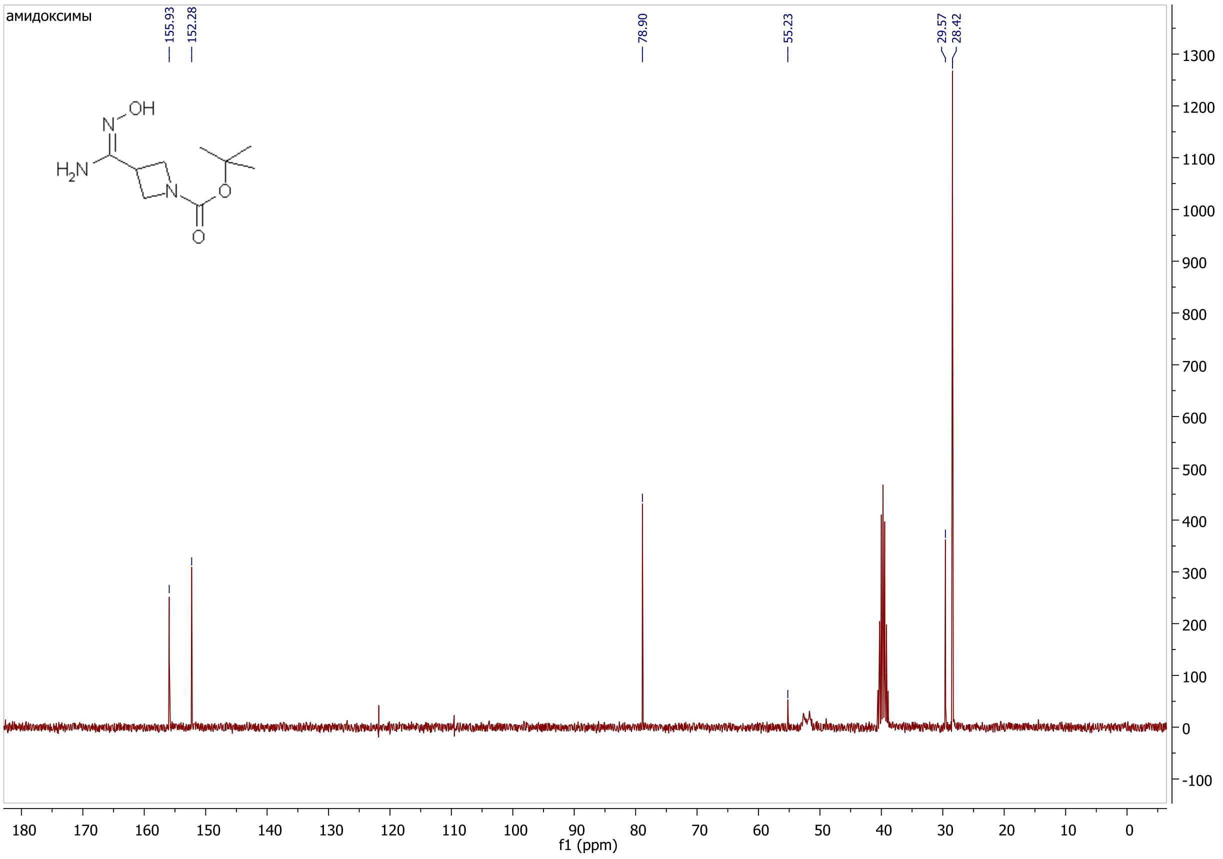

tert-butyl 2-[(hydroxyamino)(imino)methyl]piperidine-1-carboxylate (1i)

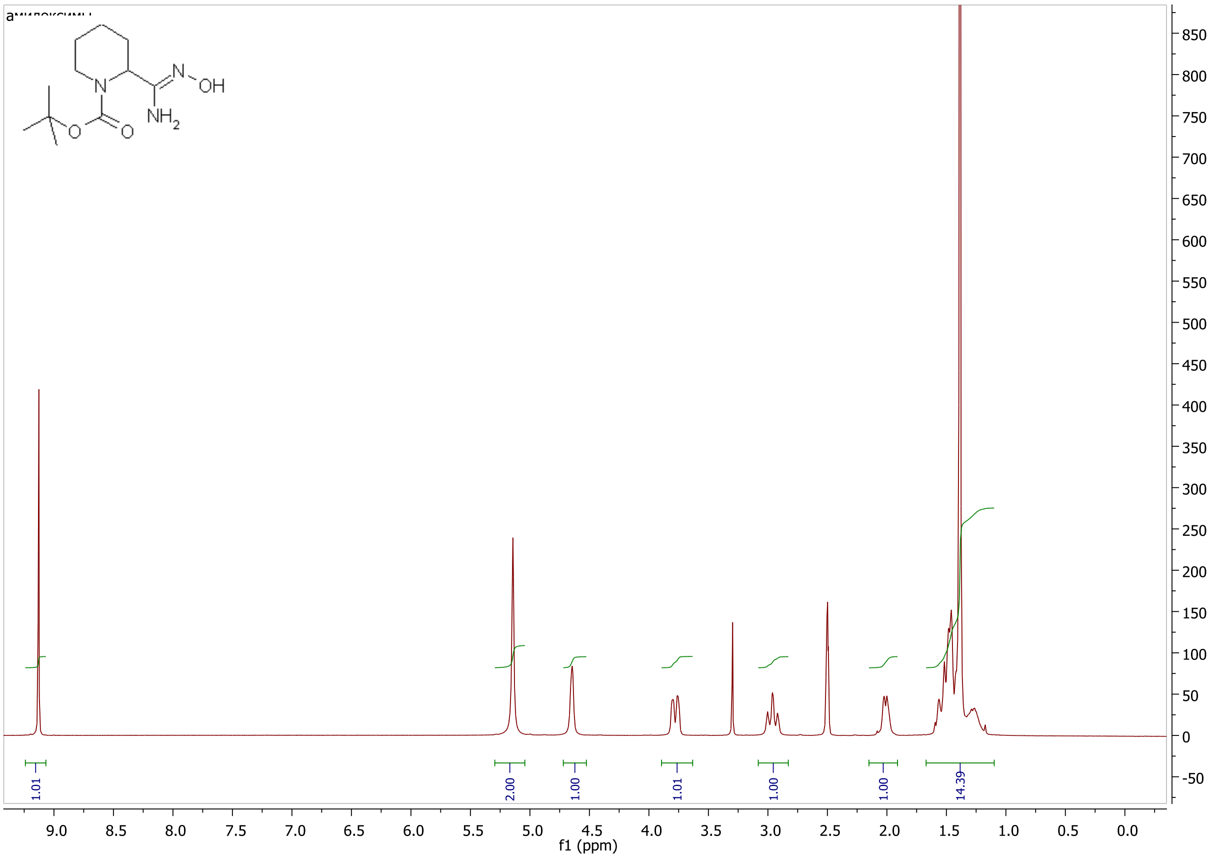

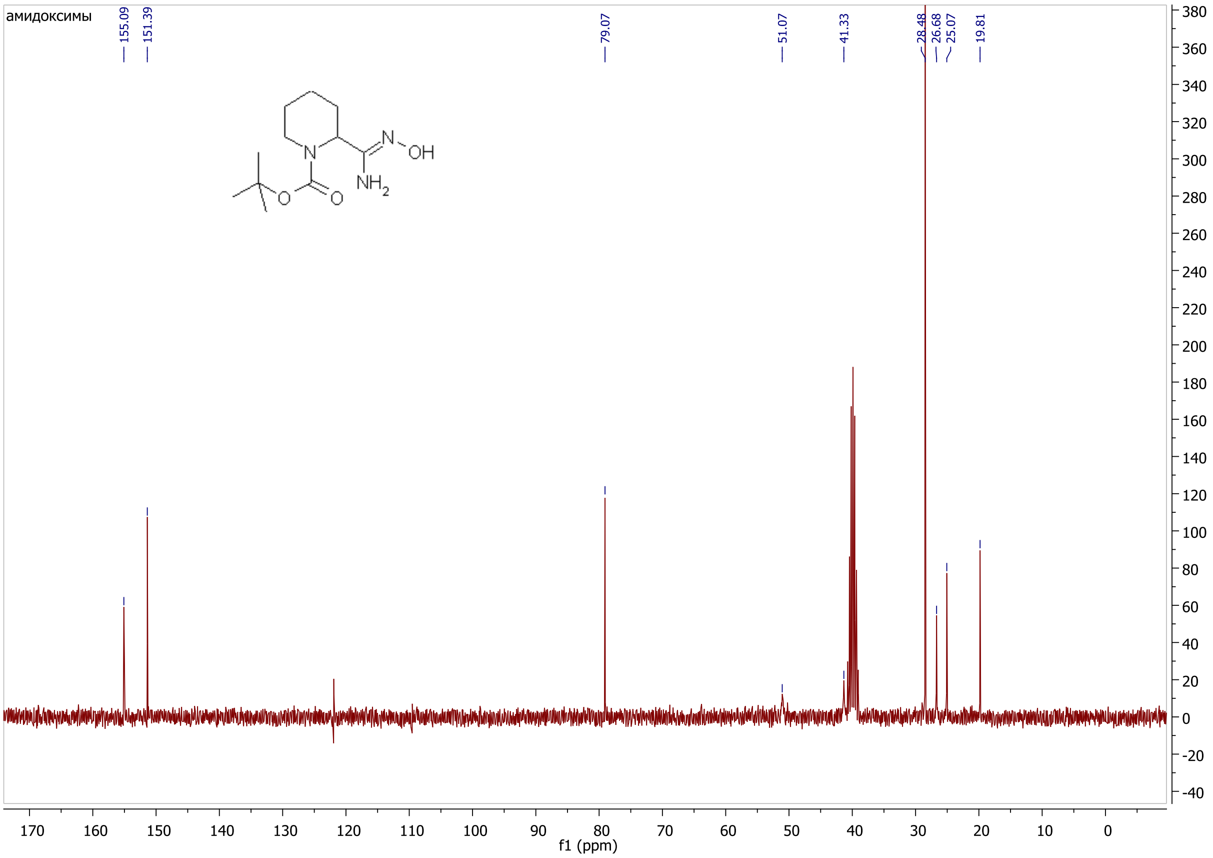

tert-butyl 4-[(hydroxyamino)(imino)methyl]-4-methylpiperidine-1-carboxylate (1j)

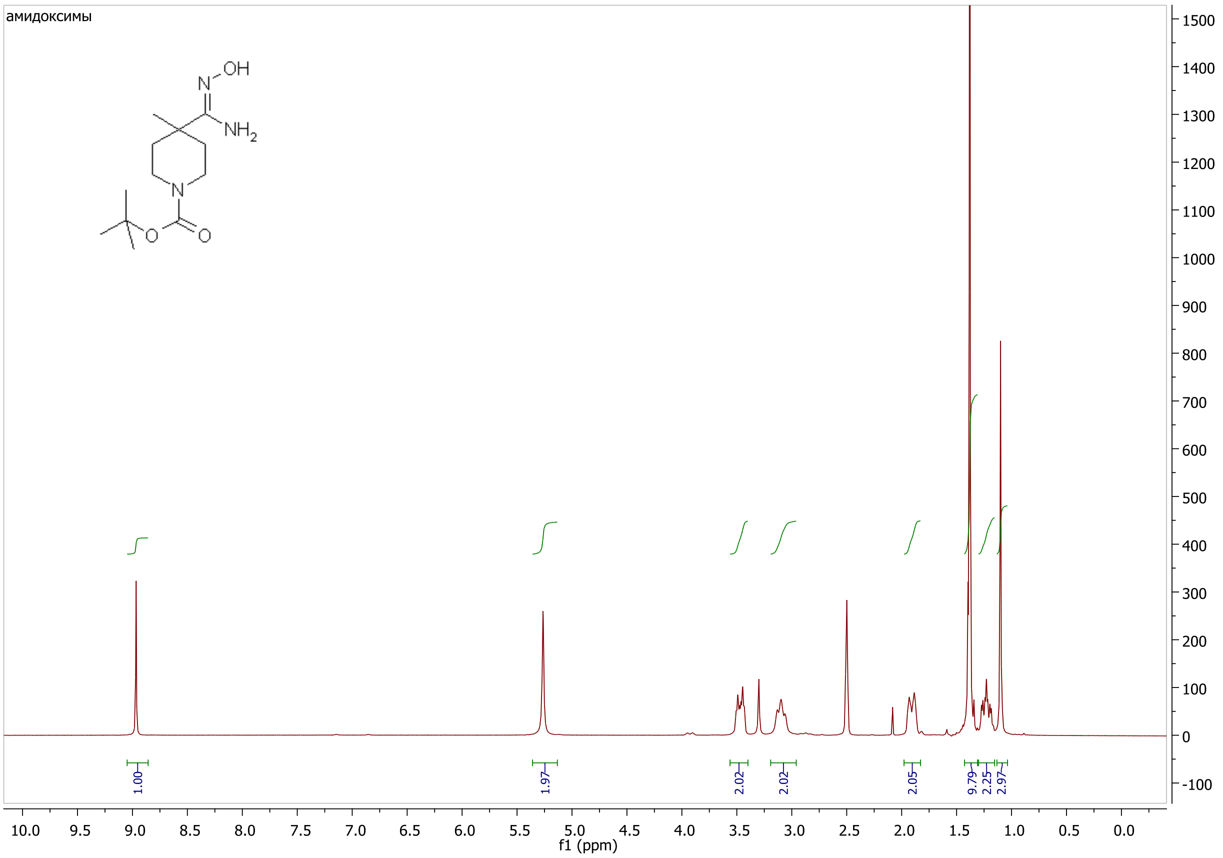

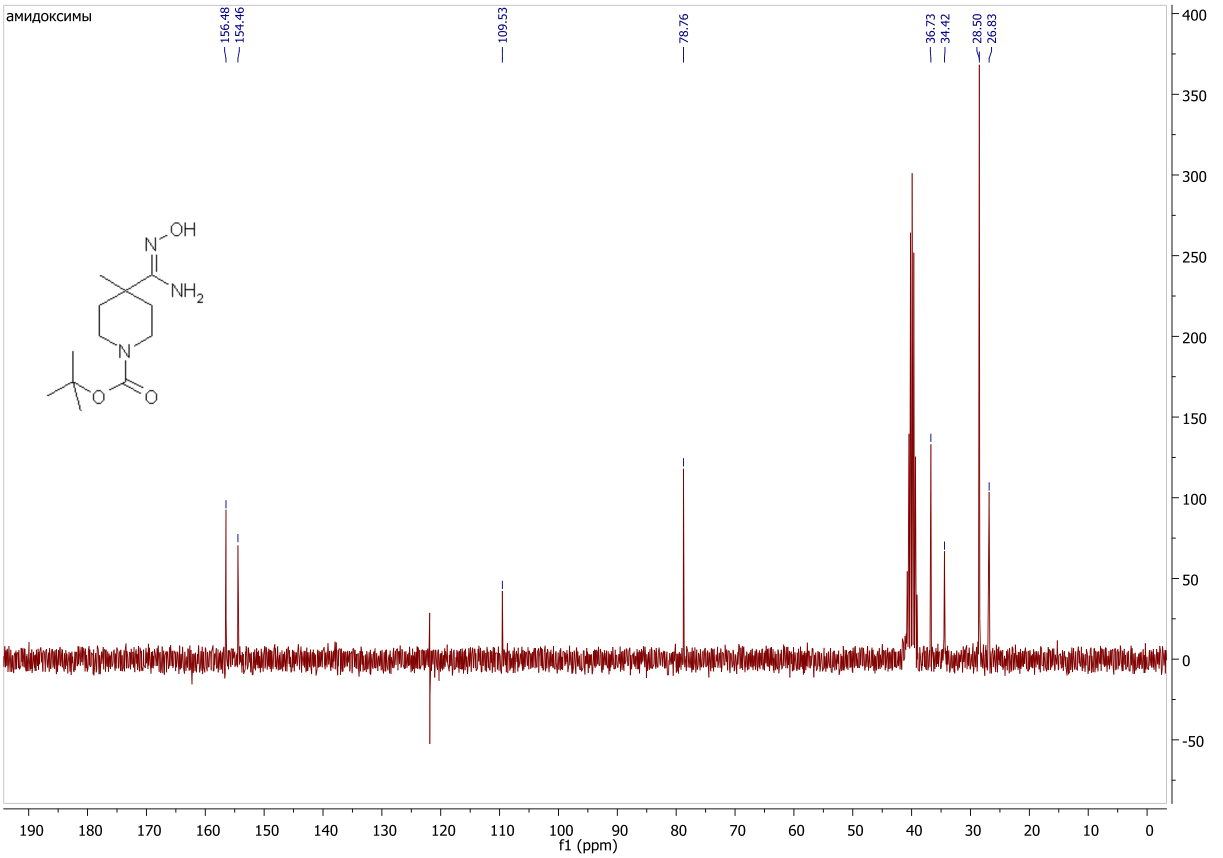

tert-butyl 3-[2-(hydroxyamino)-2-iminoethyl]pyrrolidine-1-carboxylate (1k)

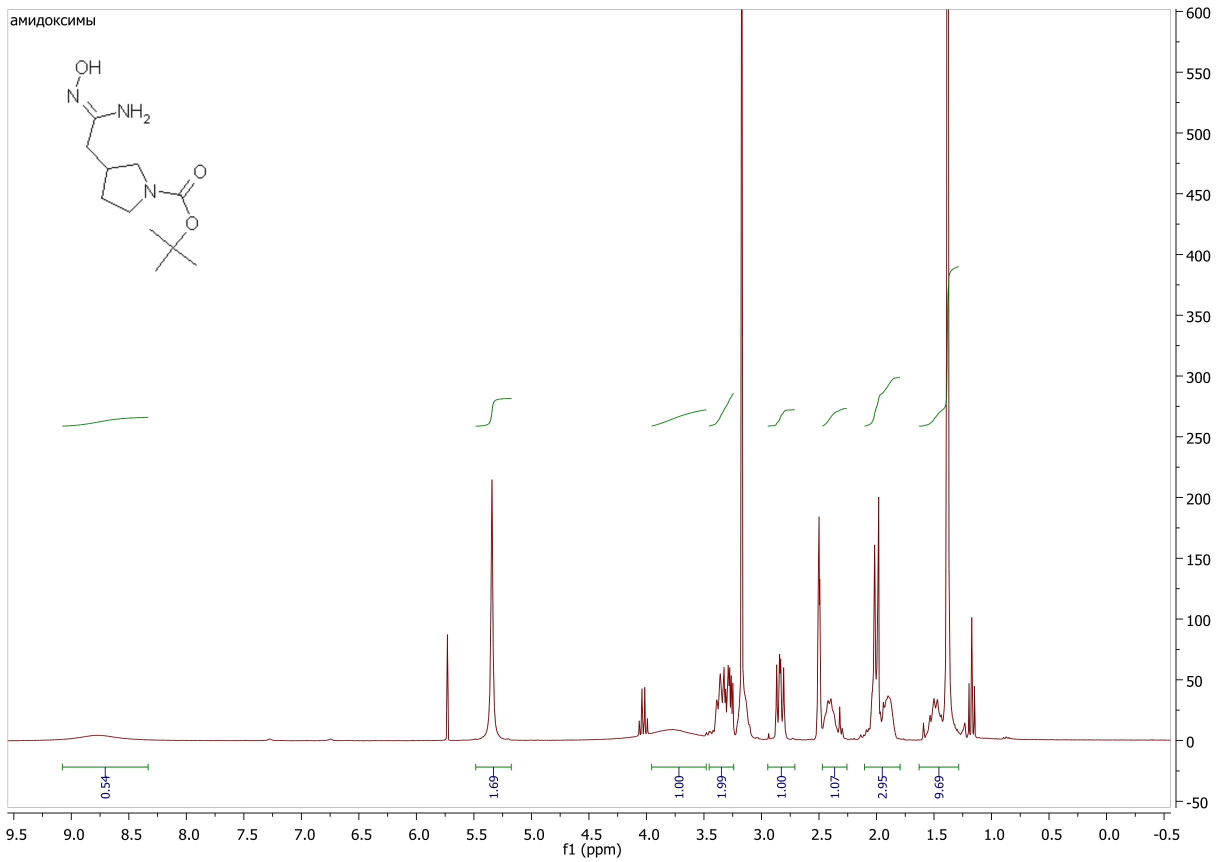

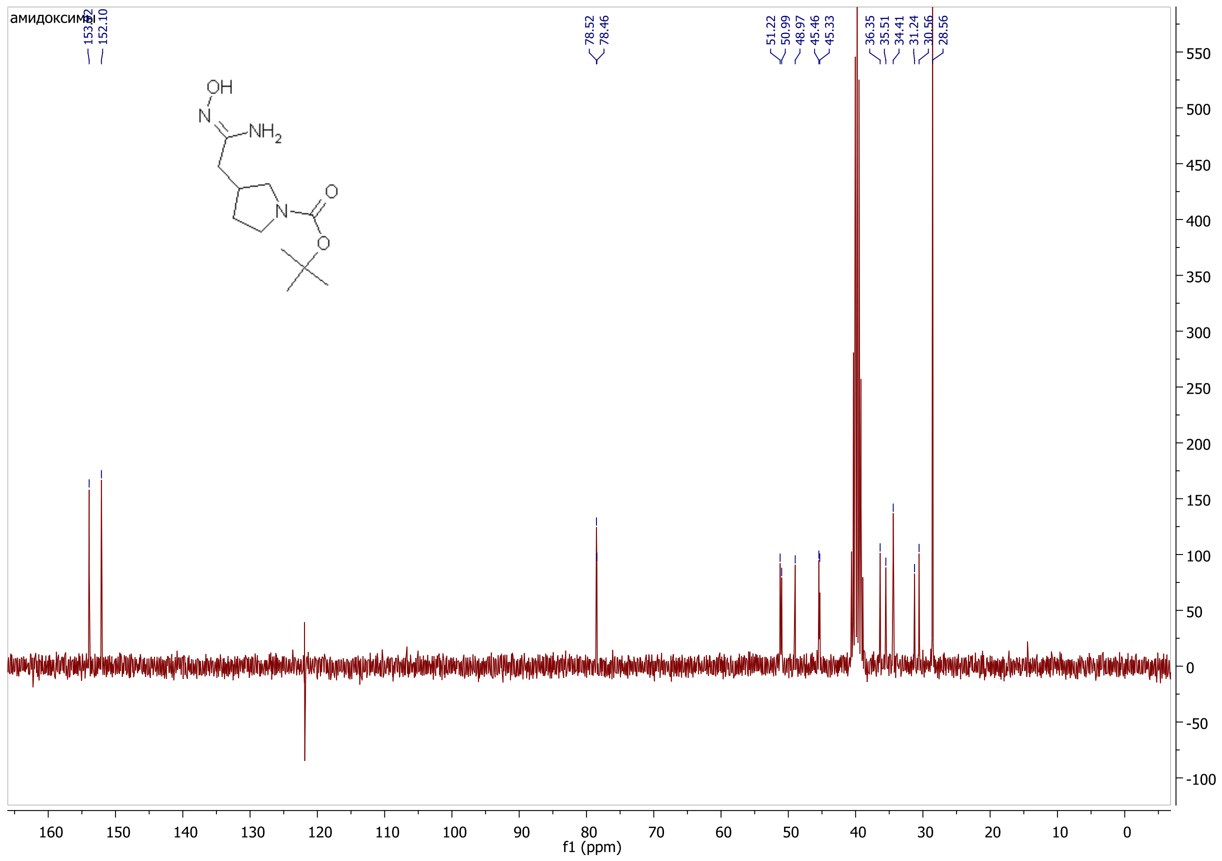

tert-butyl 3-[(hydroxyamino)(imino)methyl]pyrrolidine-1-carboxylate (1l)

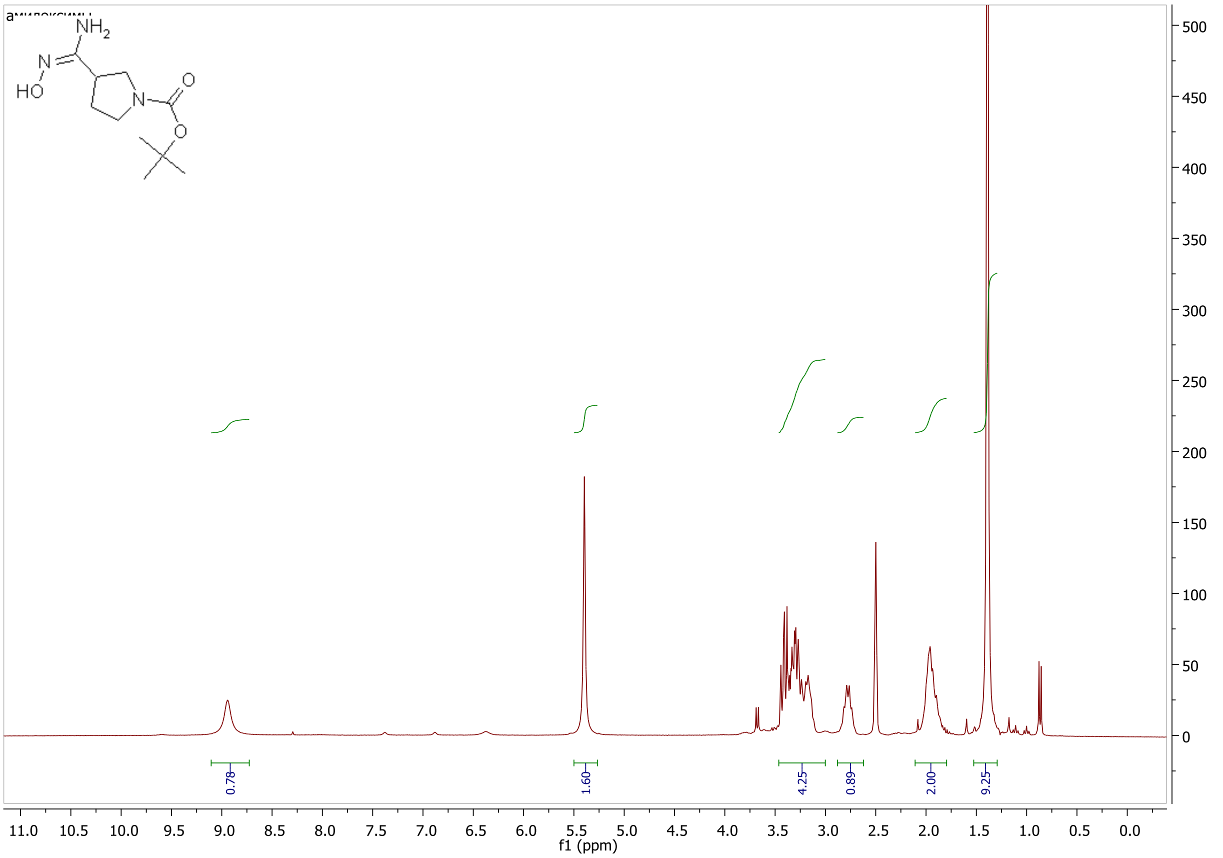

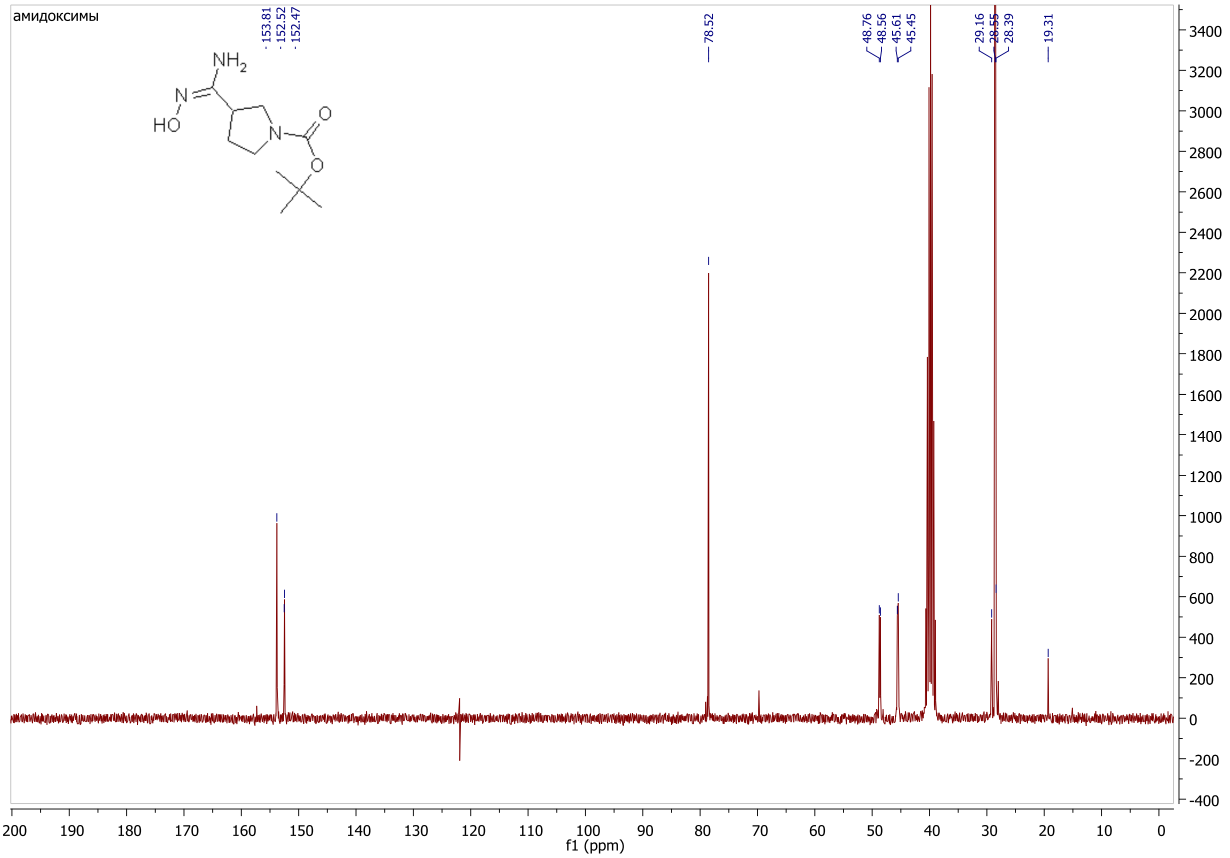

tert-butyl 3-[(hydroxyamino)(imino)methyl]-3-(2-methoxyethyl)piperidine-1-carboxylate (1m)

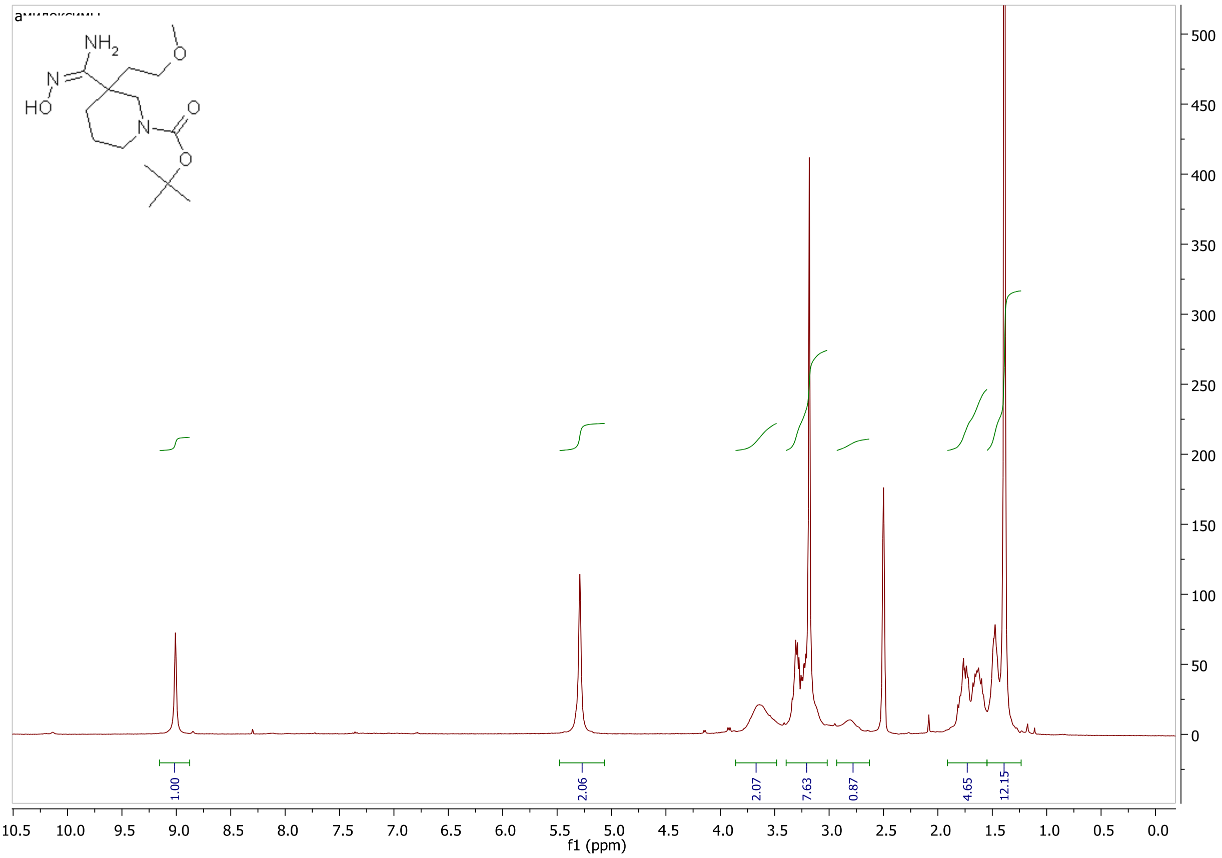

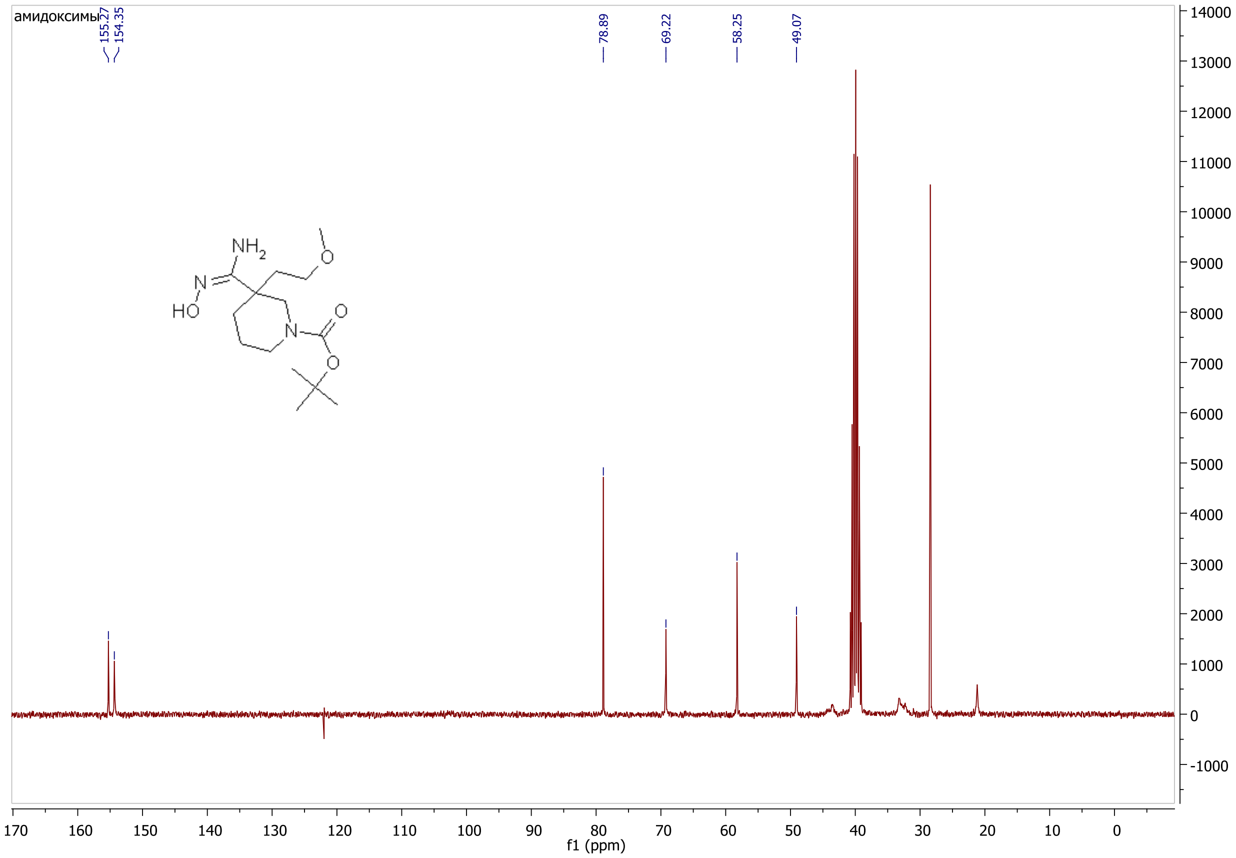

4-[5-(5-nitro-2-furyl)-1,2,4-oxadiazol-3-yl]piperidine hydrochloride (2a)

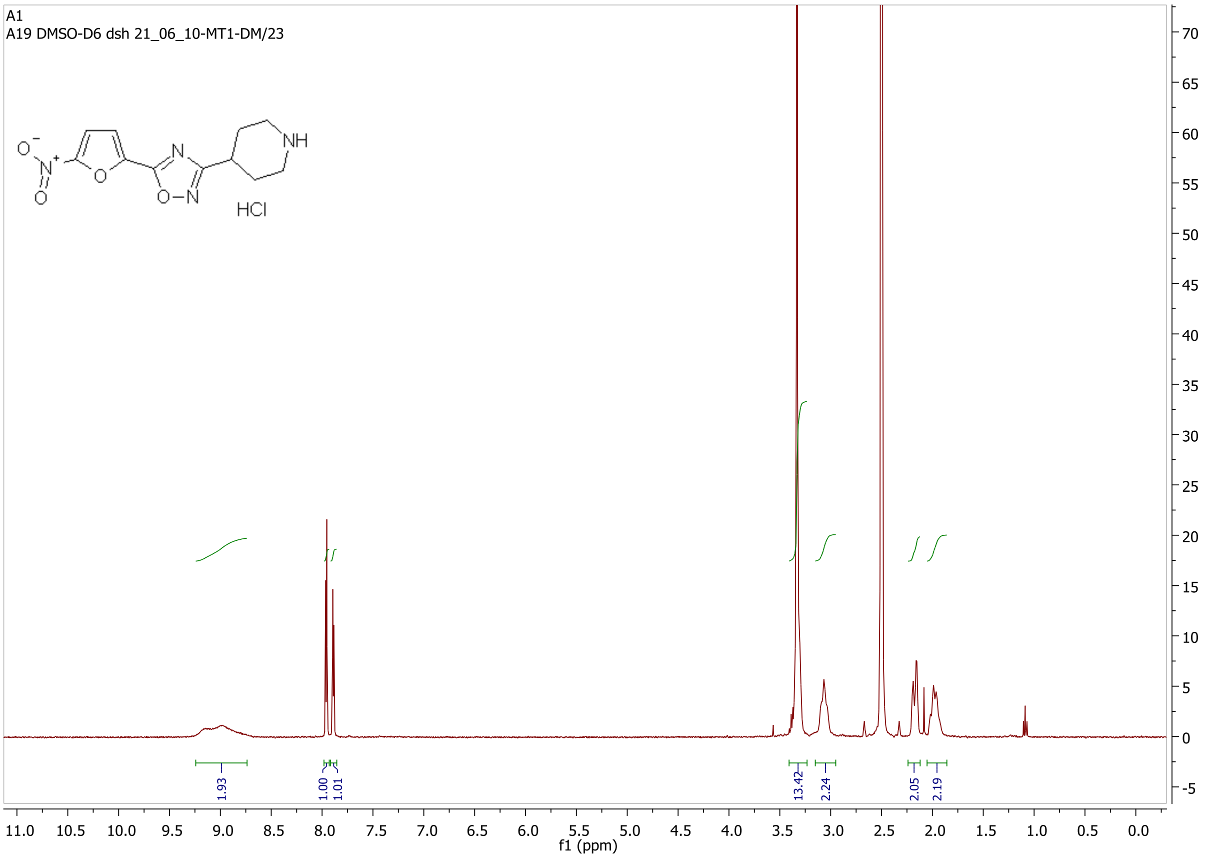

^{13}C NMR (75 MHz, DMSO) δ 173.00, 166.00, 153.38, 140.29, 119.43, 114.20, 42.50, 31.12, 26.20.

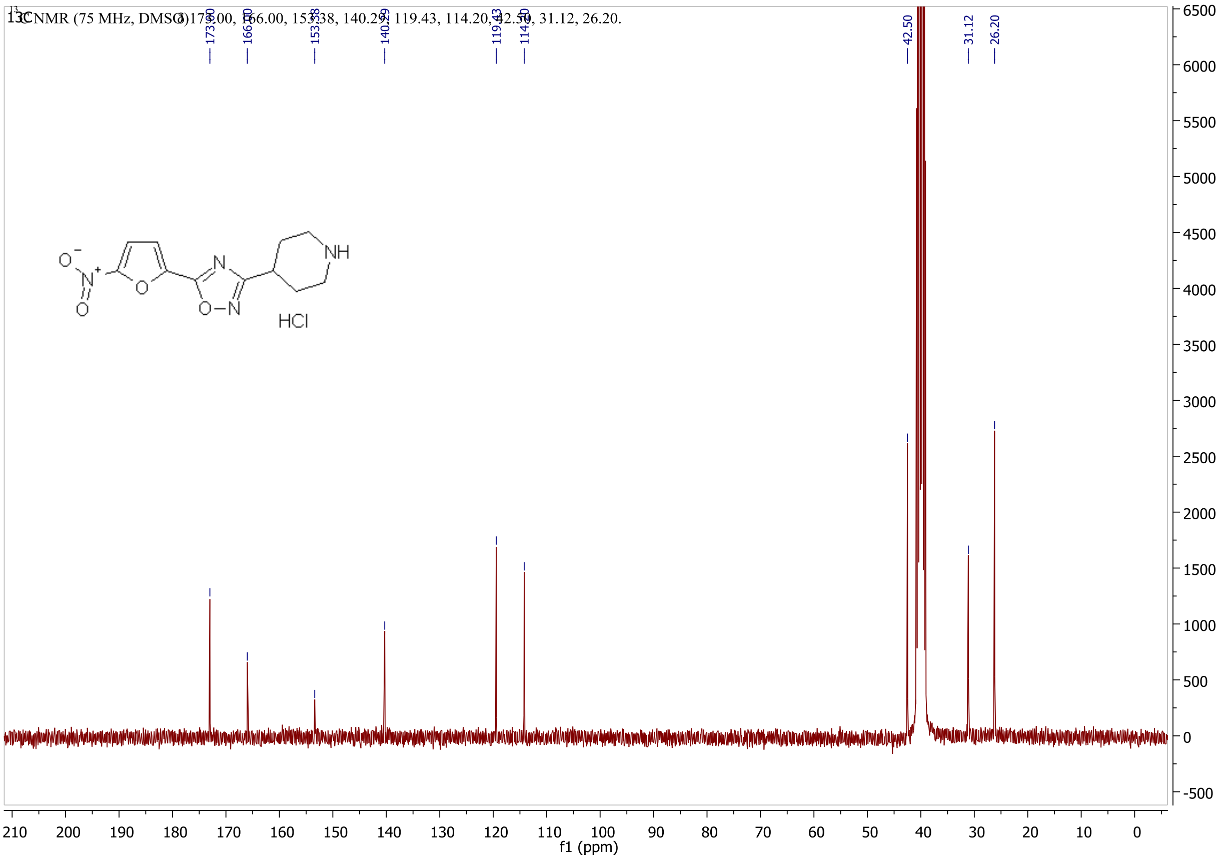

3-[5-(5-nitro-2-furyl)-1,2,4-oxadiazol-3-yl]piperidine hydrochloride (2b)

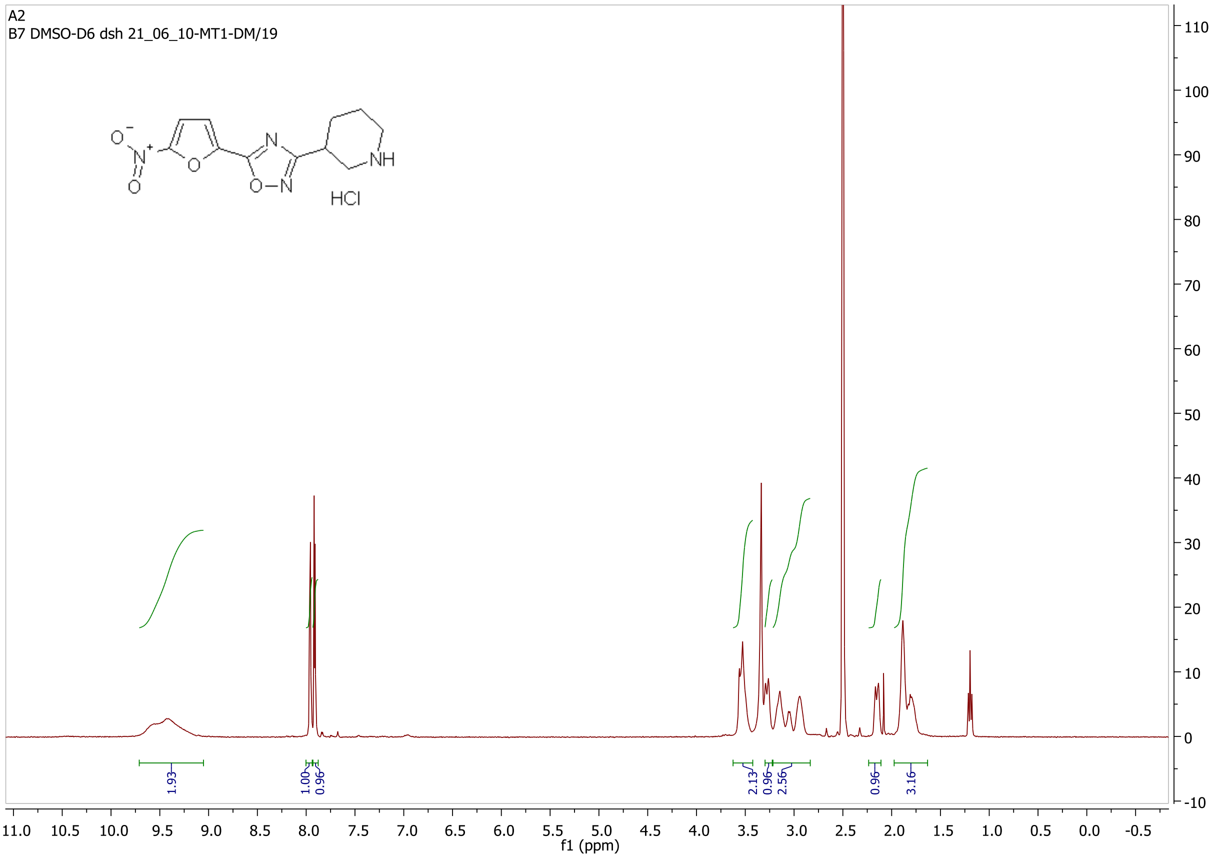

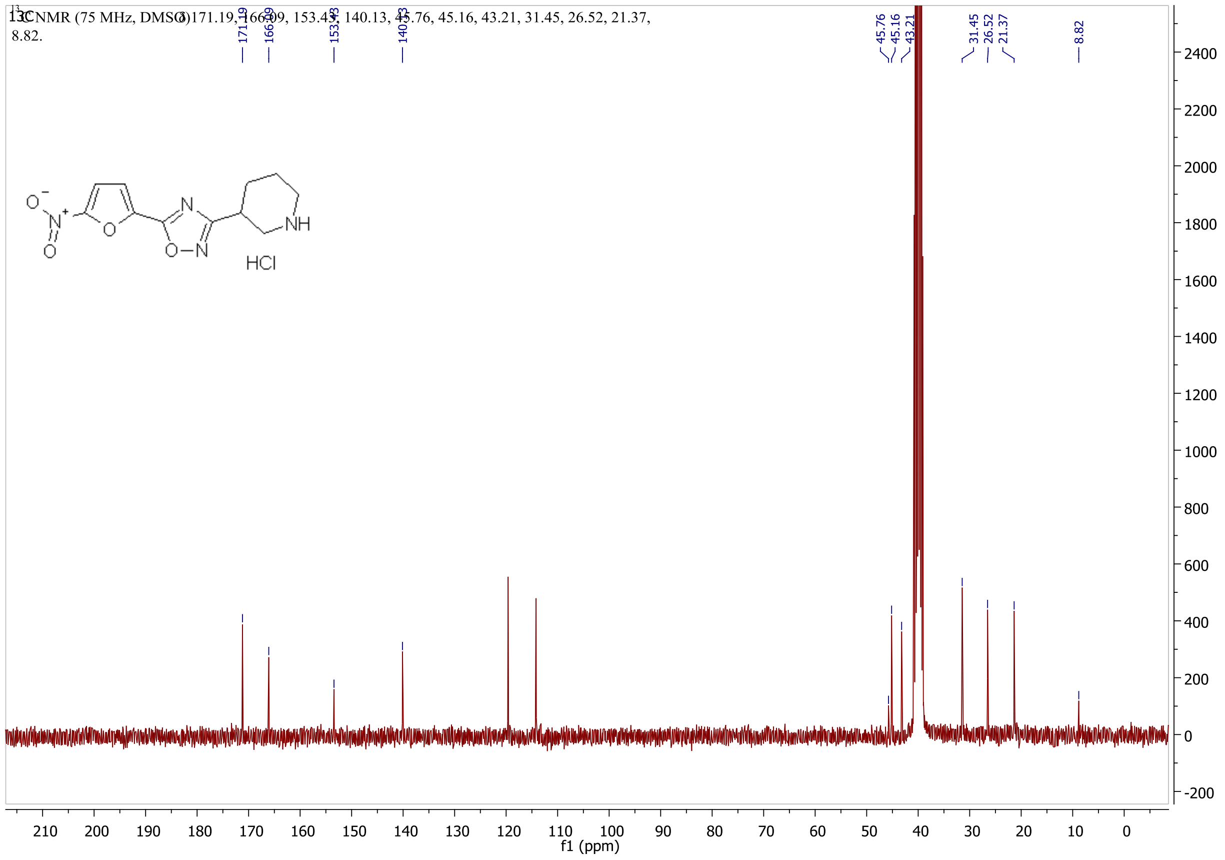

N-methyl-2-[5-(5-nitro-2-furyl)-1,2,4-oxadiazol-3-yl]ethanamine hydrochloride (2c)

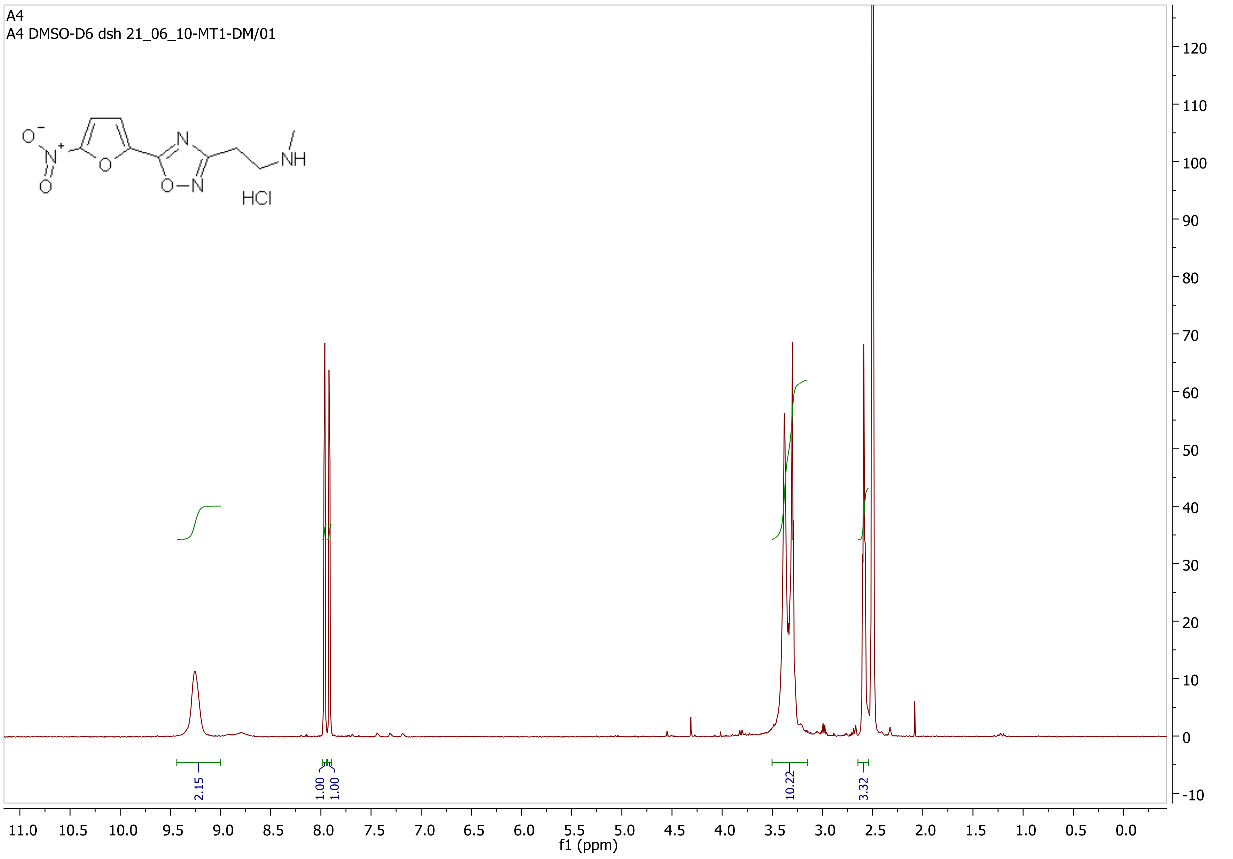

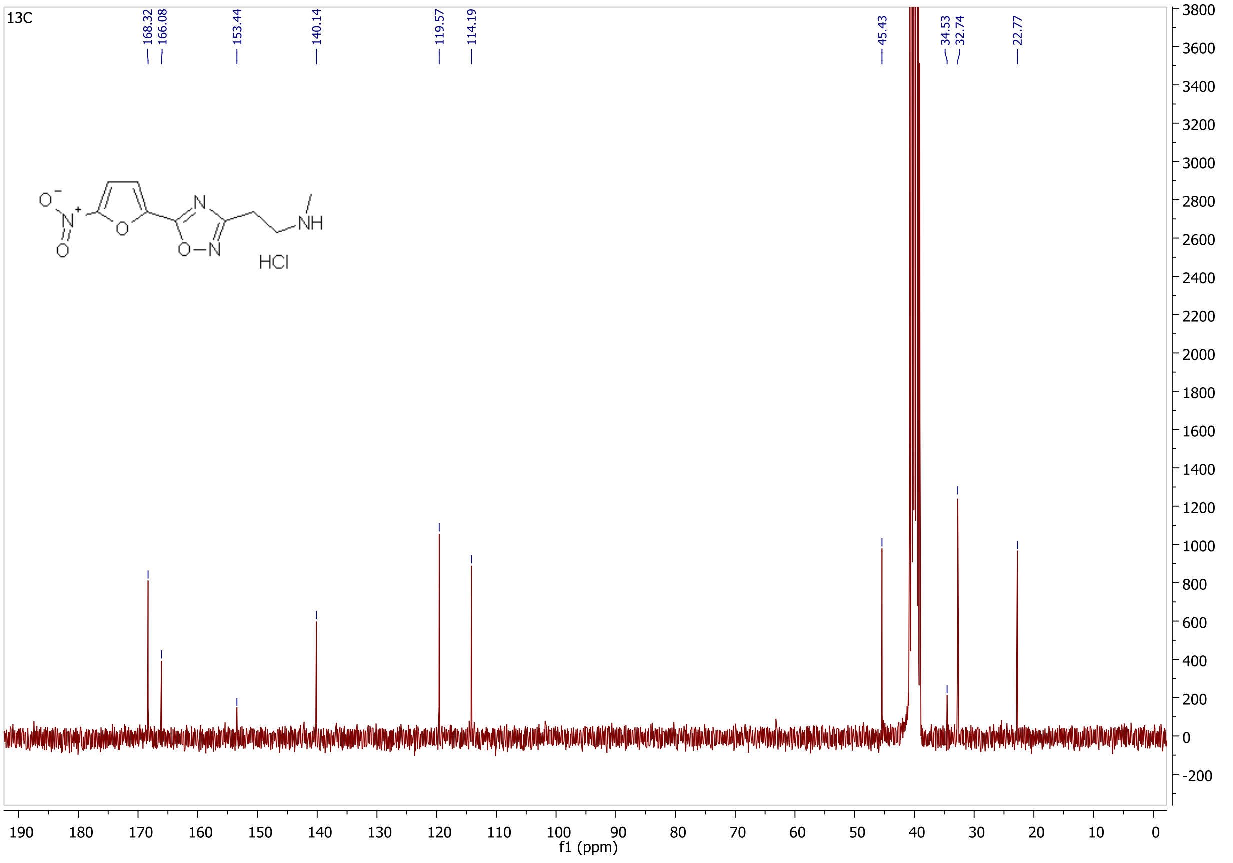

N-methyl-1-[5-(5-nitro-2-furyl)-1,2,4-oxadiazol-3-yl]methanamine hydrochloride (2d)

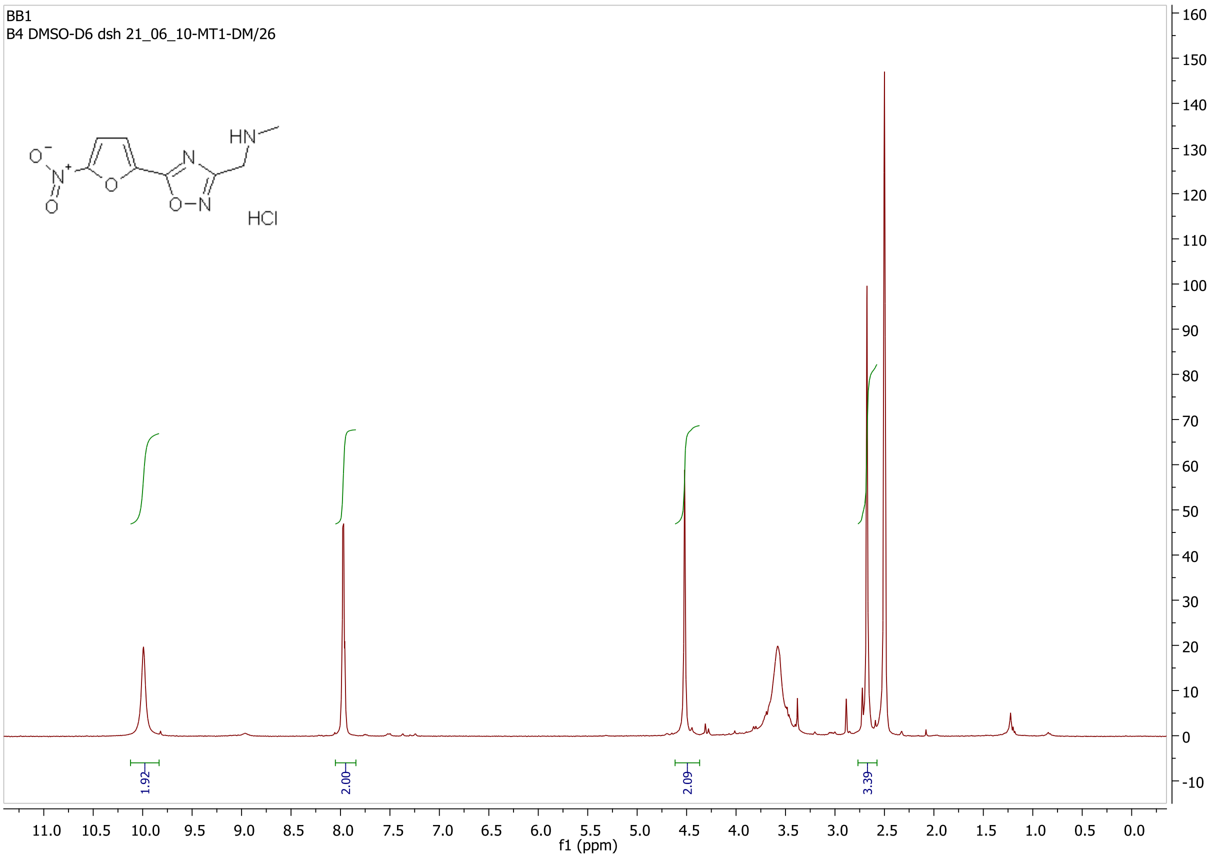

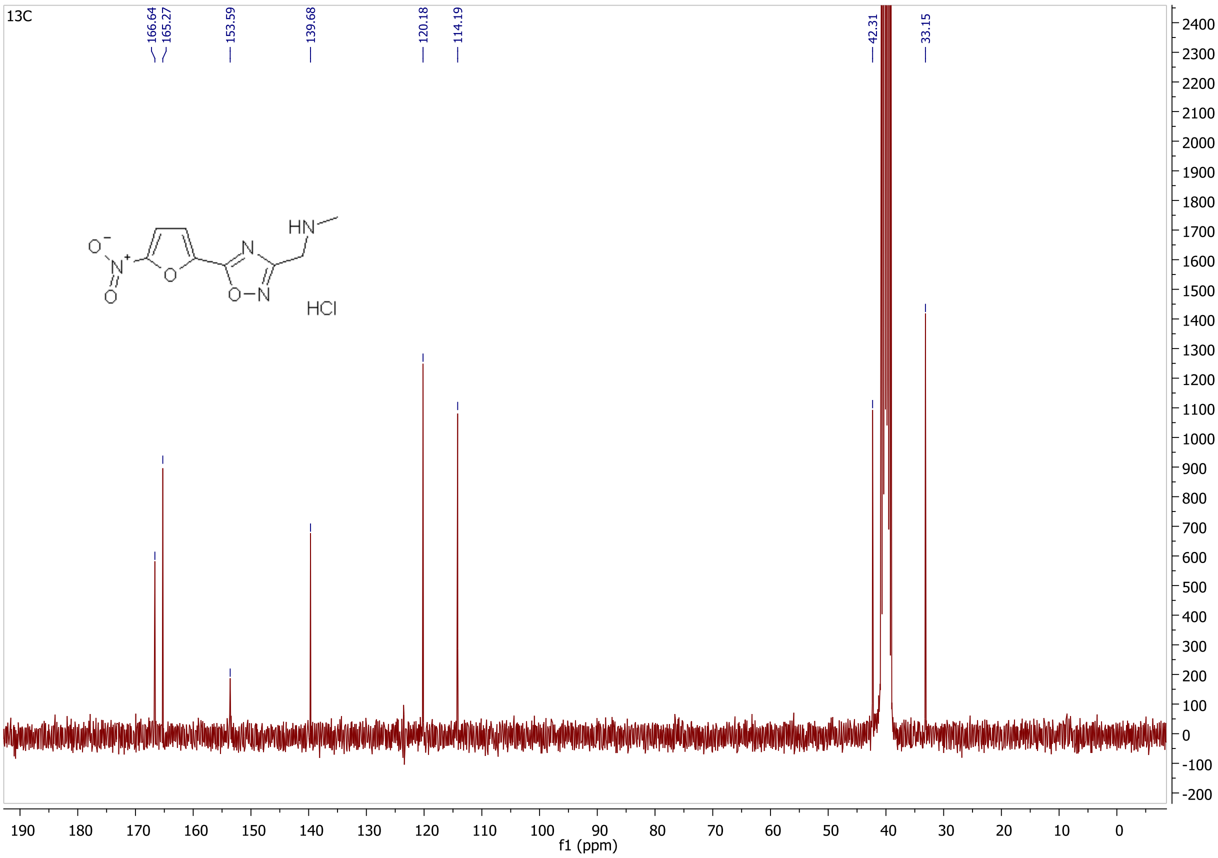

5-(5-nitro-2-furyl)-3-[4-(pyrrolidin-3-yloxy)phenyl]-1,2,4-oxadiazole hydrochloride (2e)

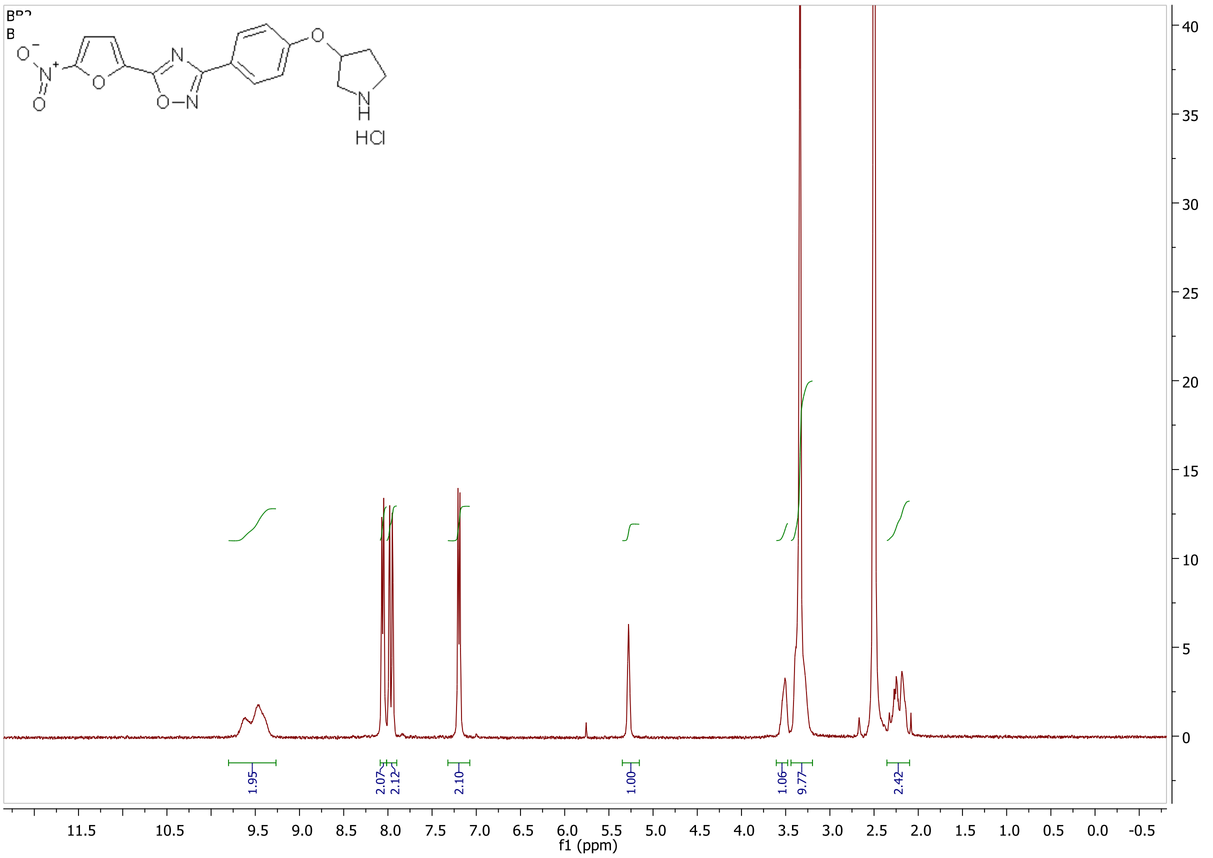

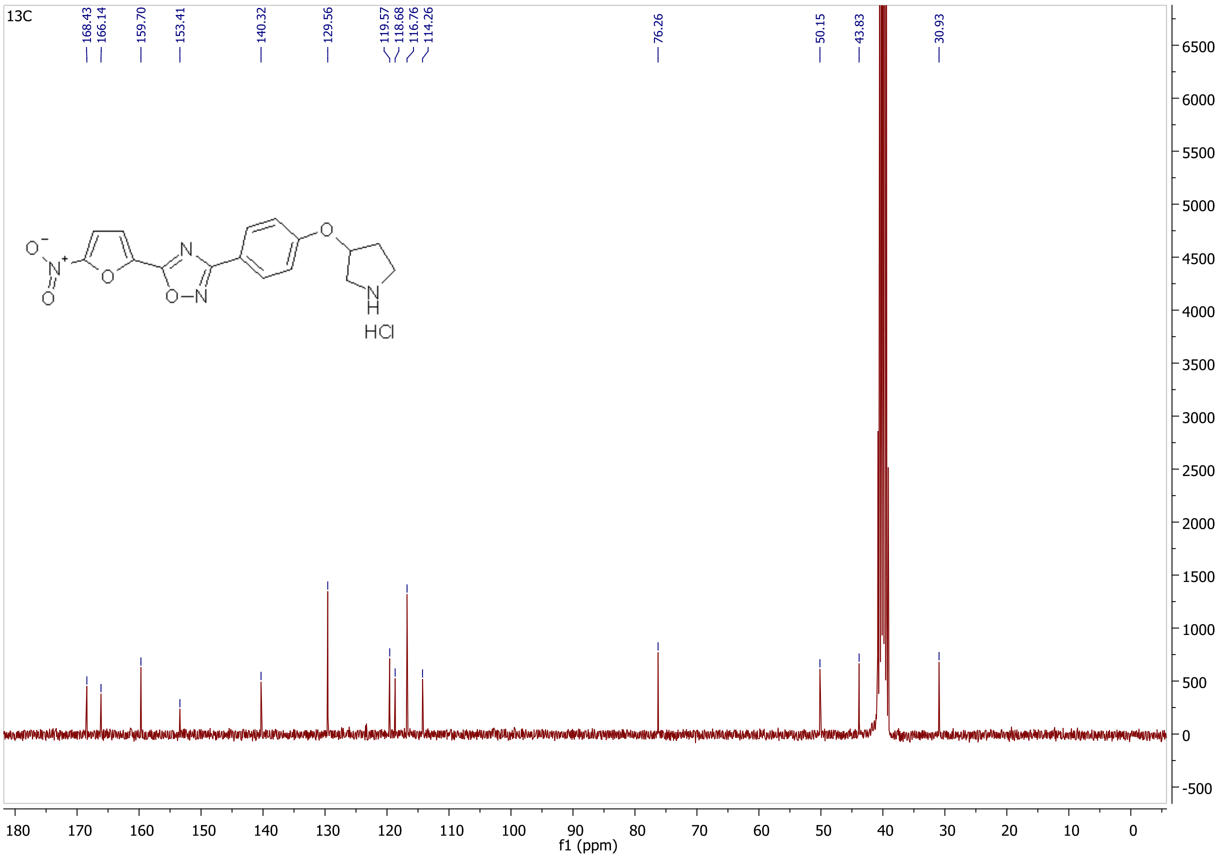

5-(5-nitro-2-furyl)-3-[2-(pyrrolidin-3-yloxy)phenyl]-1,2,4-oxadiazole hydrochloride (2f)

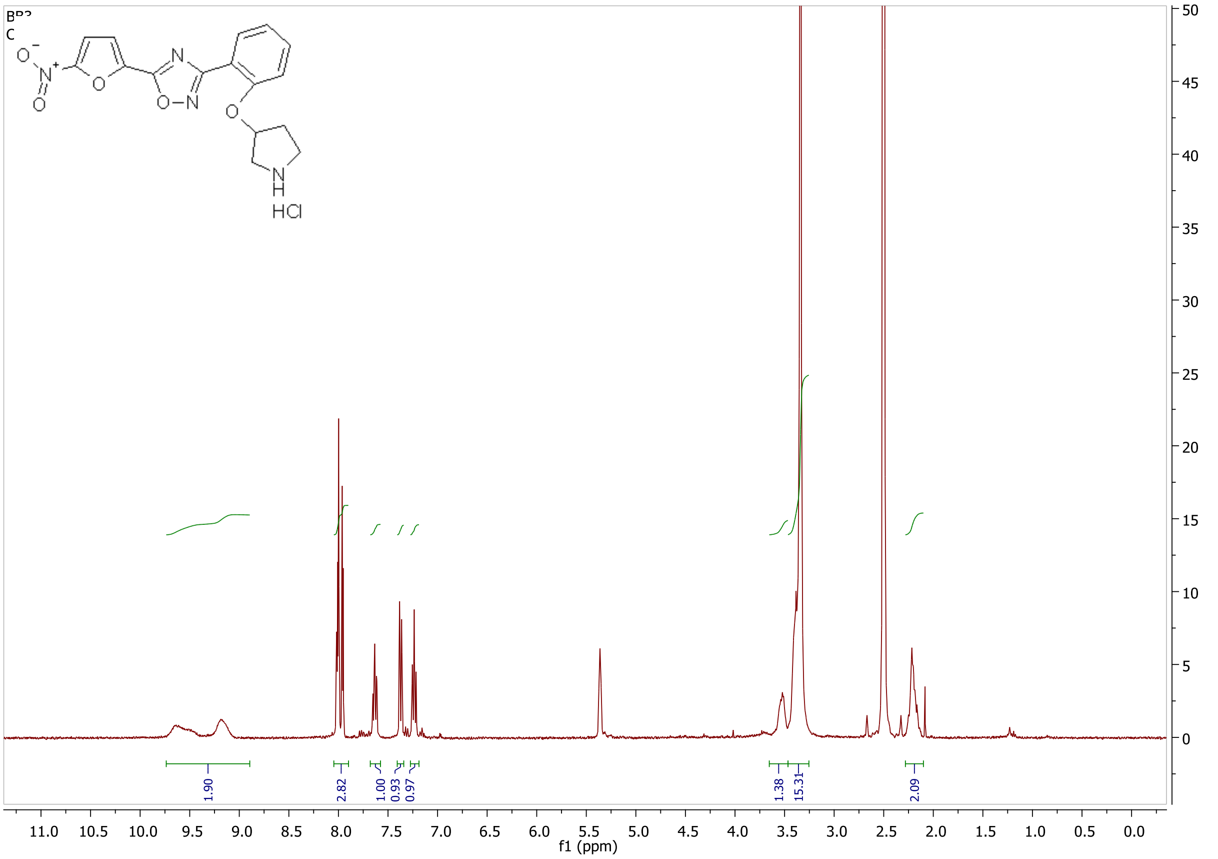

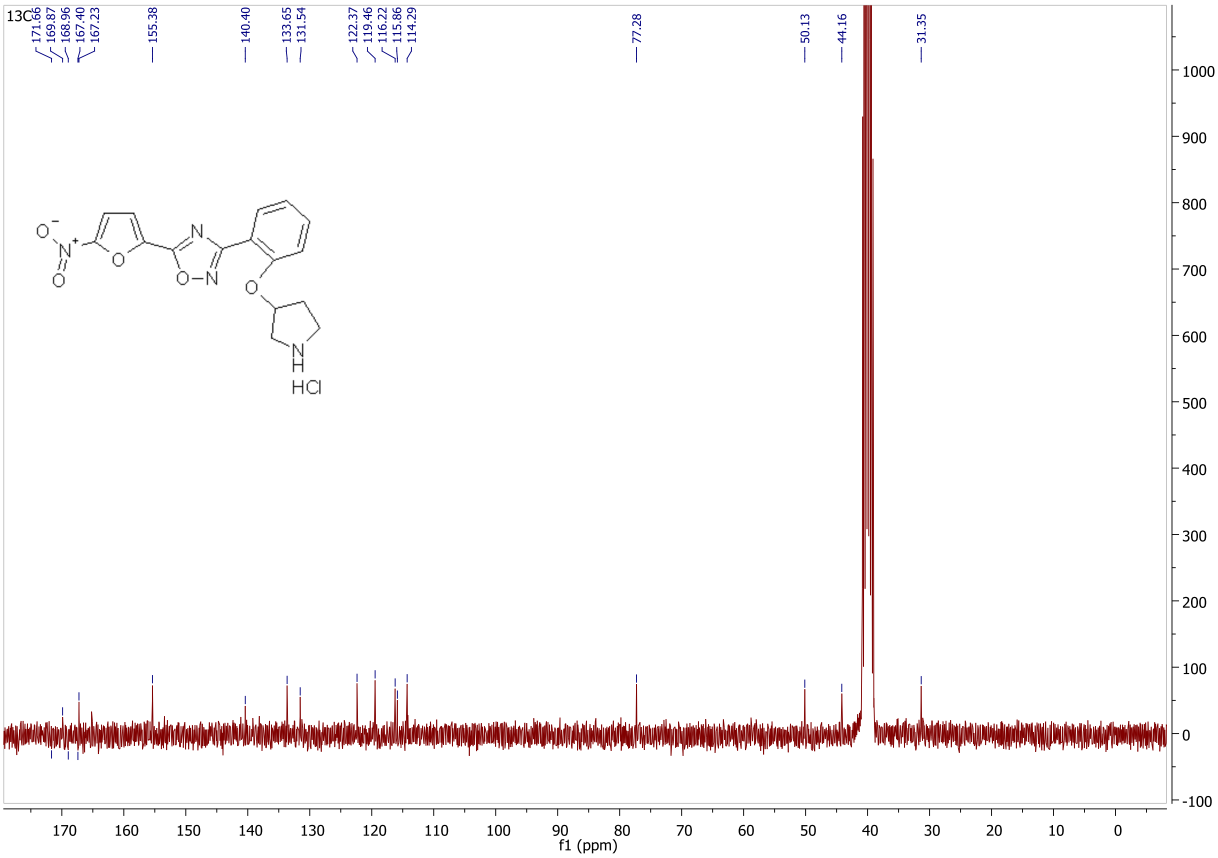

4-(2-methoxyethyl)-4-[5-(5-nitro-2-furyl)-1,2,4-oxadiazol-3-yl]piperidine hydrochloride (2g)

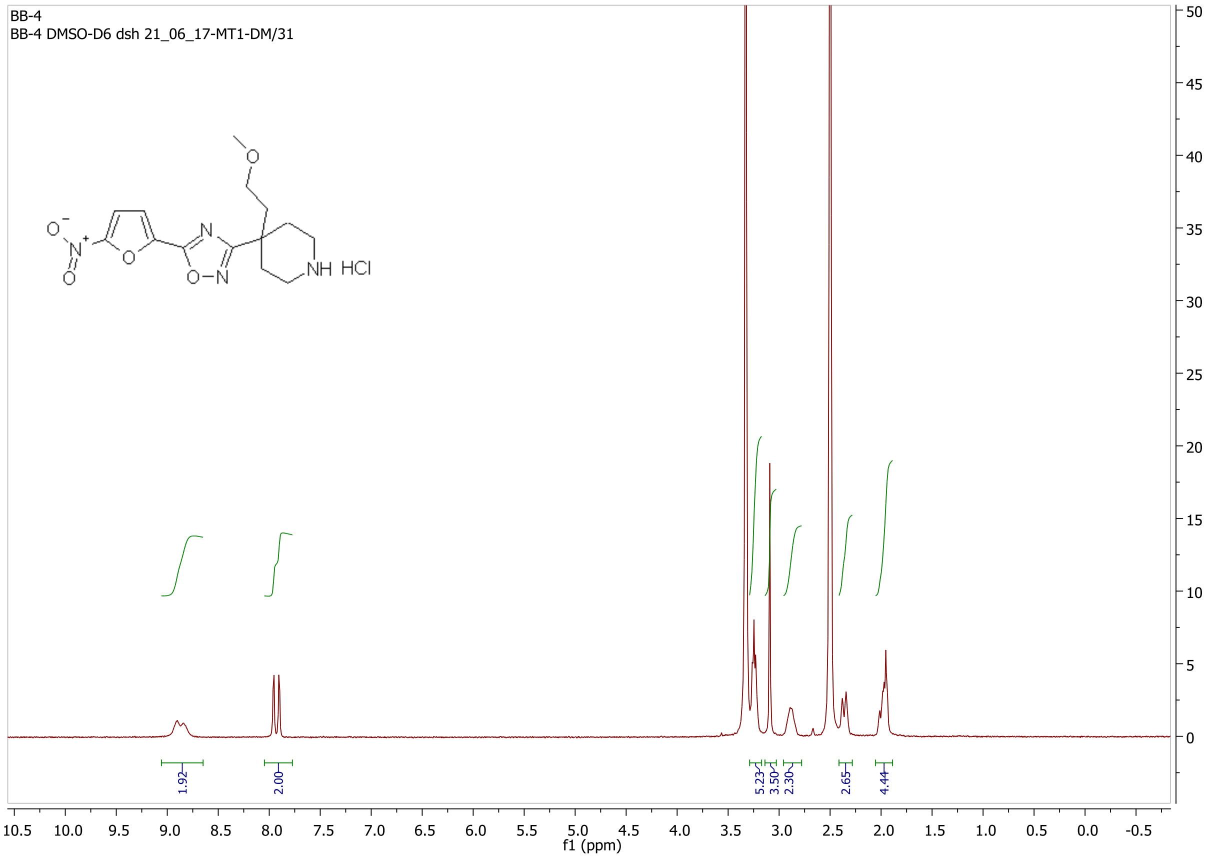

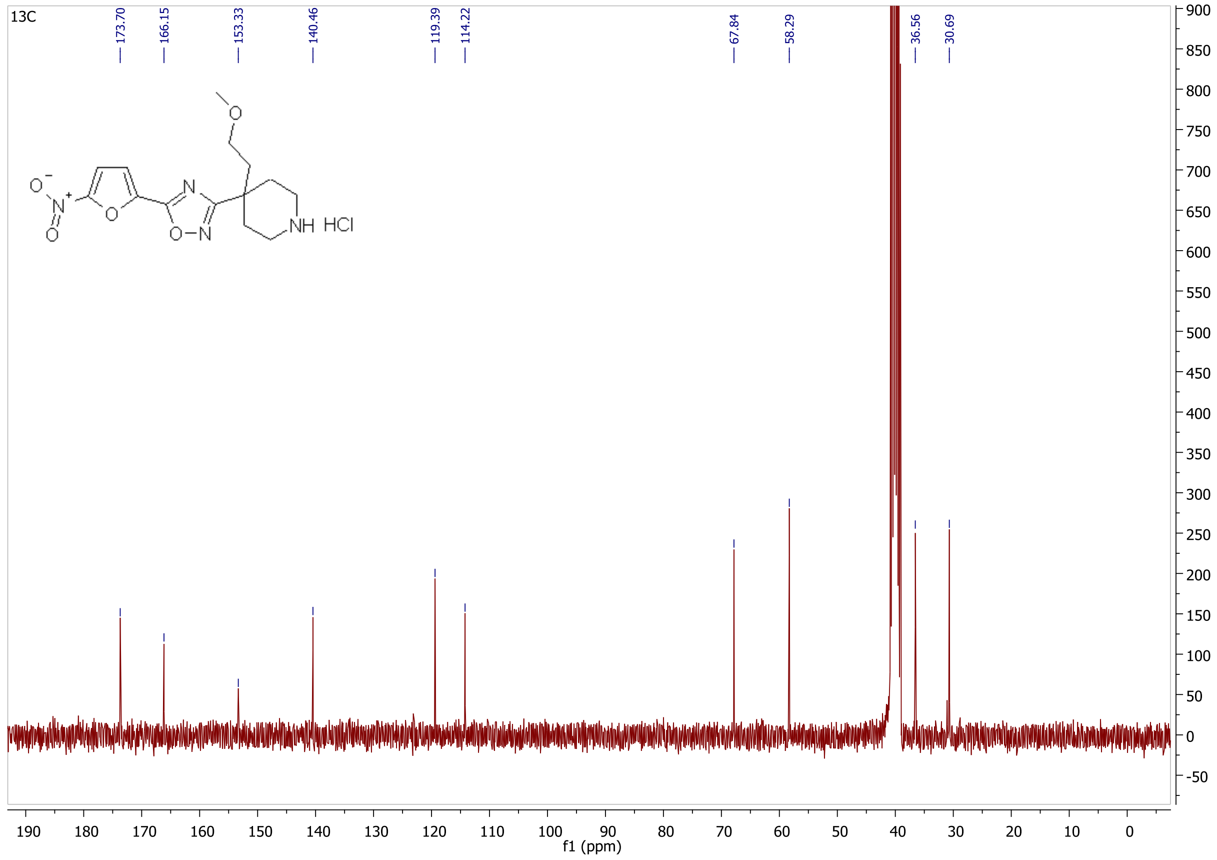

3-azetidin-3-yl-5-(5-nitro-2-furyl)-1,2,4-oxadiazole hydrochloride (2h)

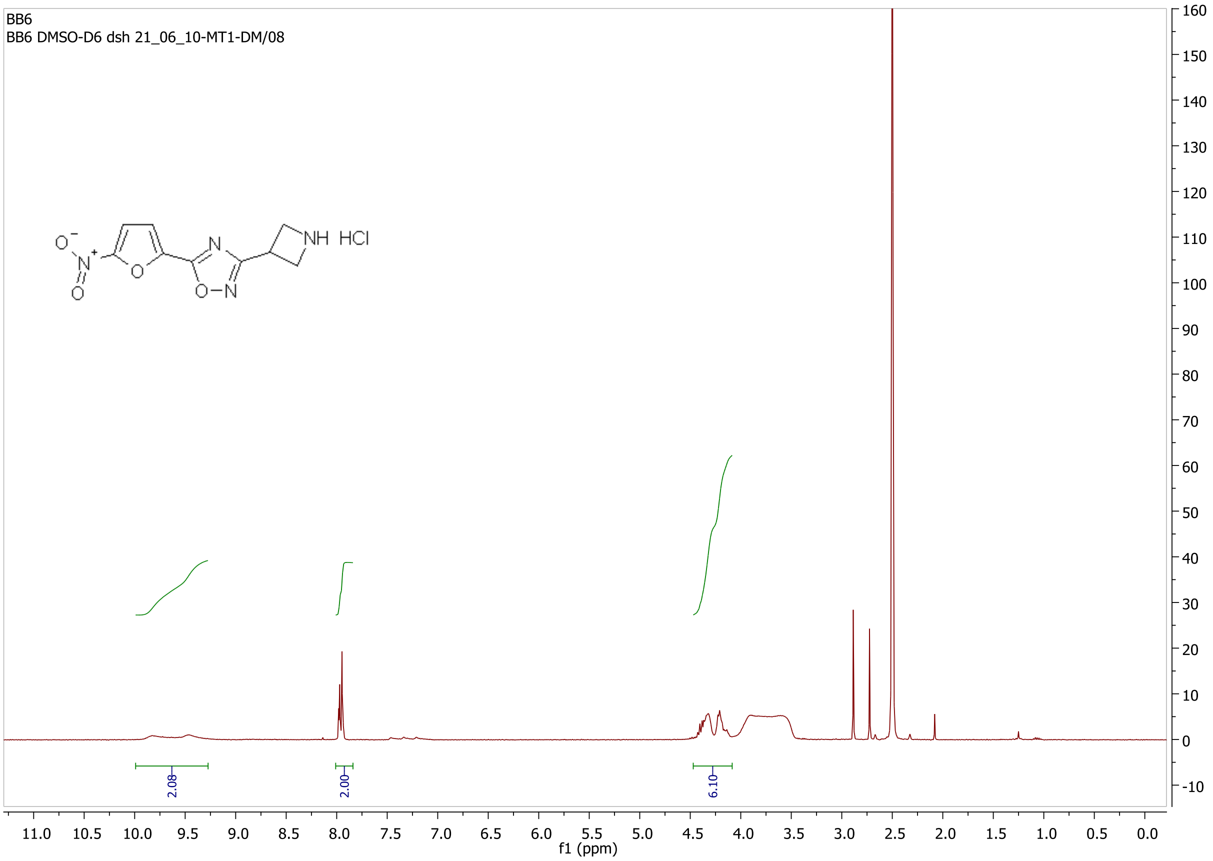

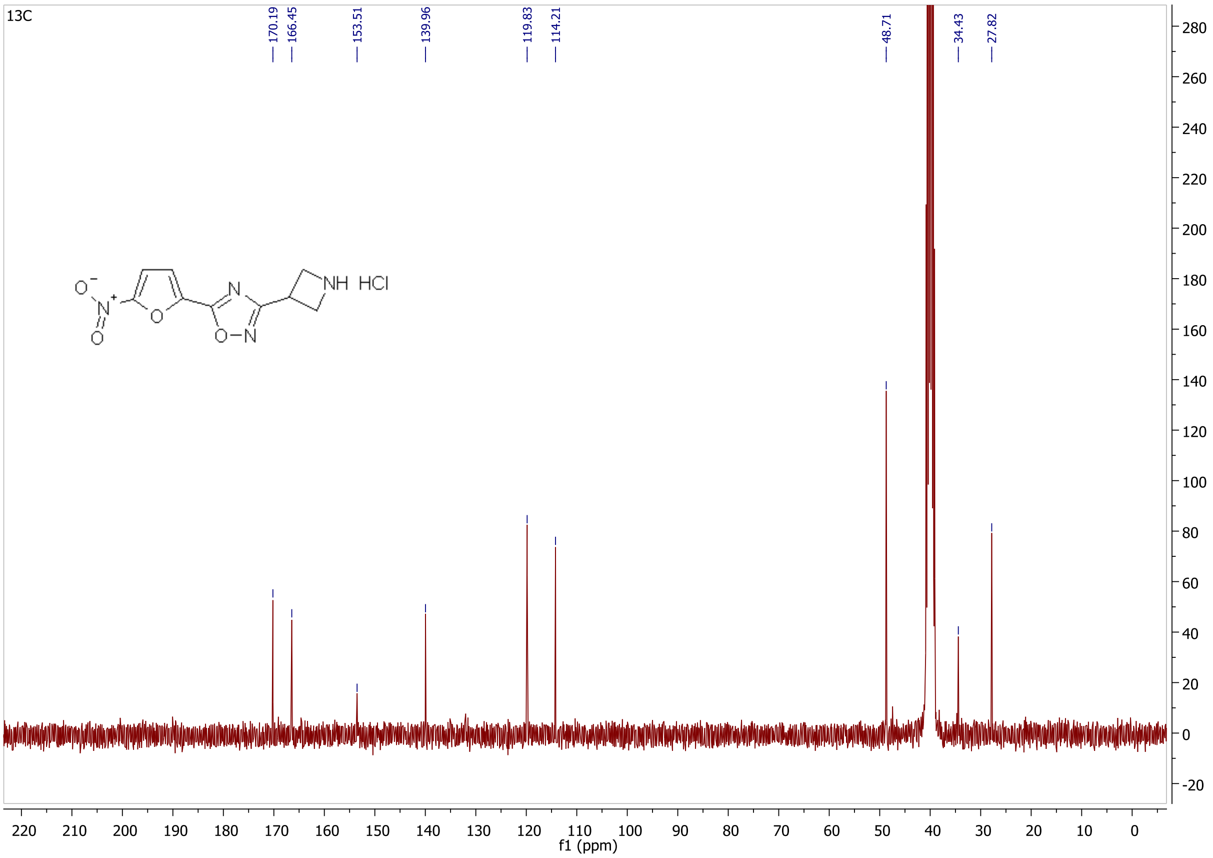

2-[5-(5-nitro-2-furyl)-1,2,4-oxadiazol-3-yl]piperidine hydrochloride (2i)

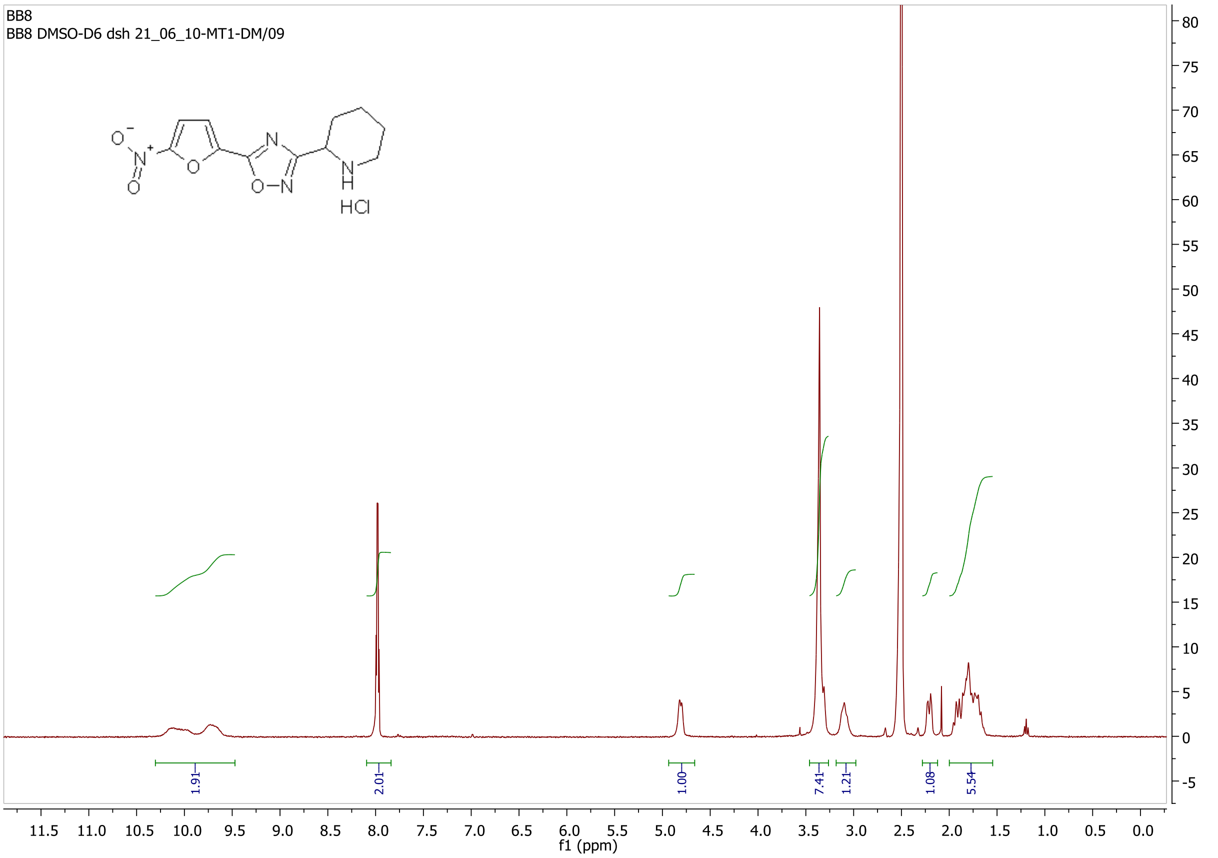

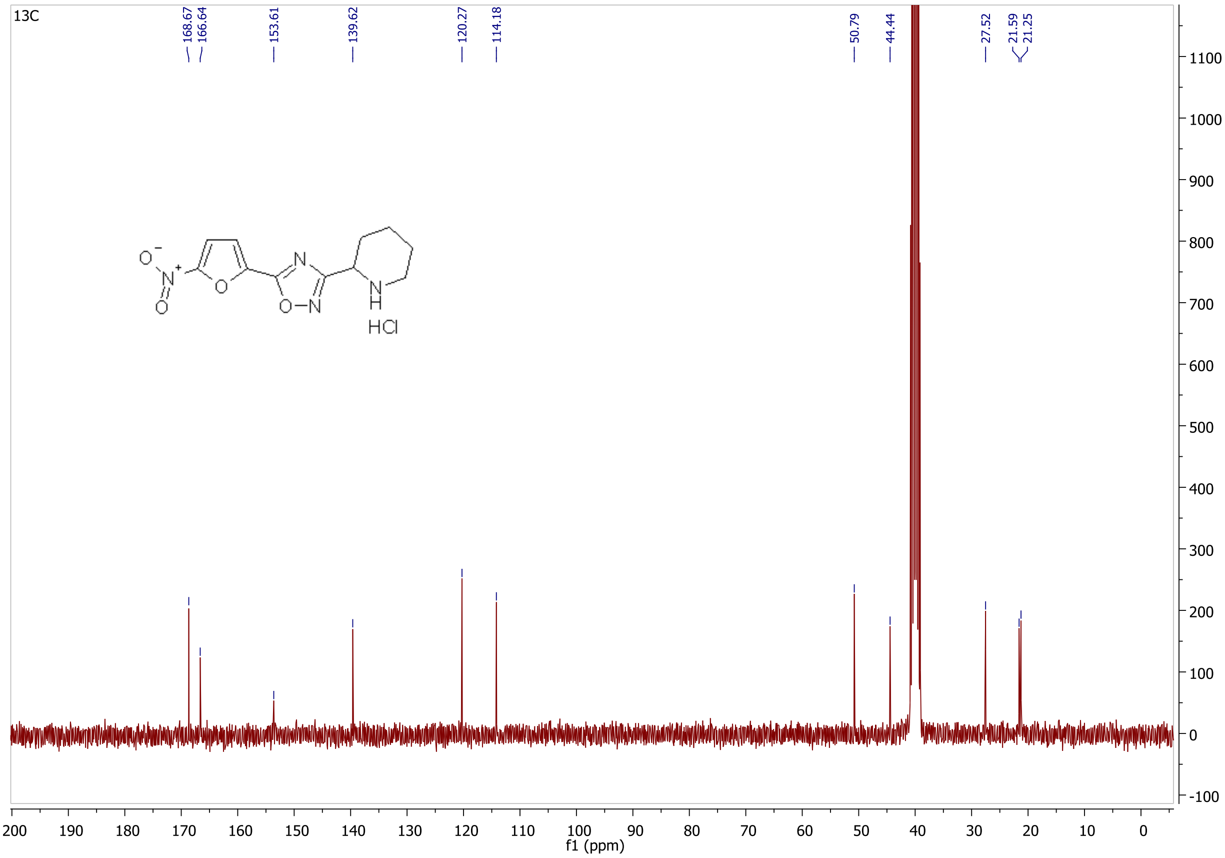

4-methyl-4-[5-(5-nitro-2-furyl)-1,2,4-oxadiazol-3-yl]piperidine hydrochloride (2j)

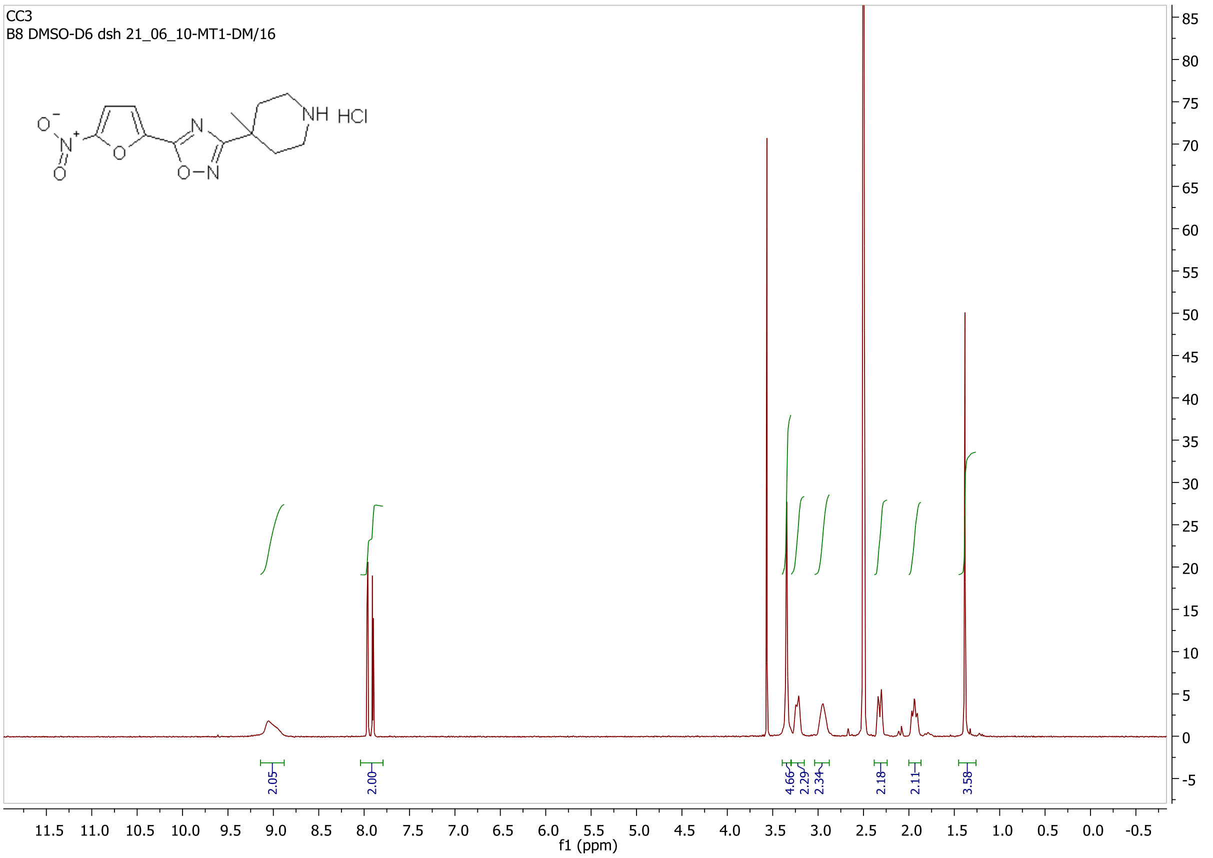

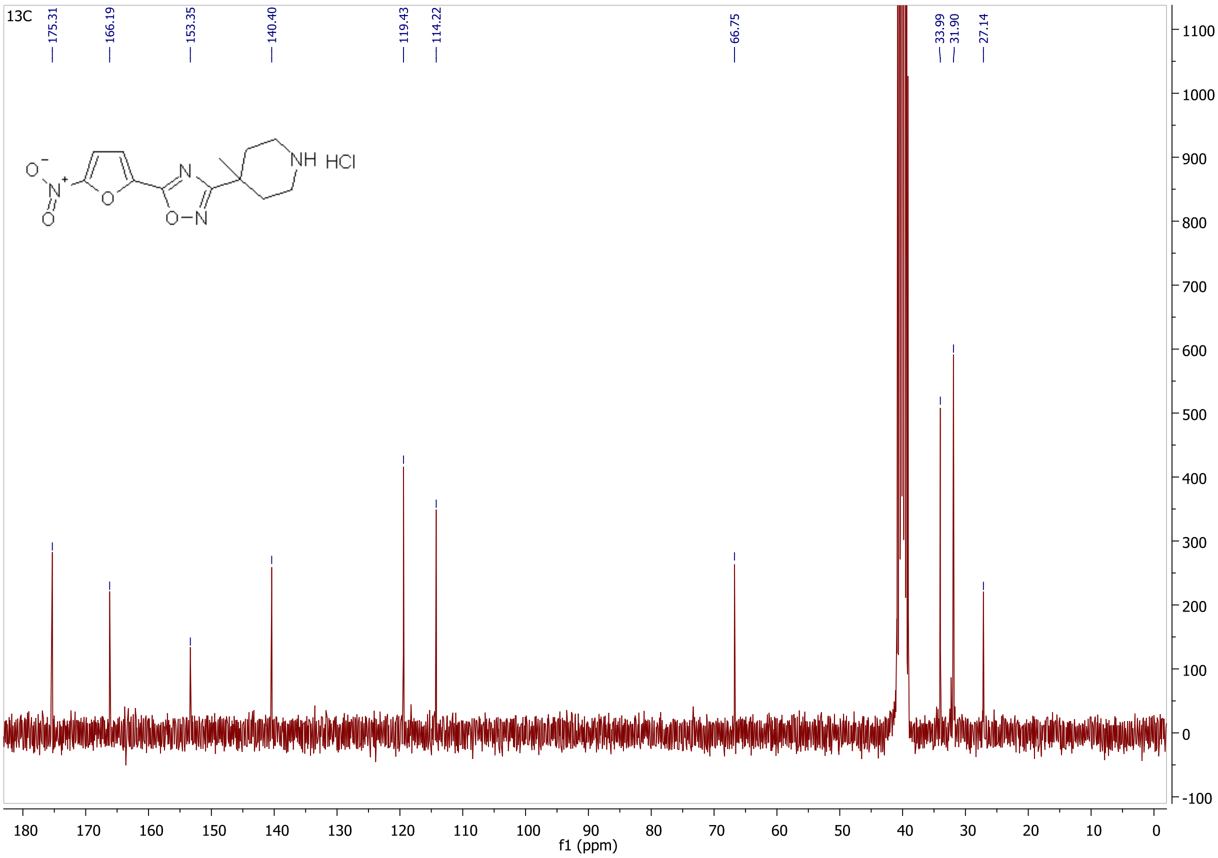

5-(5-nitro-2-furyl)-3-(pyrrolidin-3-ylmethyl)-1,2,4-oxadiazole hydrochloride (2k)

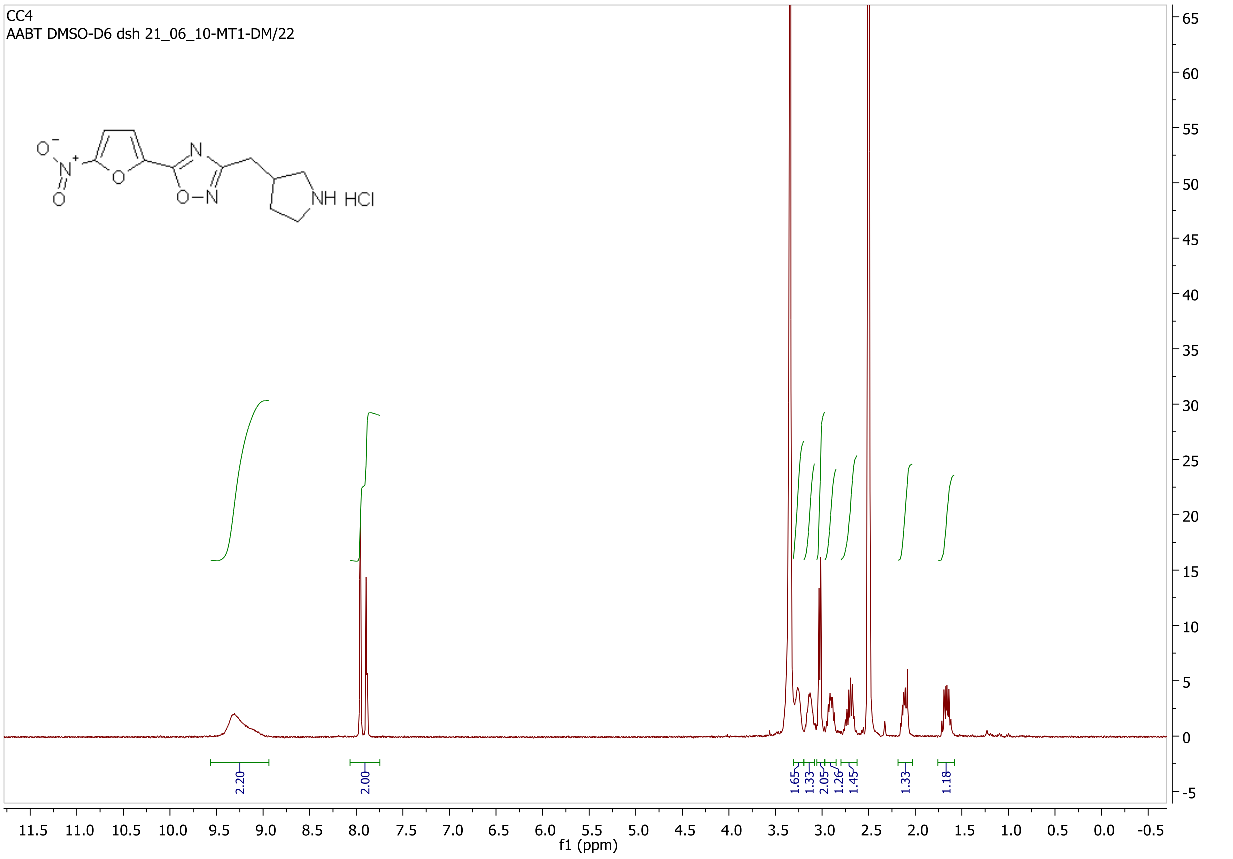

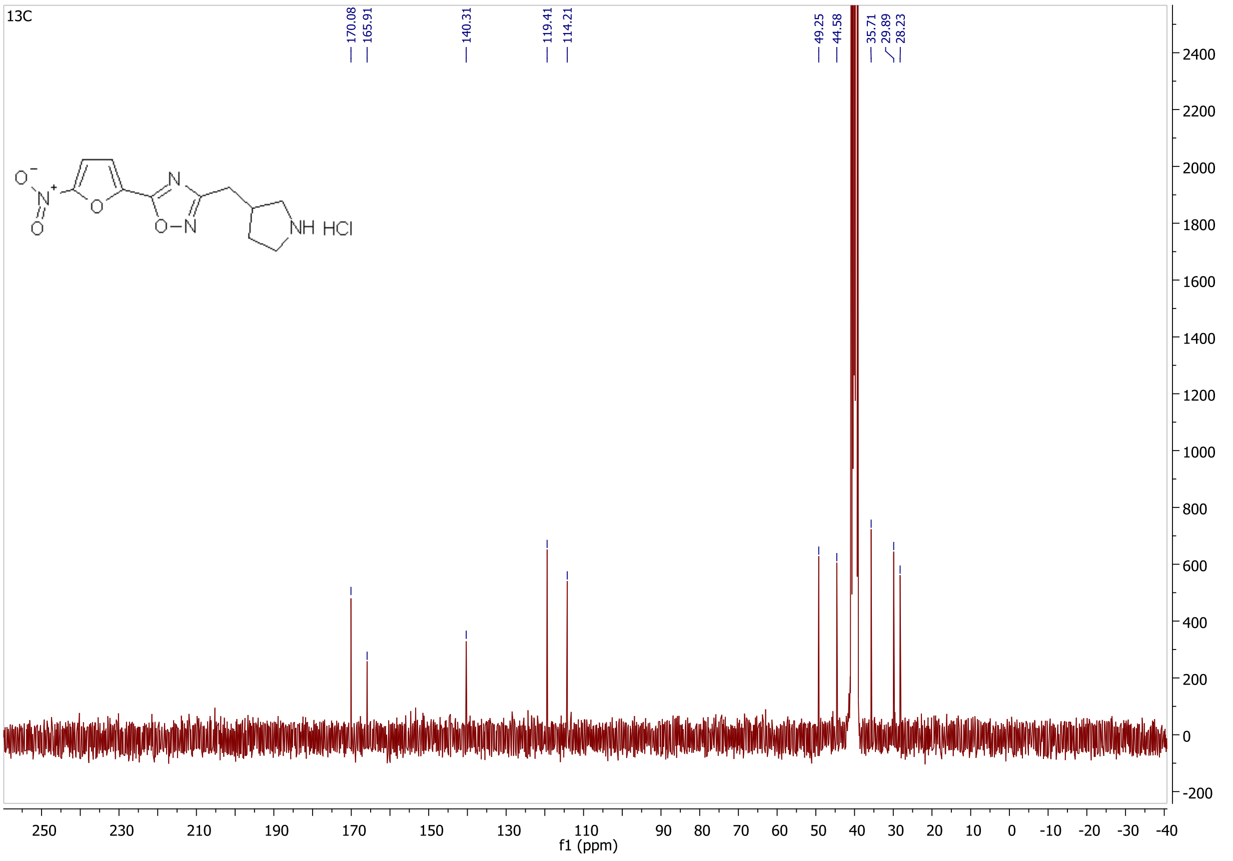

5-(5-nitro-2-furyl)-3-pyrrolidin-3-yl-1,2,4-oxadiazole hydrochloride (2l)

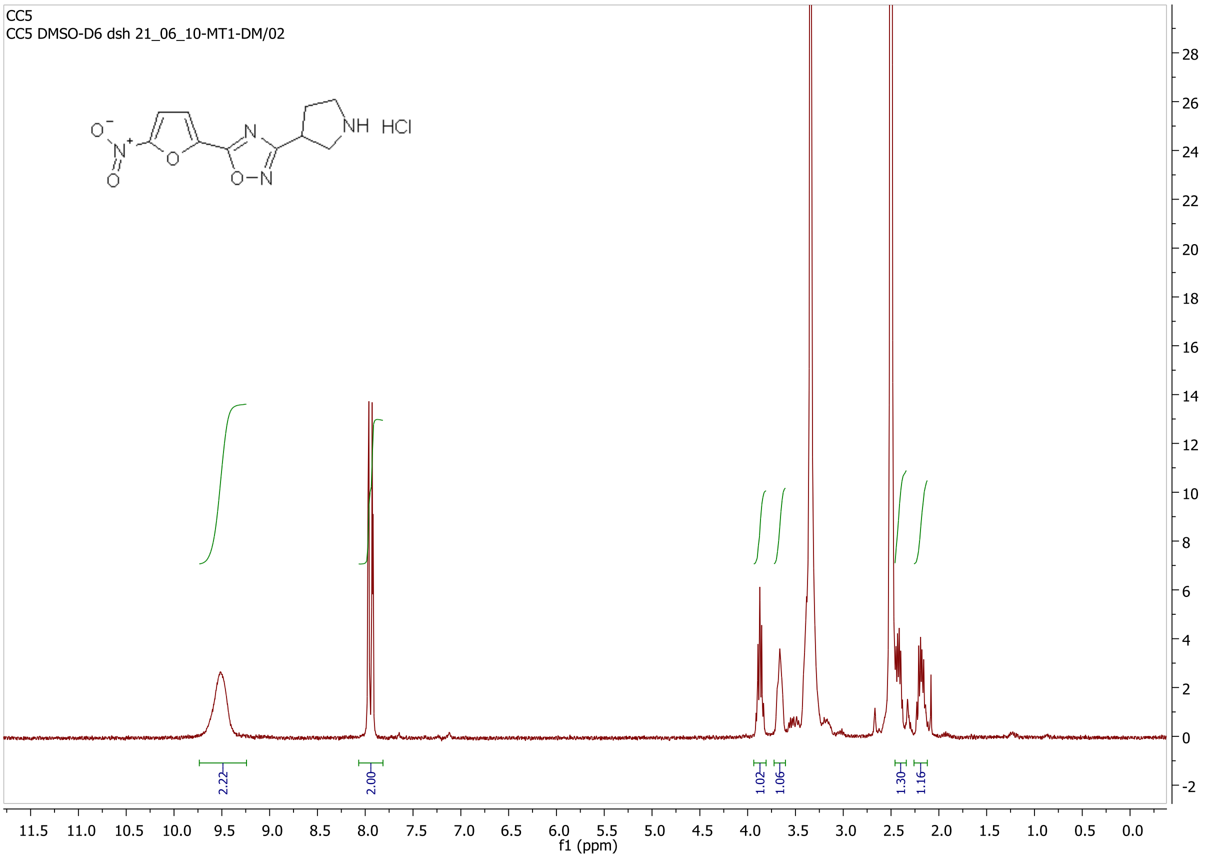

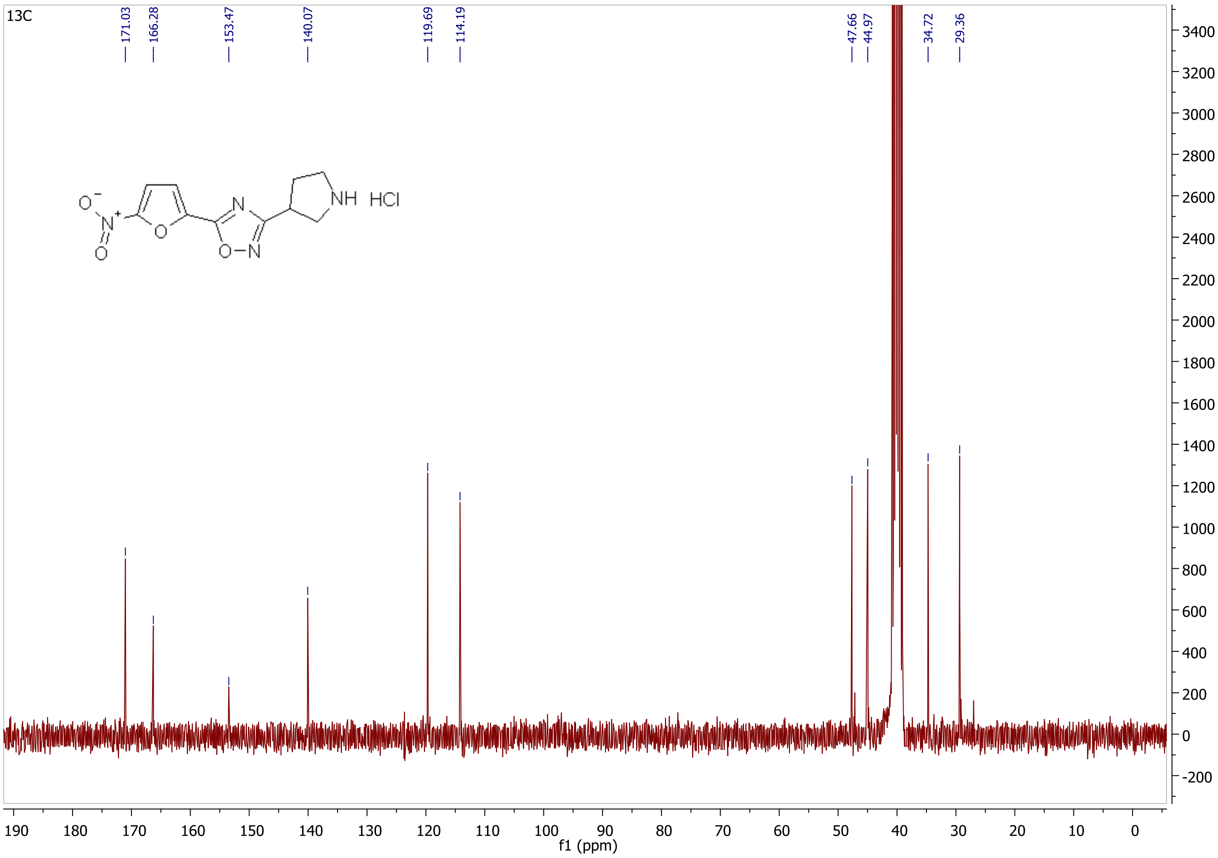

3-(2-methoxyethyl)-4-[5-(5-nitro-2-furyl)-1,2,4-oxadiazol-3-yl]piperidine hydrochloride (2m)

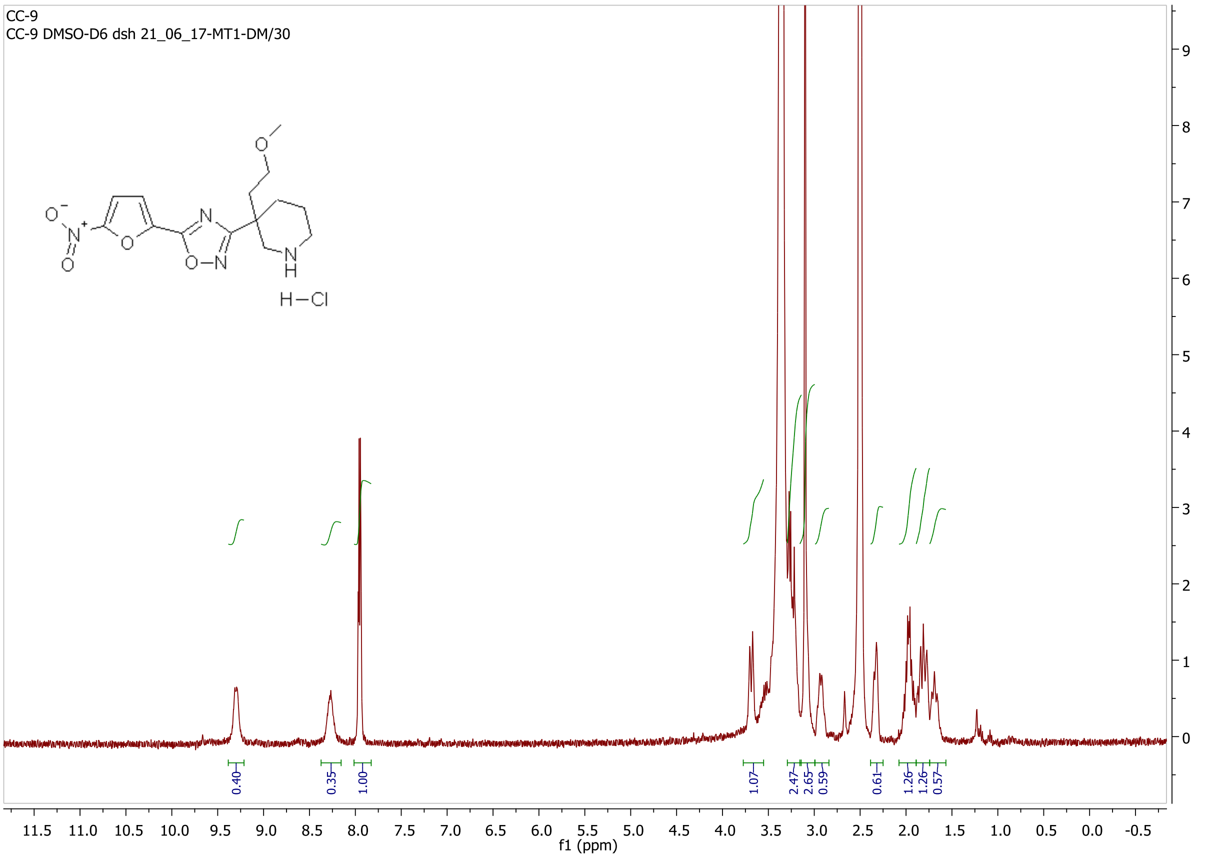

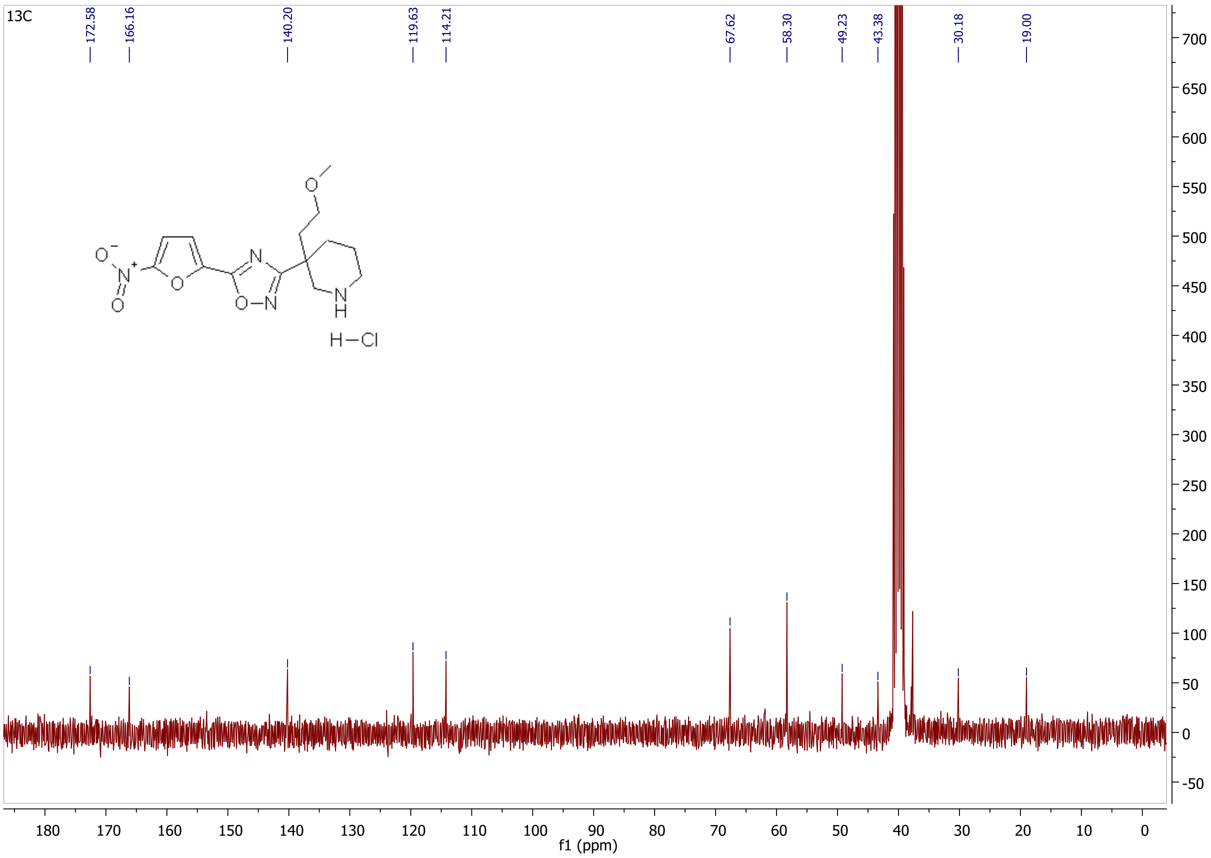

Supplement: Supplementary file 1 [file molecules-29-03364-s001.zip › molecules-3050135-supplementary-S1.pdf]
