# Supplementary material for: Molecular Periphery Design Allows Control of the New Nitrofurans Antimicrobial Selectivity
Source: Molecules. 2024 Jul 17;29(14):3364. doi: 10.3390/molecules29143364 (PMC11279955; doi:10.3390/molecules29143364)
Supplement: Supplementary file 1 [file molecules-29-03364-s001.zip › molecules-3050135-supplementary-S2.pdf]

Supporting Information

Molecular Periphery Design Allows Control Of The New Nitrofurans Antimicrobial Selectivity

Lyubov Vinogradova [1], Alexey Lukin [1], Kristina Komarova [1], Maxim Zhuravlev [1], Artem Fadeev [1], Mikhail Chudinov [1,*], Elizaveta Rogacheva [2], Lyudmila Kraeva [2], Maxim Gureev [3], Yuri Porozov [4,5], Marine Dogonadze [6] and Tatiana Vinogradova [6]

[1] Lomonosov Institute of Fine Chemical Technologies, MIREA—Russian Technological University, Moscow 119454, Russia
[2] Pasteur Institute of Epidemiology and Microbiology, Saint Petersburg 197101, Russia
[3] Institute of Cytology, Russian Academy of Sciences, Tikhoretsky Ave. 4, Saint Petersburg 194064, Russia
[4] Laboratory of Angiopathology, The Institute of General Pathology and Pathophysiology, 8 Baltiyskaya Street, 125315 Moscow, Russia
[5] Advitam Laboratory, Mihaila Shushkaloviħa 13, 11030 Belgrade, Serbia
[6] Saint-Petersburg State Research Institute of Phthisiopulmonology of the Ministry of Healthcare of the Russian Federation, Saint Petersburg 191036, Russia
* Correspondence: chudinov@mirea.ru

Molecular Modeling

Table S1. Target protein structures selected from the RCSB Protein Data Bank. 2

Figure S1. Ligand interactions diagrams of 2c with azoreductase (potential principal target) and NfsB (least likely target for ligand binding). 3

Figure S2. Ligand interactions diagram of reference compound 2~{S})-2-(5-nitrofuran-2-yl)-2,3,5,6,7,8-hexahydro-1~{H}-[1]benzothiolo[2,3-d]pyrimidin- 4-one, complexed with Pgk. 4

Figure S3. Interaction of 2a with the most likely targets in P. aeruginosa. 5

Figure S4. Ligand interactions diagrams of 2c with azoreductase (potential principal target) and NfsB (least likely target for ligand binding). 6

Table S2. Scoring function value for the affinity of the tested compounds against AzoR S.Aureus. 7

Figure S5. A-D: FMN availability for the nitro group in the AzoR structure of S. aureus; E: binding pose of control (green), 2c (yellow) and 2d (orange) in the background of AzoR S. Aureus. 7

Table S3. Scoring function value for the affinity of the tested compounds towards AzoR of P. aeruginosa. 8

Figure S6 .FMN accessibility for the nitro group in the AzoR structure of P. aeruginosa (FMN in green). 8

Table S4. Scoring function value for the affinity of the tested compounds towards AzoR of A. baumannii. 9

Figure S7 .FMN accessibility for the nitro group in the AzoR structure of P. aeruginosa (FMN in green). 9

Figure S8. Comparison of ligand interactions diagrams of 2c and 2d with NfsA. 10

Figure S9. Comparison of ligand interactions diagrams of 2g and 2h with NfsA. 11

Figure S10. Ligand interactions diagrams for compounds 2f (A), 2i (B) and 2k (C) with S.aureus NfsB. 12

Figure S11. Redocking of control structures into the active cavity of the M. tuberculosis proteins. 13

Figure S12. Compounds 2a, 2d, 2e and 2g in complex with InhA 14

Figure S13. Compounds 2a, 2d, 2e and 2g in complex with TBNAT 15

References 16

Table S1. Target protein structures selected from the RCSB Protein Data Bank.

Microorganism	Target PDB ID				
	AzoR	NfsA	NfsB	Pgk	Ddn
P. aeruginosa	3R6W [7]	AF-A0A241XMV5-F1	AF-A0A653BCW6-F1	AF-Q9I5Y4-F1	-
S.aureus	AF-A8Z0H0-F1	AF-Q2G0Z5-F1	7JH4 [27]	4DG5	-
A.baumannii	AF-A0A8B4N120-F1	AF-A0A0M1I6A0-F1	AF-A0A385EYF8	5BT8 [40]	-
M. tuberculosis	-	-	-	-	3R5L [41]

Figure S1. Ligand interactions diagrams of 2c with azoreductase (potential principal target) and NfsB (least likely target for ligand

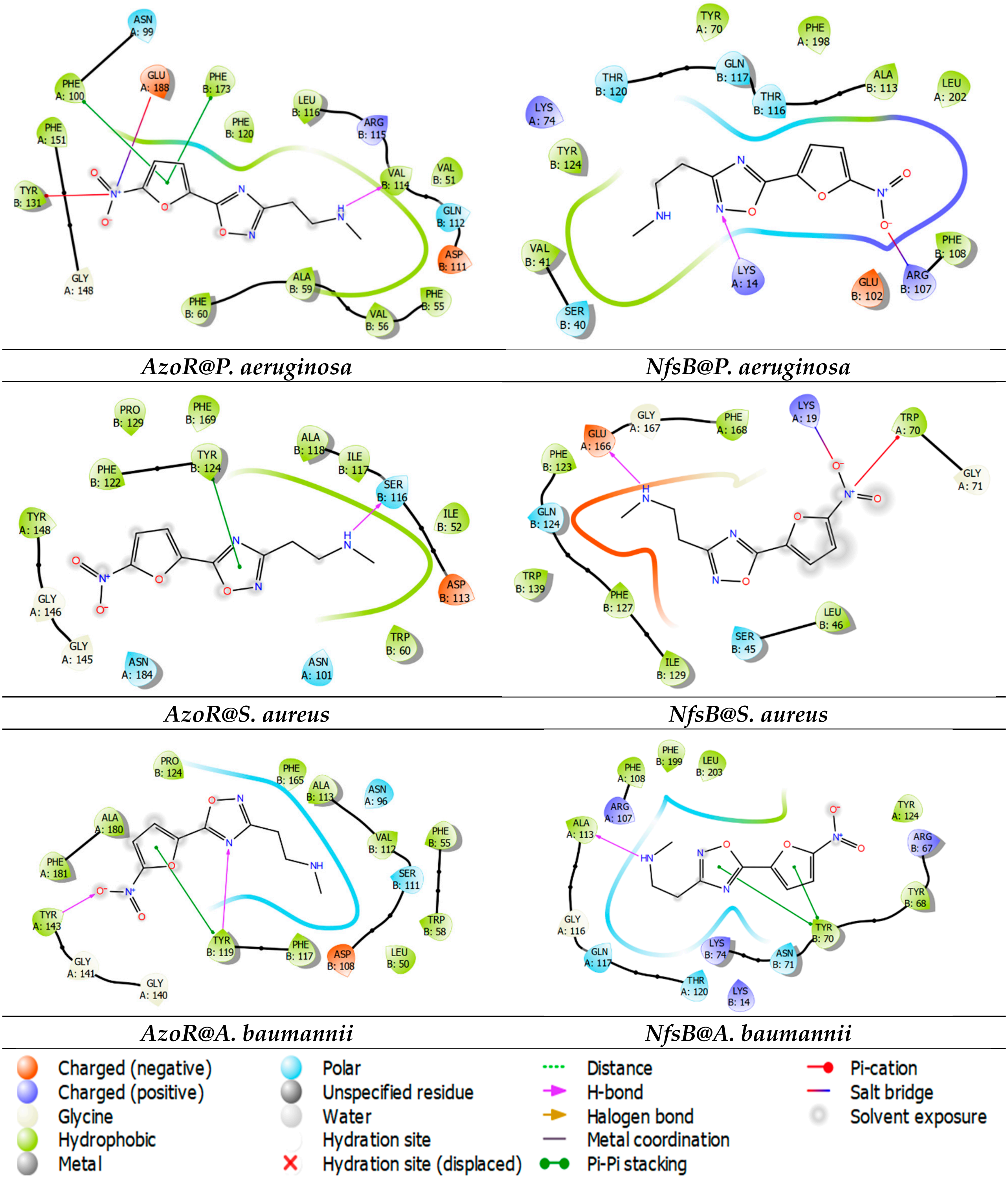

Figure S2. Ligand interactions diagram of reference compound 2~{S})-2-(5-nitrofuran-2-yl)-2,3,5,6,7,8-hexahydro-1~{H}-[1]benzothiolo[2,3-d]pyrimidin-4-one, complexed with Pgk.

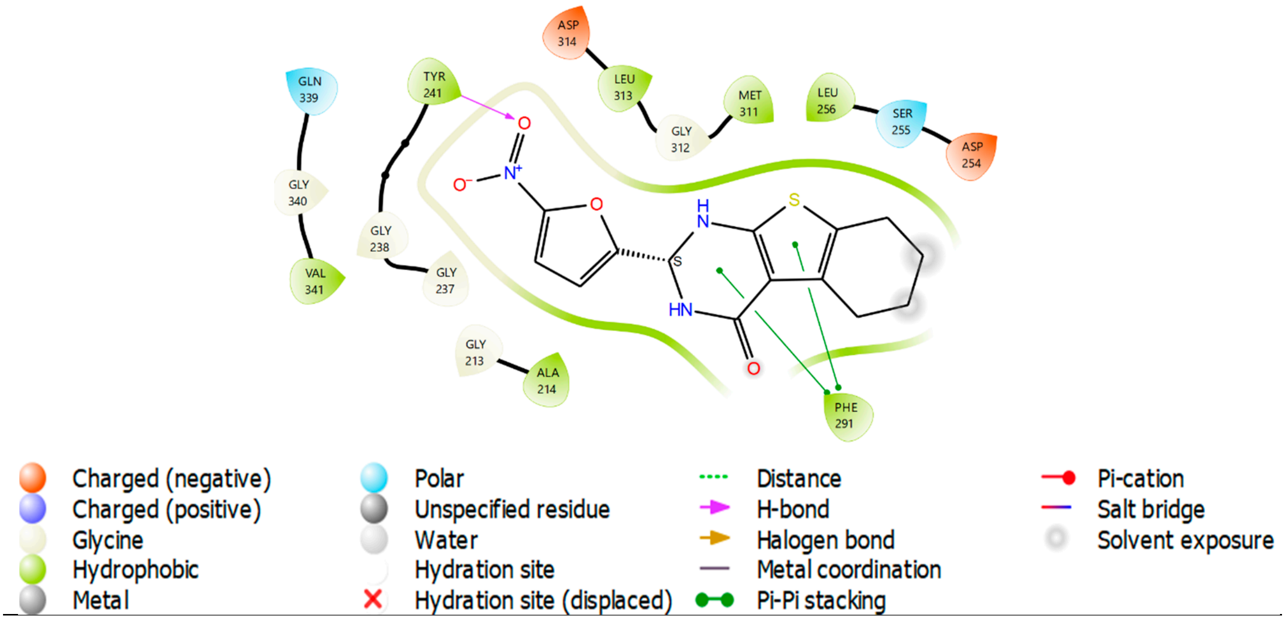

Figure S3. Interaction of 2a with the most likely targets in *P. aeruginosa*.

Three-dimensional model describing the ligand-protein complex, diagrams describing the ligand-protein interactions within the complexes. The compound 2a is shown in gray on the three-dimensional models. The control compound nitrofurazone is shown in green.

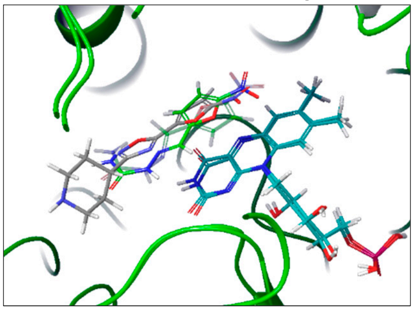

2a@AzoR

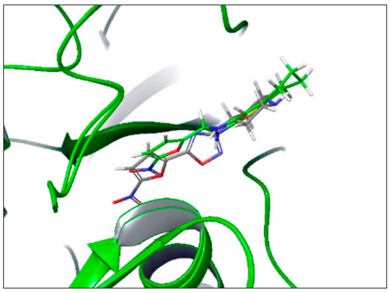

2a@Pgk

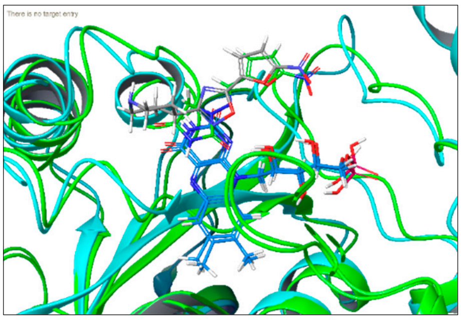

2a@NfsA

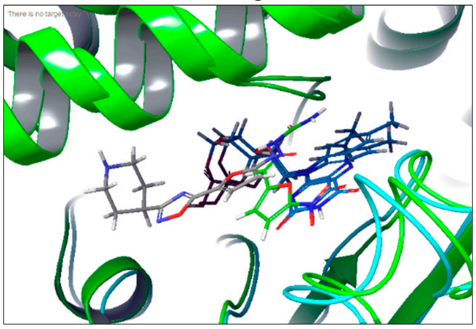

2a@NfsB

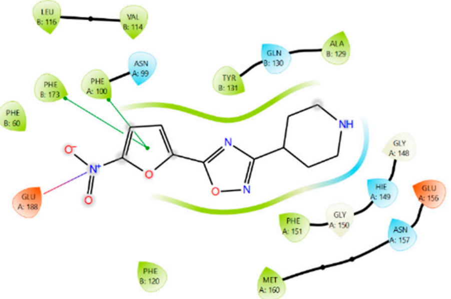

2a@AzoR

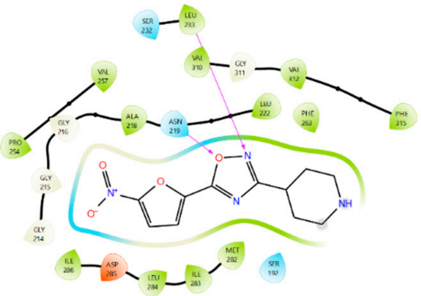

2a@Pgk

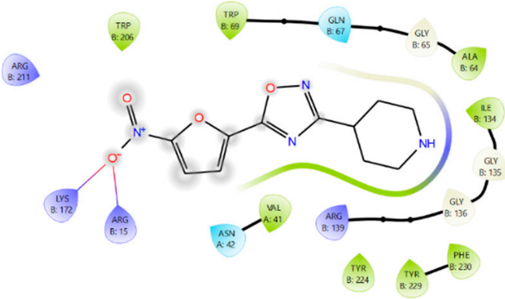

2a@NfsA

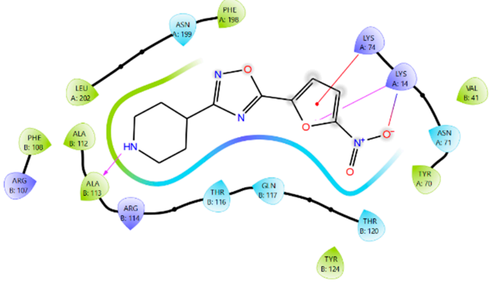

2a@NfsB

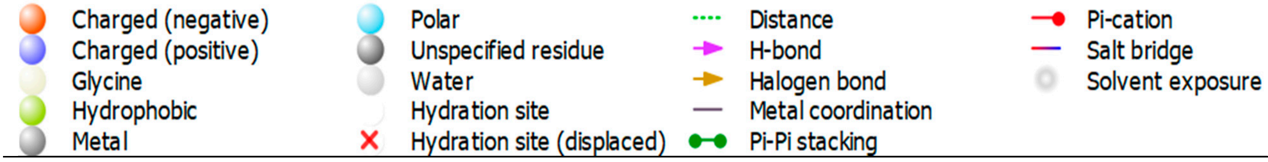

Figure S4. Ligand interactions diagrams of 2c with azoreductase (potential principal target) and NfsB (least likely target for ligand binding).

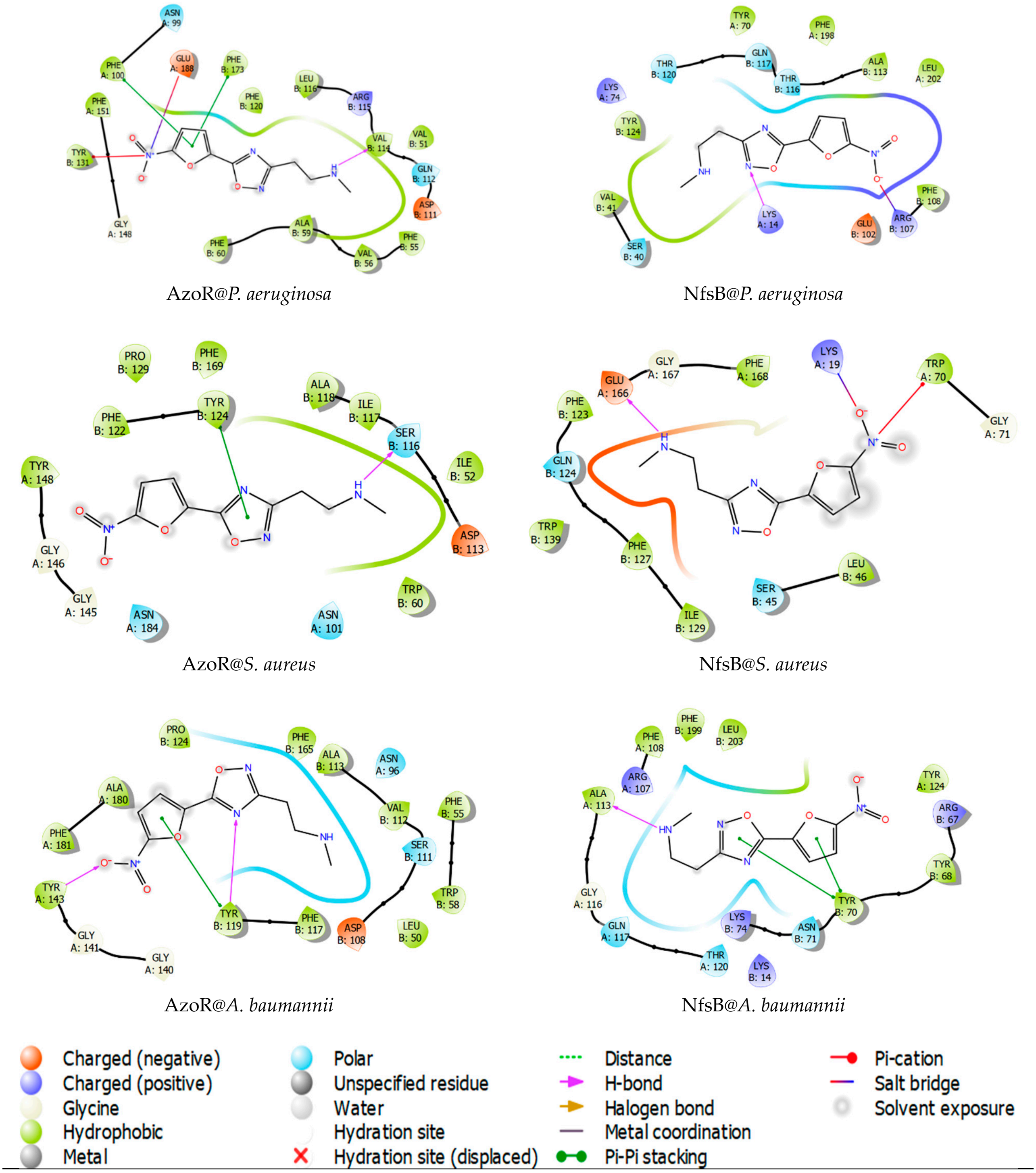

Table S2. Scoring function value for the affinity of the tested compounds against AzoR *S.Aureus*.

Increment of lipophilic interactions, and quality of binding pose (reproduction of pharmacophore characteristics, where ***- complete, **- partial, *- absent).

Compound	AzoR@ *S. aureus*	
	GScore (kcal/mol)	Lipo (kcal/mol)
**2c**	-7.53 (***)	-2.24
**2d**	-6.81 (*)	-1.70
**2g**	-8.64 (*)	-3.97
**2h**	-7.11 (***)	-2.27

Figure S5. A-D: FMN availability for the nitro group in the AzoR structure of *S. aureus*; E: binding pose of control (green), 2c (yellow) and 2d (orange) in the background of AzoR *S. Aureus*.

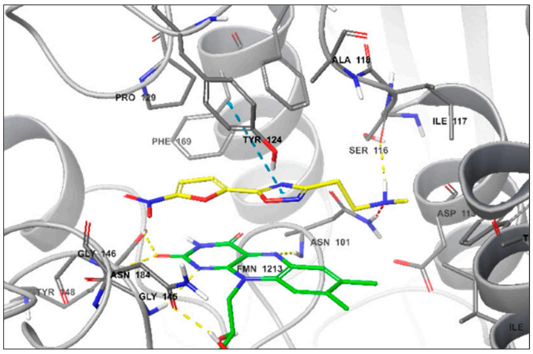

A (**2c**)

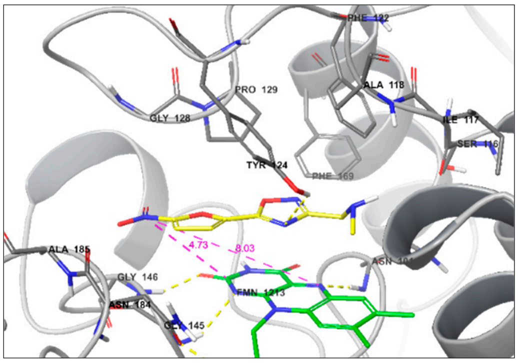

B (**2d**)

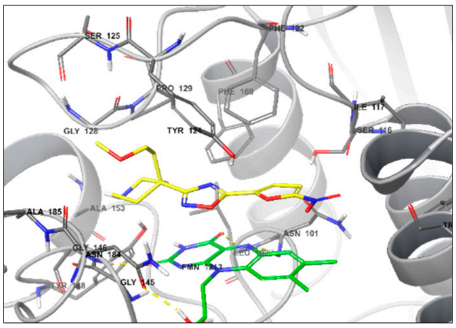

C (**2g**)

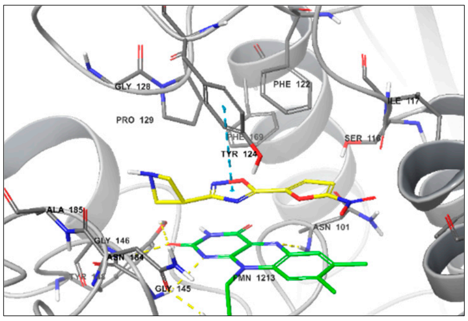

D (**2h**)

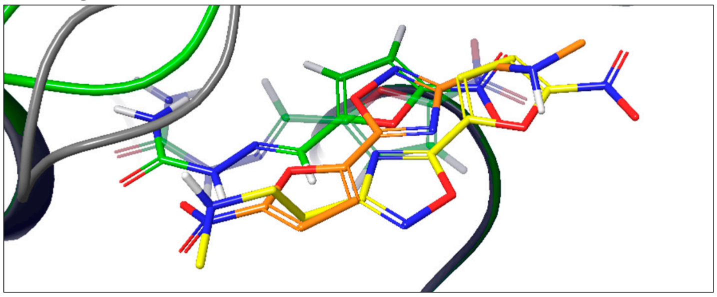

E

Table S3. Scoring function value for the affinity of the tested compounds towards AzoR of *P. aeruginosa*.

Increment of lipophilic interactions, stacking quality (reproduction of pharmacophore characteristics, where *** - complete, ** - partial, * - absent).

compound	AzoR@ *P. aeruginosa*	
	GScore (kcal/mol)	Lipo (kcal/mol)
**2c**	-6,95 (***)	-2,36
**2b**	-6,58 (*)	-1,56
**2d**	-7,59 (*)	-3,03
**2e**	-7,46 (***)	-2,59

Figure S6 .FMN accessibility for the nitro group in the AzoR structure of *P. aeruginosa* (FMN in green).

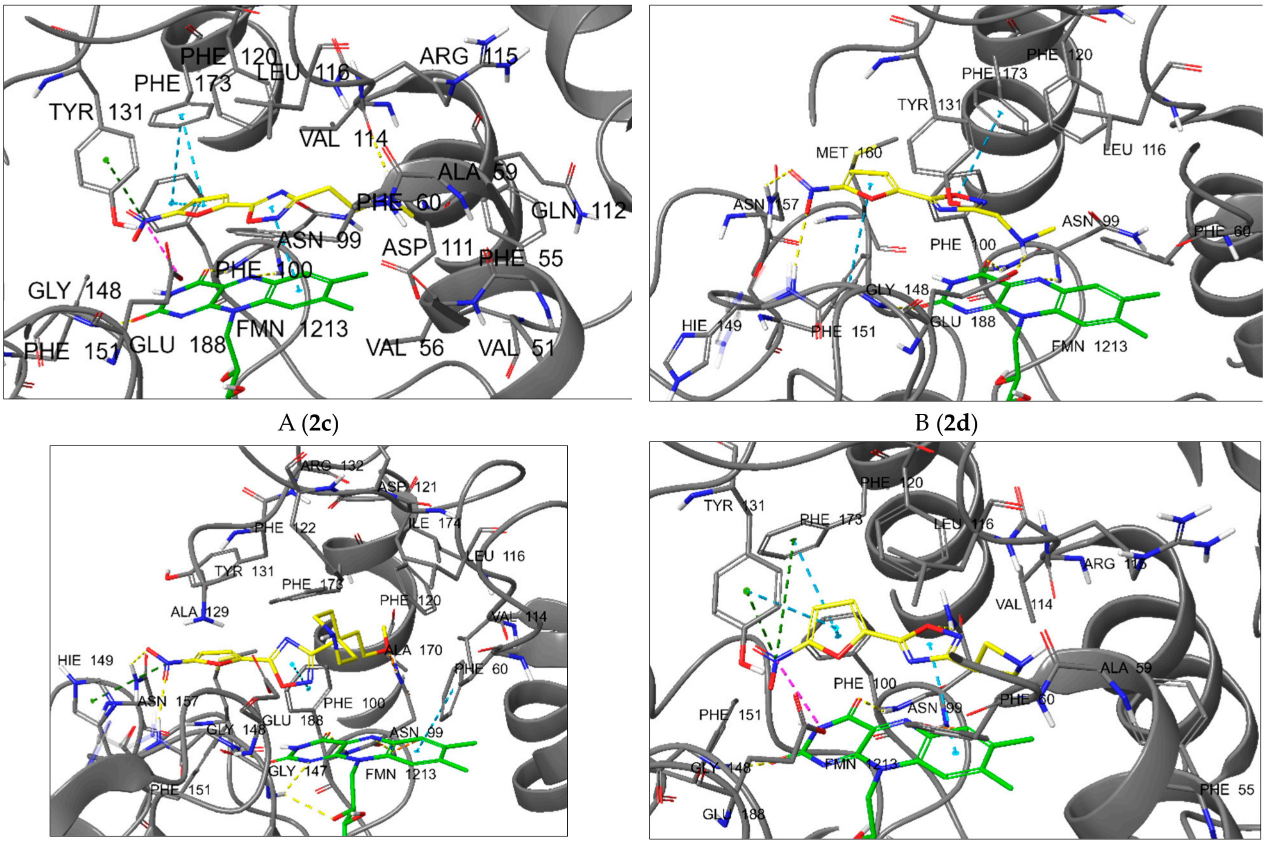

A (**2c**) B (**2d**)

C (**2g**) D (**2h**)

Table S4. Scoring function value for the affinity of the tested compounds towards AzoR of *A. baumannii*.

Increment of lipophilic interactions, stacking quality (reproduction of pharmacophore characteristics, where *** - complete, ** - partial, * - absent).

compound	AzoR@ *A. baumannii*	
	GScore (kcal/mol)	Lipo (kcal/mol)
**2c**	-8,00(***)	-2,21
**2b**	-6,69 (*)	-1,63
**2d**	-7,24 (*)	-3,13
**2e**	-7,48 (***)	-2,51

Figure S7 .FMN accessibility for the nitro group in the AzoR structure of *P. aeruginosa* (FMN in green).

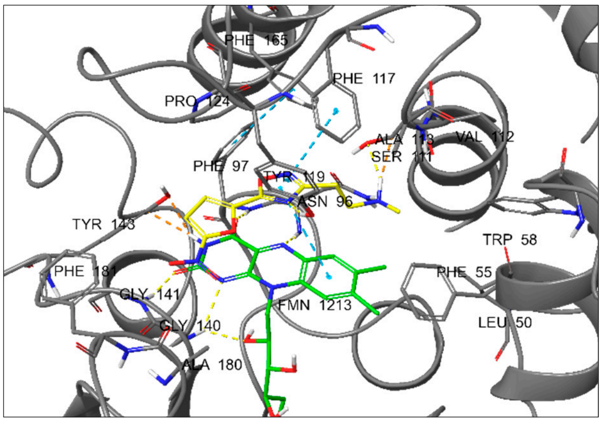

A (**2c**)

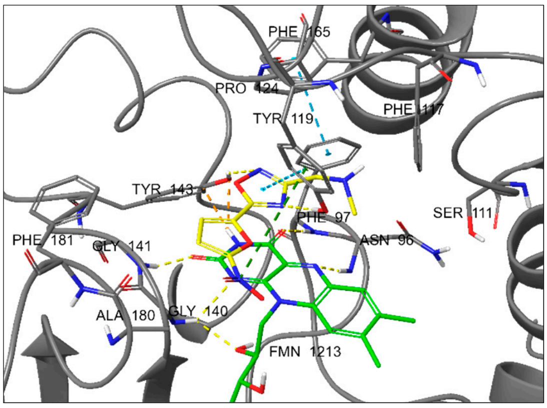

B (**2d**)

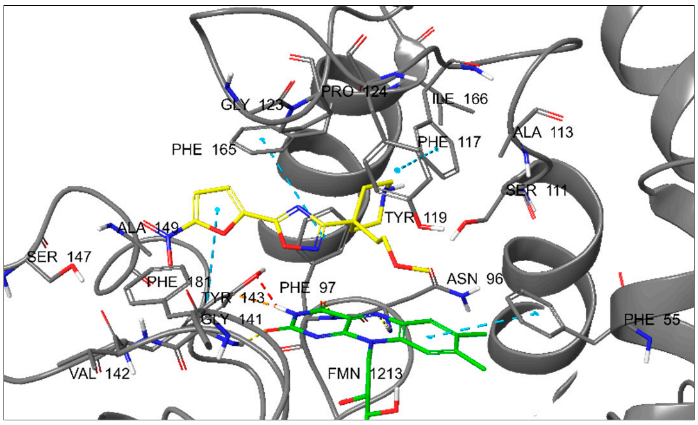

C (**2g**)

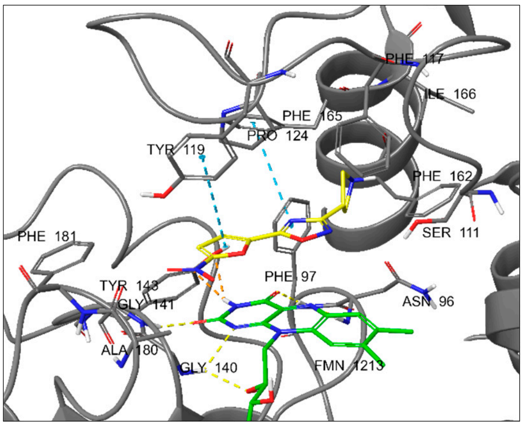

D (**2h**)

Figure S8. Comparison of ligand interactions diagrams of 2c and 2d with NfsA.

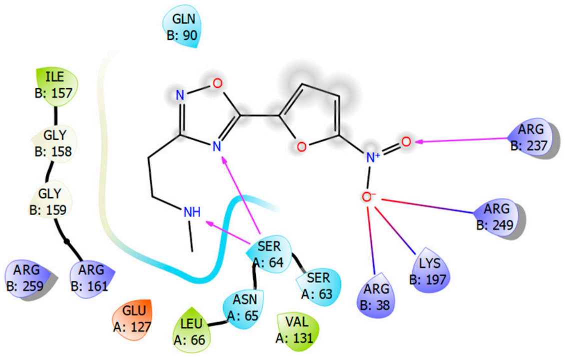

2c - NfsA@*P. aeruginosa*

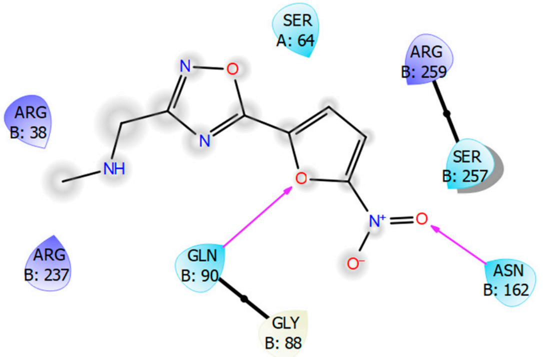

2d - NfsA@ *P. aeruginosa*

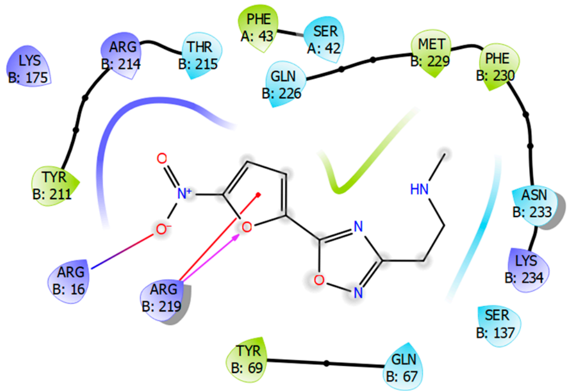

2c - NfsA@*S.aureus*

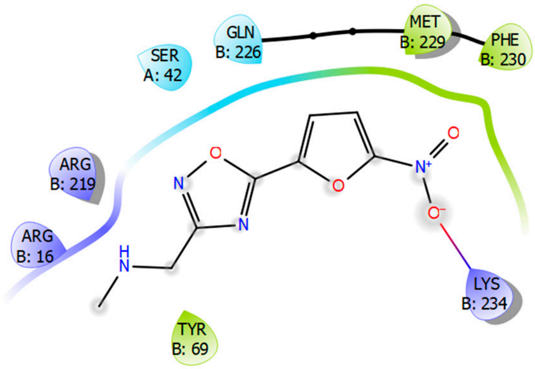

2d - NfsA @*S.aureus*

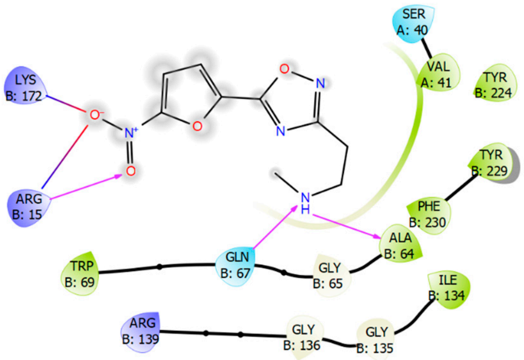

2c - NfsA@*A.baumannii*

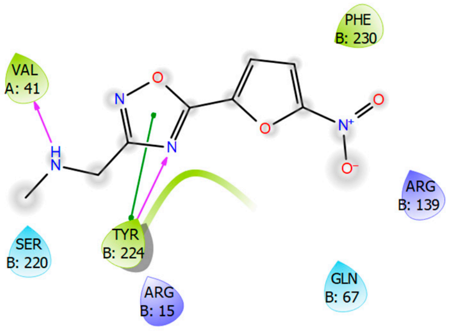

2d - NfsA @*A.baumannii*

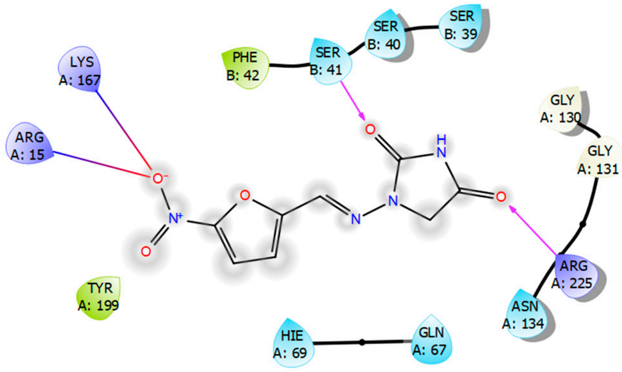

Control (nitrofurantoin – NfsA)

Figure S9. Comparison of ligand interactions diagrams of 2g and 2h with NfsA.

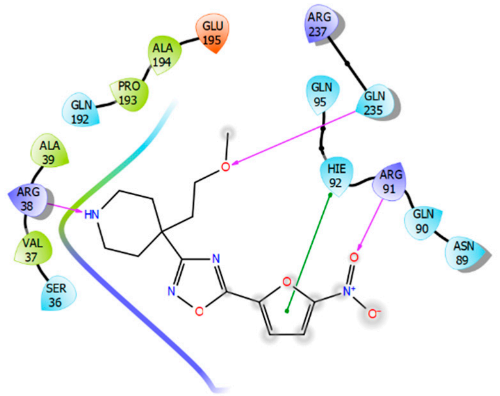

2g - NfsA@*P. aeruginosa*

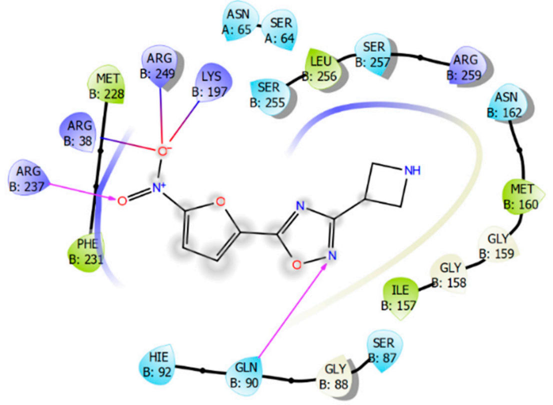

2h - NfsA@ *P. aeruginosa*

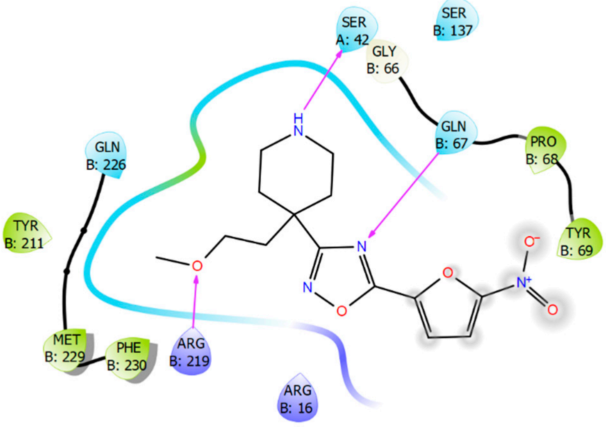

2g - NfsA@*S.aureus*

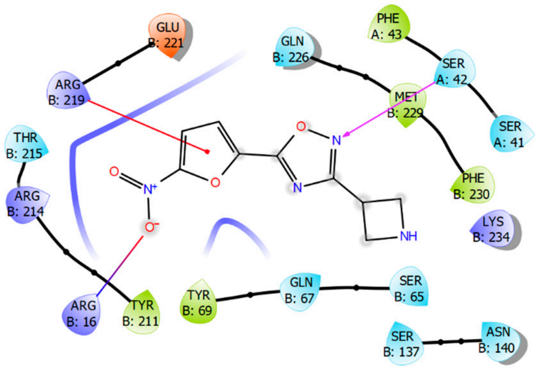

2h - NfsA @*S.aureus*

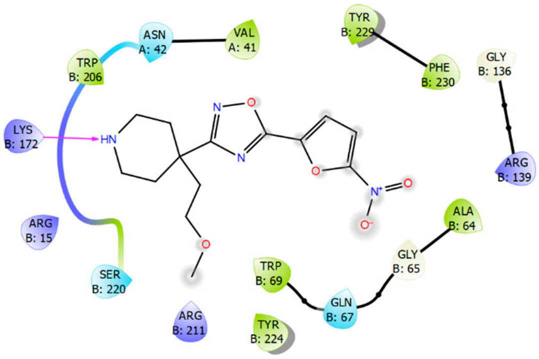

2g - NfsA@*A.baumannii*

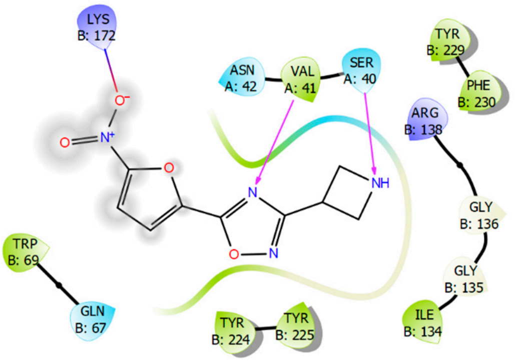

2h - NfsA @*A.baumannii*

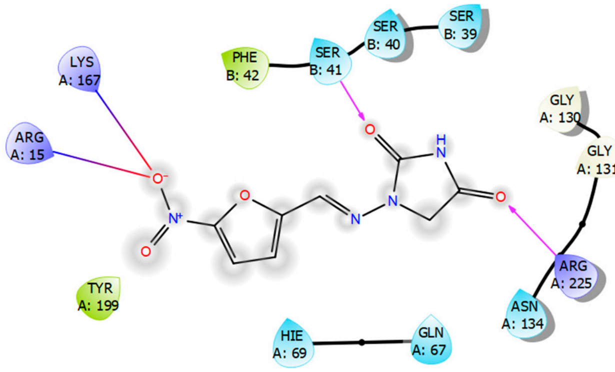

Control (nitrofurantoin – NfsA)

Figure S10. Ligand interactions diagrams for compounds 2f (A), 2i (B) and 2k (C) with *S.aureus* NfsB.

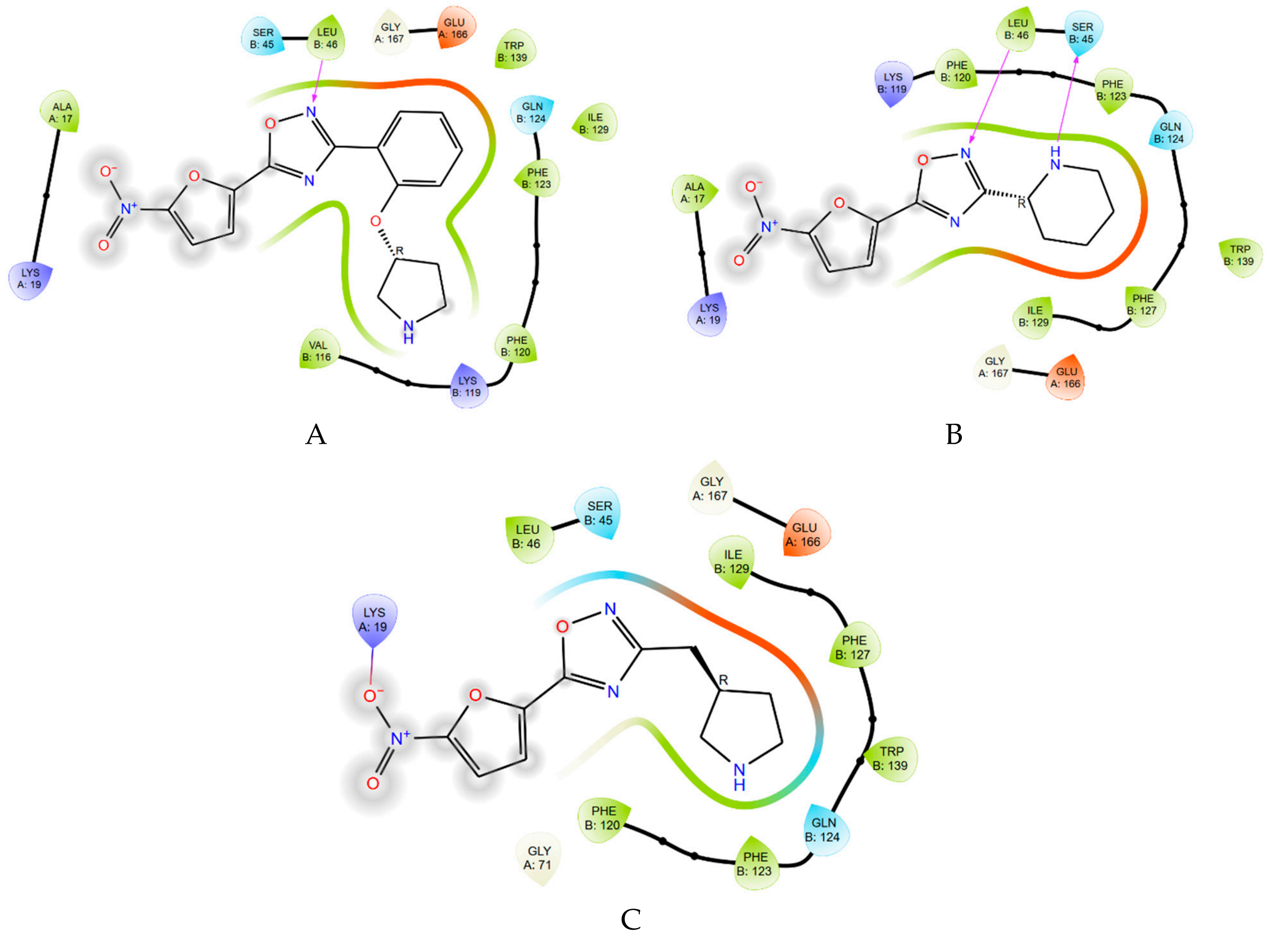

Figure S11. Redocking of control structures into the active cavity of the *M. tuberculosis* proteins.

The green structure represents the results obtained from PDB X-ray analysis, while the yellow structure shows the binding pose obtained through molecular docking.

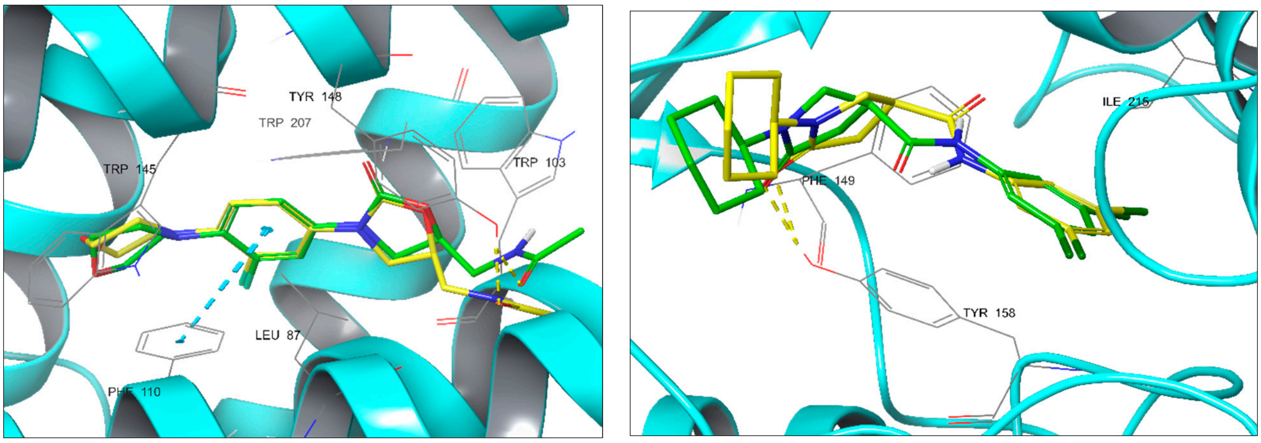

EthR InhA

Figure S12. Compounds 2a, 2d, 2e and 2g in complex with InhA.

The green structures represent the control data from PDB (X-RAY results). The yellow dashed lines represent hydrogen bonding, the blue dashed lines represent π-stacking interactions, and the orange dashed lines represent tensegrity contacts.

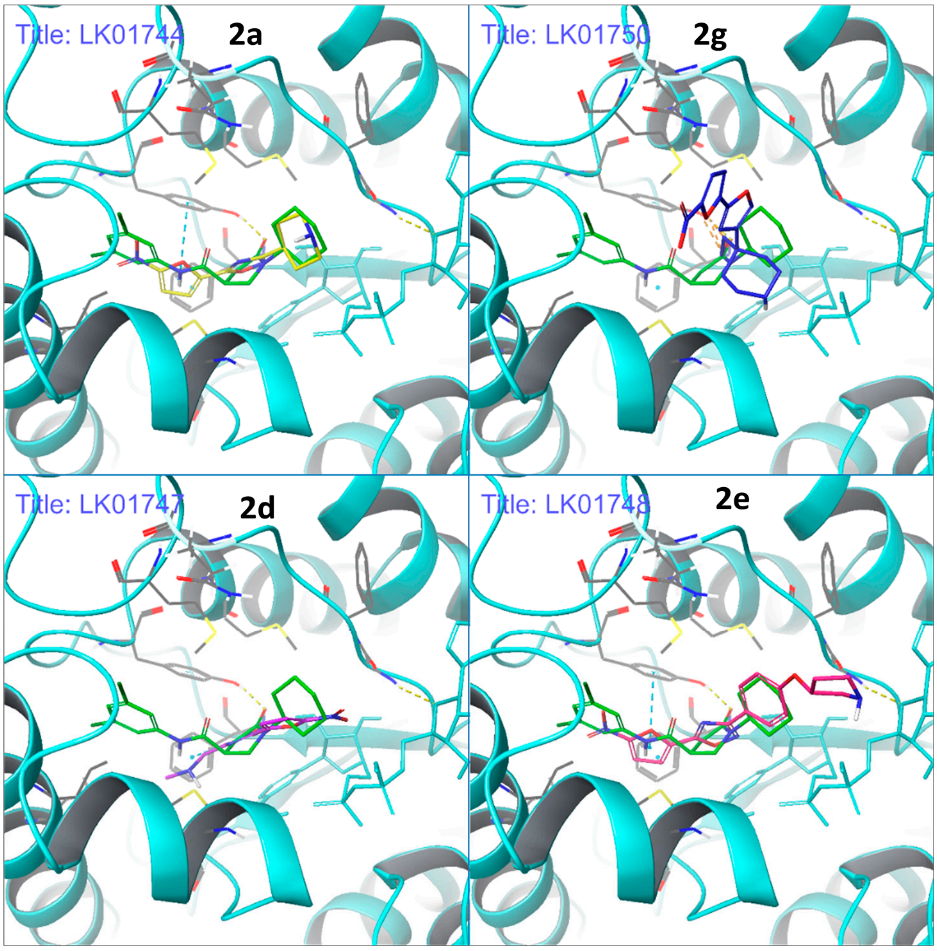

Figure S13. Compounds 2a, 2d, 2e and 2g in complex with TBNAT.

Dashed lines: yellow - hydrogen bonding, blue - π-stacking interactions, orange - strained contacts.

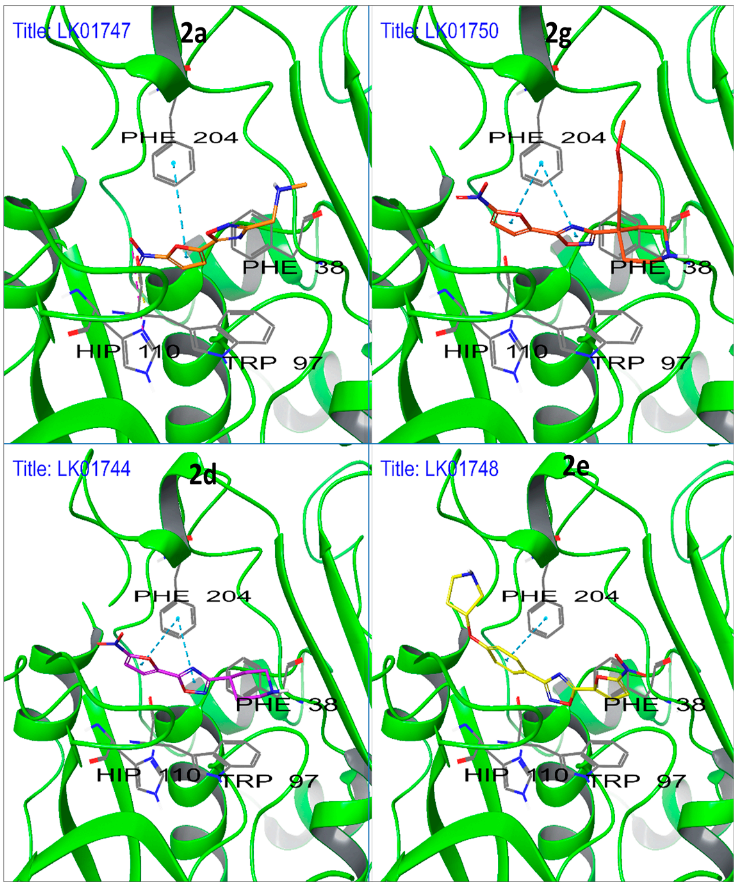

References

7. Ryan, A.; Kaplan, E.; Laurieri, N.; Lowe, E.; Sim, E. Activation of nitrofurazone by azoreductases: multiple activities in one enzyme. *Scientific reports* **2011**, *1*, 63, doi:10.1038/srep00063.
27. O'Neill, A.G.; Beaupre, B.A.; Zheng, Y.; Liu, D.; Moran, G.R. NfoR: Chromate Reductase or Flavin Mononucleotide Reductase? *Appl Environ Microbiol* **2020**, *86*, doi:10.1128/AEM.01758-20.
40. Baretta, K.; Garen, C.; Yin, J.; James, M.N. Expression, purification, crystallization and preliminary crystallographic analysis of the phosphoglycerate kinase from Acinetobacter baumannii. *Acta Crystallogr Sect F Struct Biol Cryst Commun* **2012**, *68*, 790-792, doi:10.1107/S1744309112020222.
41. Cellitti, S.E.; Shaffer, J.; Jones, D.H.; Mukherjee, T.; Gurumurthy, M.; Bursulaya, B.; Boshoff, H.I.; Choi, I.; Nayyar, A.; Lee, Y.S.; et al. Structure of Ddn, the deazaflavin-dependent nitroreductase from Mycobacterium tuberculosis involved in bioreductive activation of PA-824. *Structure* **2012**, *20*, 101-112, doi:10.1016/j.str.2011.11.001.
